# Systematic literature reviews to identify epidemiological, clinical, economic and health-related quality of life evidence in activated PI3Kδ syndrome (APDS)

**DOI:** 10.1186/s12865-025-00723-6

**Published:** 2025-07-19

**Authors:** Katerina Vlachopoulou, Joanne Tutein Nolthenius, Jo Luscombe, Jessica Radford, Keval Haria, Faye Bolan, Sirah Bah

**Affiliations:** 1 Pharming Group N.V., Leiden, The Netherlands; 2 MAP Patient Access Limited, Cambridge, UK; 3 Costello Medical Consulting Limited, London, UK; 4 Costello Medical Consulting Limited, Manchester, UK

**Keywords:** APDS, Inborn errors of immunity, PI3 Kδ, Systematic literature review, Epidemiology systematic literature review, Clinical systematic literature review, Economic systematic literature review, Health-related quality of life systematic literature review.

## Abstract

**Background:**

Activated phosphoinositide 3-kinase delta (PI3Kδ) syndrome (APDS) is an ultra-rare inborn error of immunity, characterised by immunodeficiency and immune dysregulation. Having only been recognised in 2013, evidence on APDS is limited. We carried out four systematic literature reviews (SLRs) to identify and narratively synthesise evidence on the following for APDS: epidemiology (epidemiology SLR), clinical efficacy/safety of treatments (clinical SLR), cost-effectiveness and costs/healthcare (HCRU) associated with (economic SLR) and health-related quality of life (HRQoL) and utility data (HRQoL SLR) from a global perspective.

**Methods:**

The Cochrane Collaboration and the University of York’s Centre for Reviews and Dissemination (CRD) guidelines were followed. MEDLINE, Embase, the Cochrane Library, University of York CRD, conference proceedings and other grey literature were searched through to May 2023 for all SLRs, except the epidemiology SLR (searched to Nov 2021); economic databases were also searched for the economic and HRQoL SLRs. Eligible records were: primary epidemiology publications (epidemiology SLR), interventional/observational studies of treatments (clinical SLR), cost/HCRU studies/economic evaluations (economic SLR) and HRQoL/utility studies (HRQoL SLR) in people with APDS. Risk of bias was assessed using the Downs and Black checklist (clinical SLR) and the Drummond checklist (economic SLR).

**Results:**

The numbers of unique relevant studies identified were: 0 (epidemiology SLR), 117 (clinical SLR; 87 reported on <5 patients), 2 (economic SLR) and 1 (HRQoL SLR). The clinical SLR reported symptomatic treatments to be only partially effective at controlling APDS manifestations, with variable tolerability. Outcome reporting was heterogeneous and inconsistent, with small sample sizes and patients receiving multiple treatments, limiting interpretation of results. The economic SLR reported a high direct cost of APDS. Additional HRQoL/utility studies are required to evaluate the clinical and HRQoL burden of APDS and the impact of therapies.

**Conclusion:**

Four methodologically robust SLRs identified limited evidence on epidemiology, clinical outcomes, costs and HRQoL in APDS, reflecting its ultra-rare nature and recent recognition. This suggests a need for more rigorous data evaluating the clinical and economic effectiveness of APDS treatments. Outcome reporting was highly heterogeneous and inconsistent across studies, sample sizes were small and patients often received multiple treatments, limiting interpretation of results.

**Supplementary Information:**

The online version contains supplementary material available at 10.1186/s12865-025-00723-6.

## Introduction

Activated phosphoinositide 3-kinase delta (PI3 Kδ) syndrome (APDS) is an ultra-rare inborn error of immunity (IEI) characterised by immune deficiency, immune dysregulation, an increased risk of malignancy and increased susceptibility to infections [1–4]. APDS is a progressive condition that was only recognised in 2013 [4], and is caused by variants in genes that encode subunits of the PI3 Kδ kinase complex [2, 5]. The PI3 Kδ pathway is a key signalling pathway that regulates cellular processes such as metabolism, growth, proliferation, differentiation and survival in immune cells [5, 6]. In APDS, variants in the genes of PI3 Kδ subunits cause hyperactive PI3 Kδ signalling [1, 6–9], which leads to severe, complex and potentially life-threatening manifestations that affect multiple organ systems, including the sinopulmonary, lymphatic and gastrointestinal (GI) systems [7, 10, 11]. This wide range of debilitating symptoms in APDS can have a substantial impact on an individual’s quality of life [12–14].

Whilst the range of treatments available for APDS is broad, their effectiveness is limited, with treatments mainly used to address the symptoms of APDS rather than the underlying pathology [8, 15]. Clinical management includes antimicrobial therapies (antibiotics, antifungal and antiviral drugs), immunoglobulin replacement therapy (IRT), surgeries (e.g. splenectomy) and immunosuppressive treatments including steroids (e.g. prednisolone), mammalian target of rapamycin (mTOR) inhibitors (e.g. sirolimus/rapamycin and everolimus) and monoclonal antibodies (e.g. rituximab) [7, 8, 10]. Haematopoietic stem cell transplantation (HSCT) may be a potentially curative option in some individuals; however, this is not the treatment of choice for the majority of affected individuals, due to a high risk of morbidity and mortality, with donor availability and patient comorbidities also limiting transplantation as an option [3, 11, 15, 16]. More recently, small molecule PI3 K inhibitors such as leniolisib, nemiralisib and seletalisib have been studied to examine their potential to reduce the hyperactive signalling of the enzyme, thereby offering a targeted therapy that can address the underlying cause of APDS. Leniolisib, which selectively targets PI3 Kδ, is now approved in the United States (US) by the Food and Drug Administration (FDA), in the United Kingdom (UK) by the Medicines and Healthcare products Regulatory Agency (MHRA) and in Israel by the Ministry of Health (MOH) [9, 17–24].

## Objectives

As an ultra-rare and recently recognised condition, the burden of disease and epidemiology of APDS remains unclear. We conducted four de novo systematic literature reviews (SLRs) to comprehensively examine the existing literature on APDS. The four key objectives of our reviews were to: (1) identify all relevant evidence on the clinical effectiveness and safety of treatments used for APDS (clinical SLR); (2) identify all relevant evidence on the incidence and prevalence of APDS (epidemiology SLR); (3) identify all relevant evidence from economic evaluations and cost and healthcare resource use (HCRU) studies in people with APDS (economic SLR) and (4) identify all relevant evidence on HRQoL and utility in people with APDS (HRQoL SLR).

## Methods

### SLRs

Four de novo SLRs (the clinical, epidemiology, economic and HRQoL SLRs) were conducted on 11^th^ and 12^th^ November 2021. Subsequent updates were conducted for the clinical, economic and HRQoL SLRs on 18^th^ May 2023 to capture all recent, relevant evidence, with hits deduplicated against hits identified in the original SLRs. The SLRs were conducted following current best practices, as recommended by the Cochrane Collaboration and the University of York’s Centre for Reviews and Dissemination (CRD) guidelines [25, 26]. All SLRs followed pre-specified protocols; the only protocol amendment was that articles with non-English full texts were evaluated for eligibility and data extraction based on their English abstracts. The protocols were not registered.

Methods and results of these SLRs are reported in line with the guidance provided by the National Institute for Health and Care Excellence (NICE) and the Preferred Reporting Items for Systematic Reviews and Meta-Analyses (PRISMA) guidelines [27, 28]. Electronic database searches were performed, as well as supplementary searches of grey literature for all the SLRs, including searches of health technology assessment (HTA) agency websites, bibliographies, clinical trial registries and economic websites (for the economic and HRQoL SLRs only). The search terms used for the electronic database and grey literature searches for each SLR are outlined in the Supplementary Methods 1: Search Terms. Each SLR search strategy broadly considered disease-related terms, such as APDS, immunodeficiency and PI3 K.

### Eligibility criteria

Articles were screened for inclusion based on predefined eligibility criteria, such as ensuring studies were in humans with APDS undergoing any treatment, published from 2013 onwards (congresses/congress books [or similar] were searched from 2019 onwards) and reported outcomes of interest for each SLR, such as immunophenotyping measures, prevalence of APDS, cost per clinical outcome and HRQoL measures for the clinical, epidemiology, economic and HRQoL SLRs, respectively. As APDS has only recently been recognised, a broad scope was maintained for the SLRs to ensure all potentially relevant information was captured. The full eligibility criteria for each SLR are described in the Supplementary Methods 2: Eligibility Criteria. For each SLR, titles and abstracts were screened for inclusion by two independent reviewers, with a third reviewer consulted to make the final decision if a consensus could not be reached.

### Data extraction and analysis

Full texts deemed eligible for inclusion were extracted by a single reviewer into pre-specified data extraction tables in Microsoft Word and were checked by a second individual, with a third individual resolving any discrepancies between the two reviewers. The data extraction was performed in line with guidelines from the University of York’s CRD [25, 26]. For the clinical SLR, analysis was performed by grouping the studies by patient numbers (<5 or ≥5) and by treatment type. No standardised metrics or transformation were used for analysis, instead, descriptive synthesis was used to summarise the outcomes most commonly reported across the included studies. The heterogeneity of effect was examined narratively. Each study (excluding case reports, for which quality assessment was not conducted) was assessed for quality by considering the characteristics that could introduce bias, using the Downs and Black checklist, which is applicable to all study types eligible for inclusion in the clinical SLR [29]. One individual completed quality assessment of each study, which was independently verified by a second individual. The results from the quality assessment are outlined in the Supplementary Results 1: Quality Assessment. For economic evaluations in the economic SLR, the quality was assessed using the Drummond checklist [30], completed by one individual and independently verified by another individual, however, no risk of bias assessment was conducted for the cost and HCRU studies in the economic SLR. Additionally, no formal assessment of risk of bias was performed for the epidemiology or HRQoL SLRs. No certainty, sensitivity or statistical analyses were performed due to the anticipated heterogeneity of the included studies.

## Results

### Summary of included studies

Across all SLRs, a total of 120 unique studies published between 2013 and 2023 (2021 for the epidemiology SLR) were included, consisting primarily of studies included in the clinical SLR (*n*= 117/120, 97.5%) [3, 7, 9, 11, 16, 22, 31–150].

**Fig. 1:**
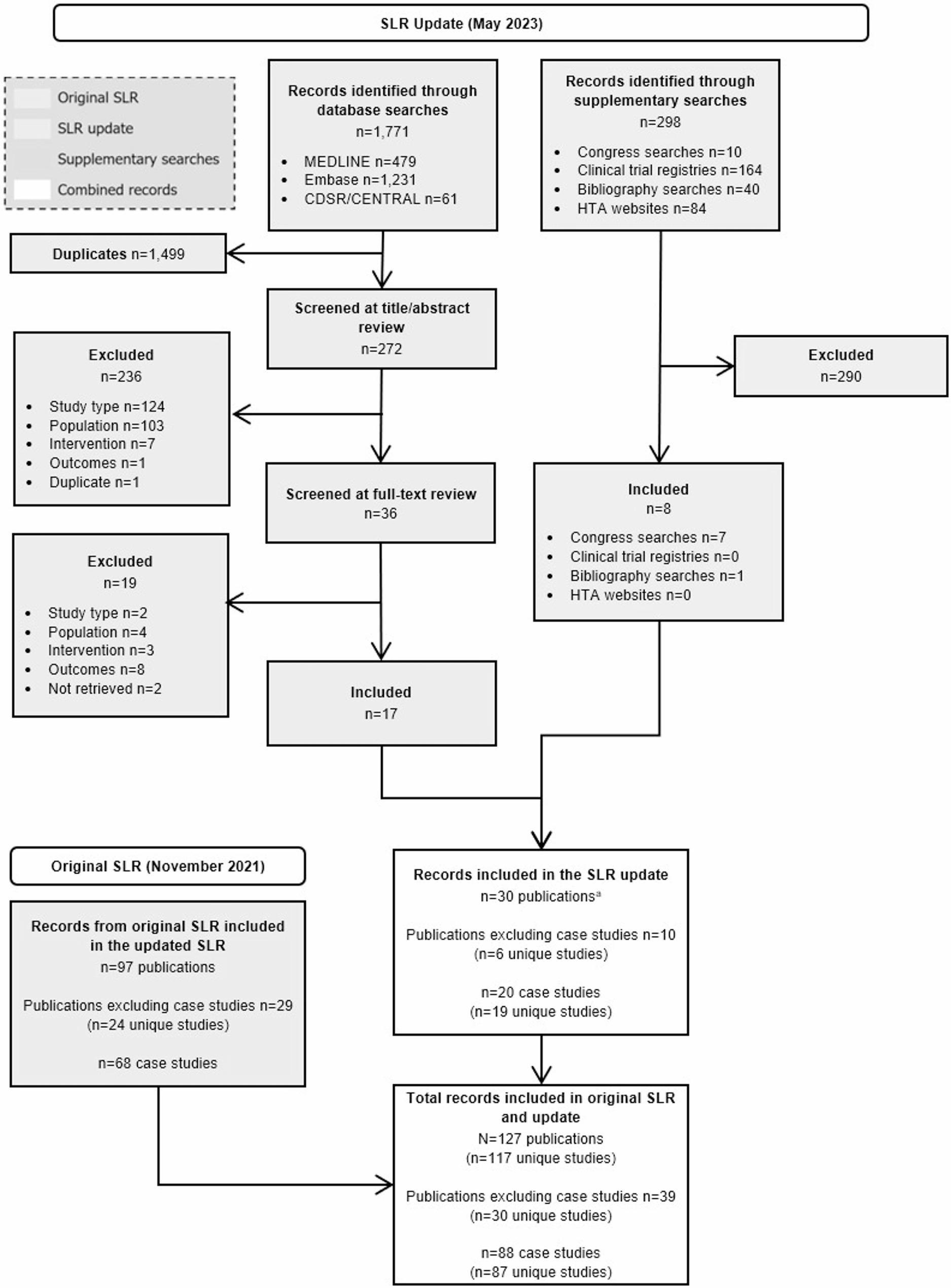
Footnote: ^a^Five publications from original SLR were retrieved during the SLR update. Abbreviations: CDSR: Cochrane Database of Systematic Reviews; CENTRAL: Cochrane Central Register of Controlled Trials; Embase: Excerpta Medical Database; HTA: health technology assessment; MEDLINE: Medical Literature Analysis and Retrieval System Online; PRISMA: Preferred Reporting Items for Systematic reviews and Meta-Analyses; SLR: systematic literature review

**Fig. 2:**
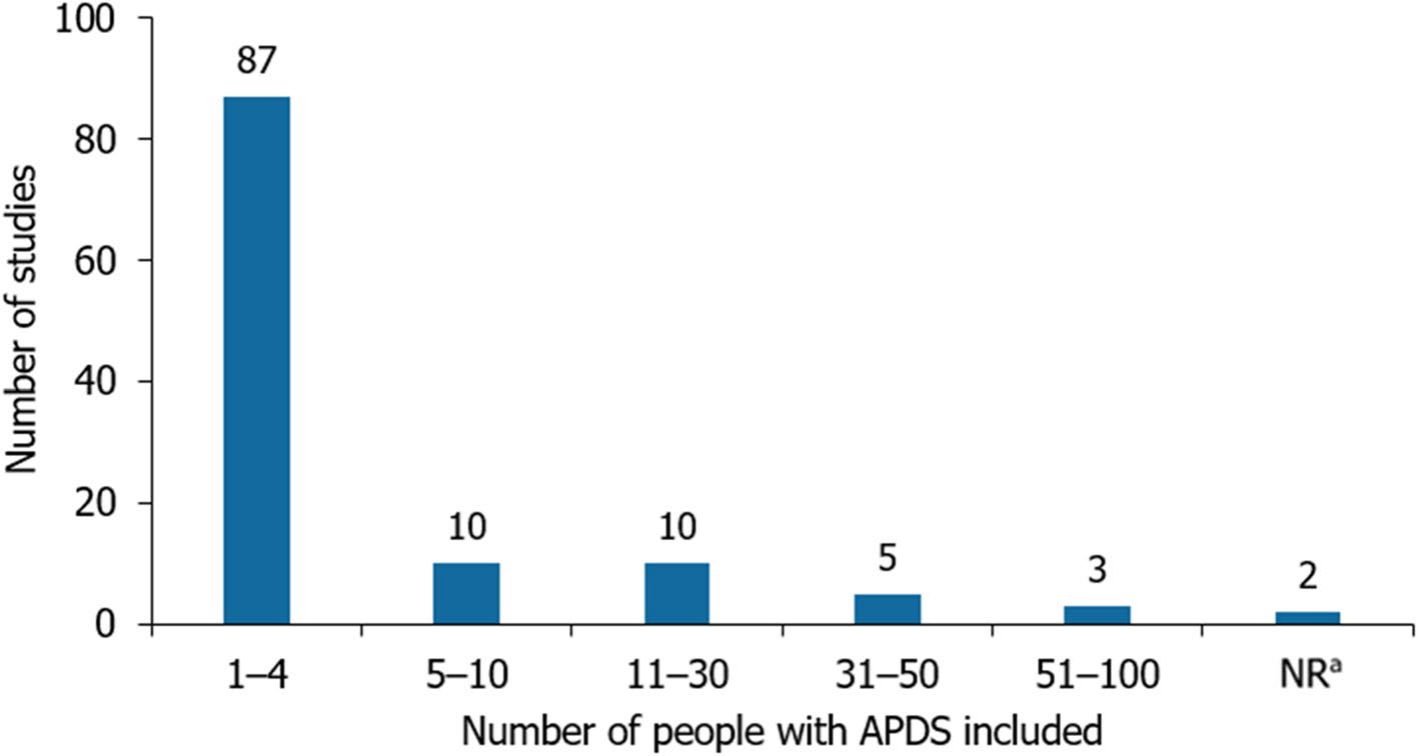
Sample size of included studies in the clinical SLR (*n*= 117). Footnote: An additional publication with a sample size of 170 was published after the search date [152]. Only participants with a reported APDS diagnosis are included in the sample size. Includes both studies and case reports. ^a^Sample size was not reported for *n*= 2 studies. Abbreviations: APDS: activated phosphoinositide 3-kinase delta syndrome; NR: not reported; systematic literature review

**Fig. 3:**
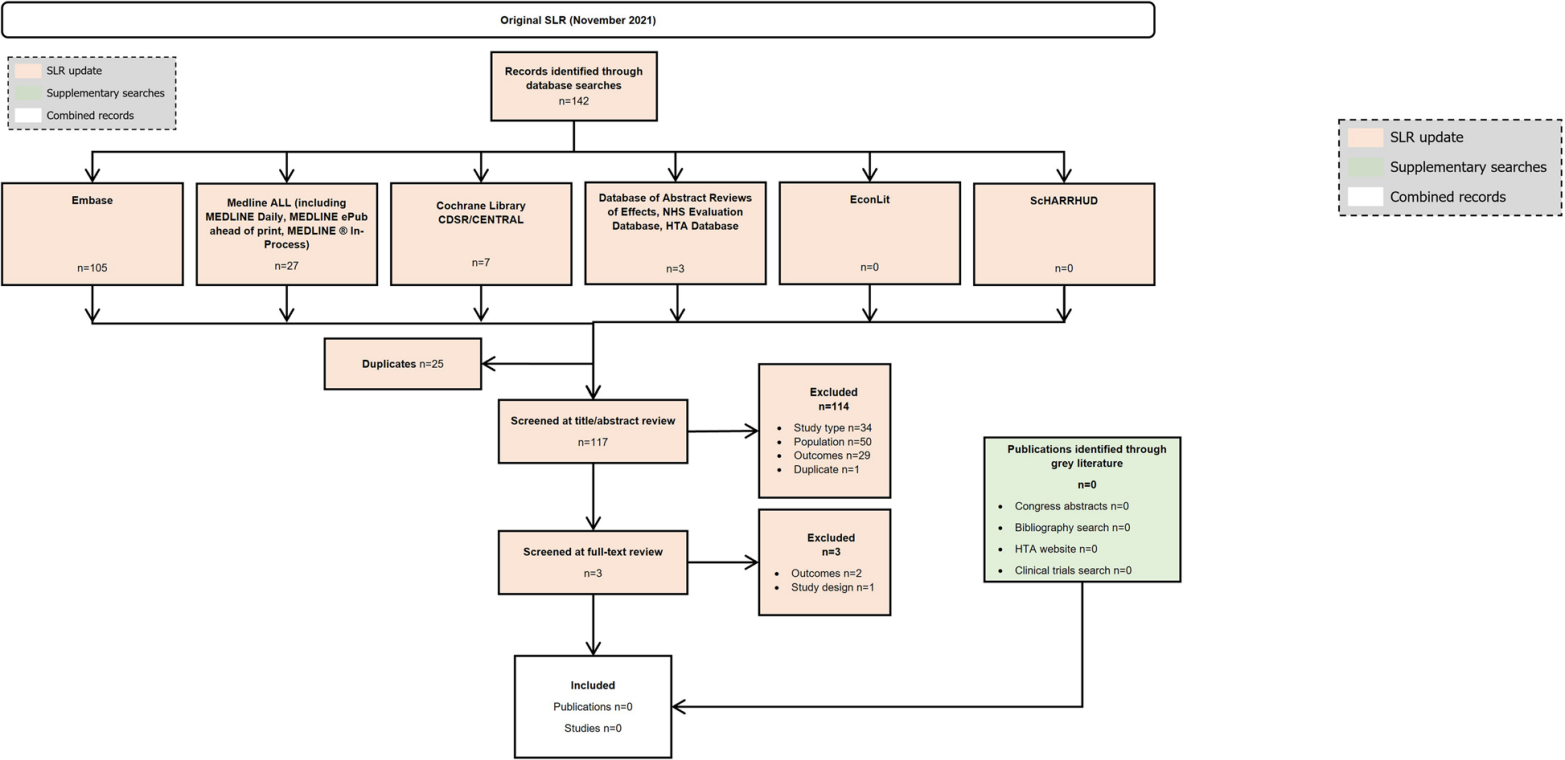
PRISMA diagram of records identified in the epidemiology SLR. Abbreviations: CDSR: Cochrane Database of Systematic Reviews; CENTRAL: Cochrane Central Register of Controlled Trials; EconLit: Economics Literature; Embase: Excerpta Medical Database; HTA: health technology assessment; MEDLINE: Medical Literature Analysis and Retrieval System Online; NHS: National Health Service; PRISMA: Preferred Reporting Items for Systematic reviews and Meta-Analyses; ScHARRHUD: School of Health and Related Research Health Utilities Database; SLR: systematic literature review

**Fig. 4:**
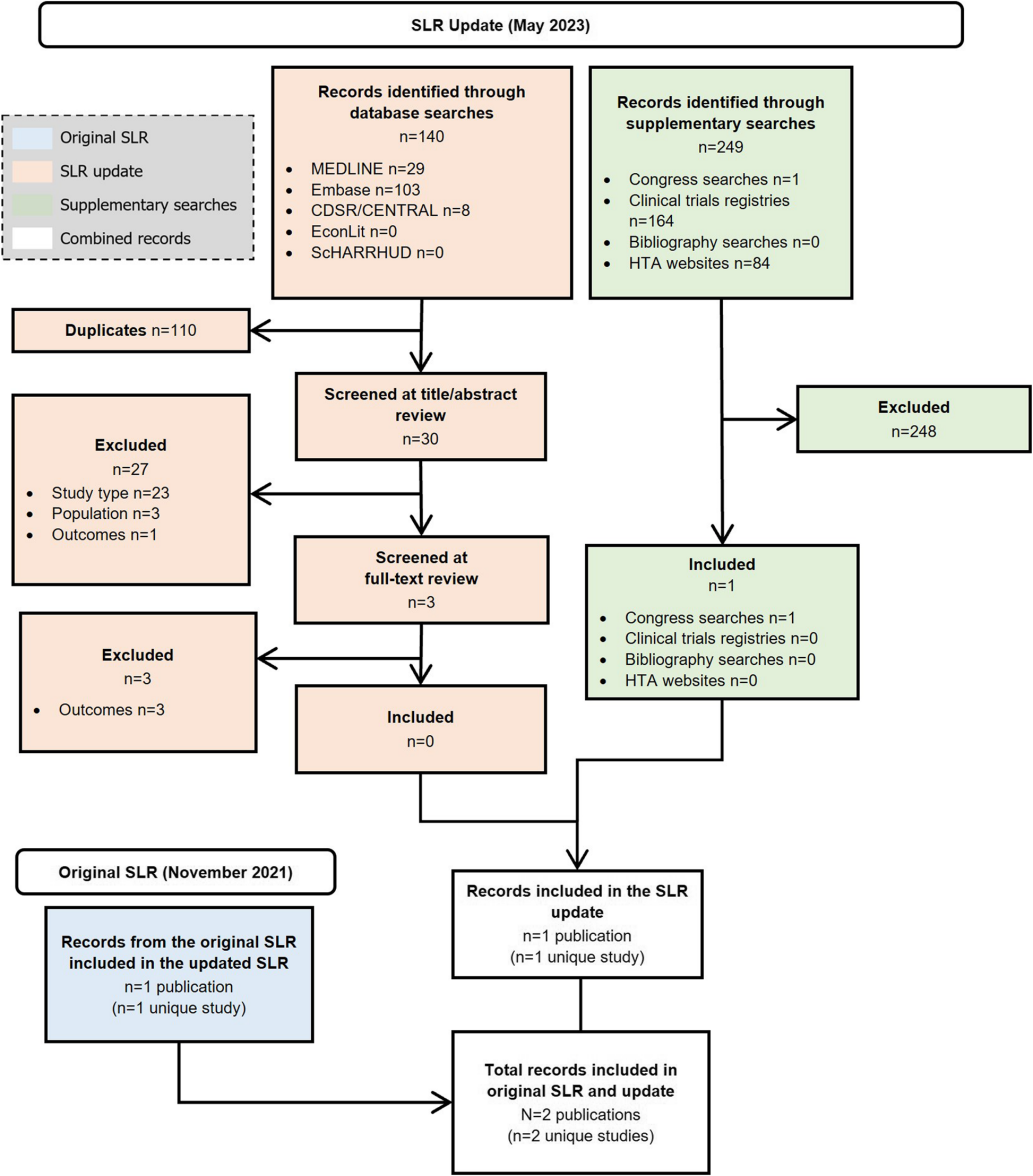
PRISMA diagram of records identified in the economic SLR. Abbreviations: CDSR: Cochrane Database of Systematic Reviews; CENTRAL: Cochrane Central Register of Controlled Trials; Embase: Excerpta Medical Database; HTA: health technology assessment; MEDLINE: Medical Literature Analysis and Retrieval System Online; PRISMA: Preferred Reporting Items for Systematic reviews and Meta-Analyses; ScHARRHUD: School of Health and Related Research Health Utilities Database; SLR: systematic literature review

**Fig. 5:**
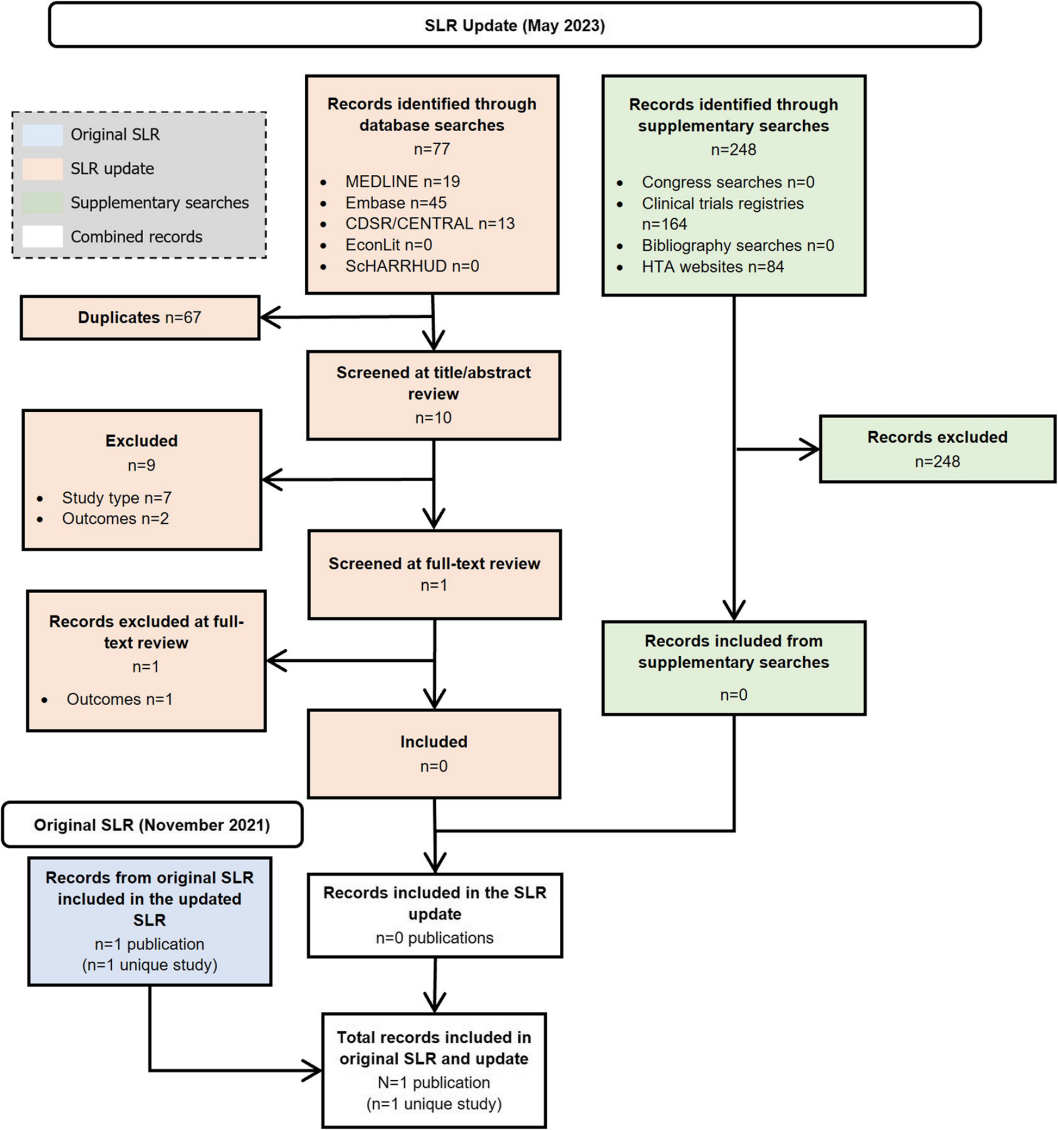
PRISMA diagram of records identified in the HRQoL SLR. Abbreviations: CDSR: Cochrane Database of Systematic Reviews; CENTRAL: Cochrane Central Register of Controlled Trials; Embase: Excerpta Medical Database; HRQoL: health-related quality of life; HTA: health technology assessment; MEDLINE: Medical Literature Analysis and Retrieval System Online; PRISMA: Preferred Reporting Items for Systematic reviews; ScHARRHUD: School of Health and Related Research Health Utilities Database

In the clinical SLR, (Fig. 1), 30/117 (25.6%) were unique clinical studies reporting on ≥5 patients [3, 7, 9, 11, 16, 22, 119–150], with the remaining studies identified (*n*= 87/117, 74.4%) reporting on <5 patients (defined henceforth as case reports; Fig. 2) [31–118]. No relevant records were found in the epidemiology SLR (Fig. 3). In the economic SLR, two unique studies were included (Fig. 4) [33, 151]. Only one unique study was identified in the HRQoL SLR (Fig. 5) [9].

### Clinical SLR

#### Patient characteristics

In the clinical SLR, 30 unique studies reported on ≥5 participants [3, 7, 9, 11, 16, 22, 119–149]. Despite APDS being a progressive condition, making age an important factor in assessing the effectiveness of treatments, some studies (*n*= 5/30, 16.7%) did not report the age of the patient population [3, 122–124, 129, 130, 133, 135, 145]. For studies reporting age as a range (*n*= 15/30, 50.0%), individuals ranged from <1–66 years of age at the time of the study [7, 9, 11, 16, 22, 119, 121, 125, 127, 128, 134, 136, 137, 139, 140, 143, 144, 146, 150, 153]. Furthermore, mean age at the time of study ranged from 17.1 to 36.6 years (*n*= 5/25, 20.0%) [7, 11, 119, 127, 143, 144], and median age at the time of study ranged from 8 to 22 years (*n*= 8/25, 32.0%) [16, 22, 121, 125, 128, 134, 136, 137, 139, 140, 152–154].

Sex is a commonly reported baseline characteristic for clinical studies, however only *n*= 22/30 (73.3%) studies here reported on the sex of participants [3, 7, 9, 16, 22, 120–122, 125–129, 131, 132, 135–144, 146–150]. Across studies which reported on sex, there were 408 participants in total: 155 were female (38.0%) and 253 were male (62.0%).

Of the studies reporting on APDS type (*n*=26/30, 86.7%) [3, 7, 9, 11, 16, 22, 119, 121–123, 125, 127–132, 134–145, 147–150], *n*= 15/26 (57.7%) reported on patients with APDS1 only (including when APDS type was inferred based on the specific gene mutation) [3, 7, 119, 122, 127, 130–132, 135, 137, 139, 141–145, 147–149], *n*= 2/26 (7.7%) reported on patients with APDS2 only [16, 129], and *n*=9/26 (34.6%) reported on a mixed population of individuals with APDS1 and APDS2 [9, 11, 22, 121, 123, 125, 128, 134, 136, 138, 140, 150]. Two studies reported survival analysis for patients with a particular APDS type: one study focused on 23 individuals with APDS1, and the other on 36 individuals with APDS2. Both studies estimated a 30-year survival rate of 83% [16, 135].

#### Study follow-up duration

Approximately half of the unique studies reporting on ≥5 individuals in the clinical SLR reported a follow-up duration (*n*= 14/30, 46.7%), using a mixture of metrics [7, 9, 22, 120, 122, 126, 128, 129, 134, 136–140, 143–145, 148, 150]. Among these studies, 35.7% (*n*= 5/14) reported follow-up duration as a median, ranging from 21 months to four years [128, 134, 136, 143, 144, 150], while 50.0% (*n*= 7/14) reported it as a range, spanning from one month to 21.7 years [7, 128, 134, 136, 143–145, 148]. For HSCT, the only potentially curative treatment, the median follow-up duration varied from 27 months to four years (*n*= 2/5, 40.0% of studies reporting on median follow-up duration reported on HSCT) [134, 150] and the overall range for follow-up was four months to 18 years (*n*= 2/7, 28.6% of studies reporting range of follow-up reported on HSCT) [7, 134].

#### Types of interventions

**Fig. 6:**
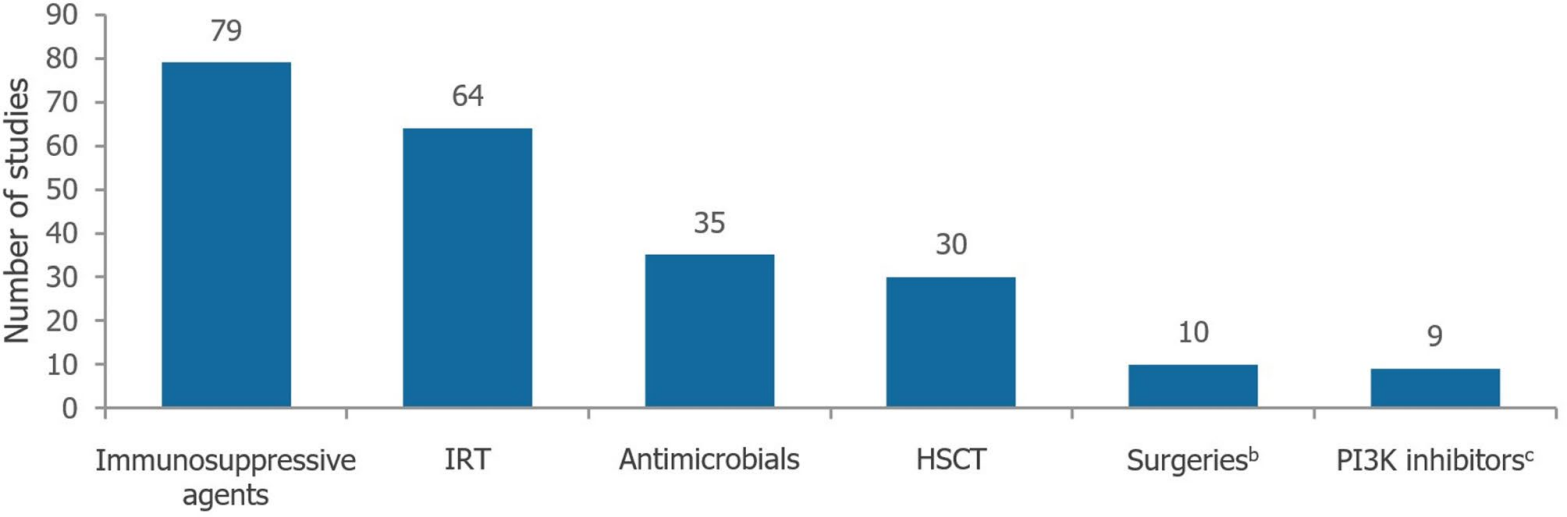
Count of interventions across included studies in the clinical SLR (*n*= 117)^a^. Footnotes: ^a^117 unique studies were included in the clinical SLR, with a total of 227 interventions across all studies, irrespective of the number of people with APDS. ^b^Surgeries include splenectomy, adenotonsillectomy, bowel resection and hemicolectomy. ^c^PI3 K inhibitors include leniolisib, nemiralisib and seletalisib. Abbreviations: APDS: activated phosphoinositide 3-kinase delta syndrome; HSCT: haematopoietic stem cell transplantation; IRT: immunoglobulin replacement therapy; PI3 K: phosphoinositide 3-kinase delta; SLR: systematic literature review

Clinical studies and case reports on interventions of interest for APDS were documented. Immunosuppressive agents were the most reported intervention (*n*= 79/117, 67.5%) [7, 11, 16, 32–34, 36, 37, 40–42, 44, 46, 48, 49, 51, 52, 56, 57, 59–62, 64–69, 71–78, 81–84, 87–91, 95–106, 108–112, 114–118, 121, 122, 127, 131, 132, 136, 141–146, 148, 149], including but not limited to: corticosteroids, mTOR inhibitors (including sirolimus/rapamycin and everolimus) and rituximab. These treatments were grouped for analysis due to the limited number of studies reporting outcomes for each specific immunosuppressive agent. The next most reported interventions were IRT (*n*= 64/117, 54.7%) [3, 7, 11, 31, 33–36, 38, 40–47, 49, 50, 52–54, 57, 59–62, 66, 67, 69–73, 75, 76, 79, 80, 82, 83, 85–90, 92, 93, 96, 98, 99, 101, 102, 108–112, 114, 121, 122, 127, 128, 136, 148], antimicrobials (*n*= 34/117, 29.1%) [3, 11, 31, 36, 43, 55, 58–61, 65, 67, 70, 71, 76, 79, 83, 84, 86–89, 100, 101, 103, 105, 108, 109, 111, 114, 115, 121, 127, 128] and HSCT (*n*= 31/117, 26.5%) [3, 7, 11, 16, 39, 41, 50, 63, 64, 72, 78, 94, 96, 97, 102, 106–108, 111, 113, 114, 117, 119, 123, 126, 129, 130, 134–136, 147, 150], as shown in Fig. 6. The majority of included records (*n*= 76/117, 65.0%) reported outcomes in individuals receiving multiple interventions/intervention types.

#### Treatment effectiveness

In the studies that reported on the effectiveness of interventions, current symptomatic treatments for APDS were only partially effective at controlling manifestations. Results by intervention type were as follows.

##### IRT

Only one of the studies reporting on IRT (*n*= 1/9, 11.1%) recorded improvements in immune dysregulation; lymphoproliferation regressed in *n*= 4/6 (66.7%) participants receiving IRT together with antimicrobial prophylaxis, and in *n*= 1/3 (33.3%) participants receiving IRT alone [121]. On the other hand, most IRT studies reported improvements in immune deficiency with treatment (*n*= 6/7, 85.7% of IRT studies describing immune deficiency) [7, 121, 122, 127, 136, 148].

##### Immunosuppressive treatment

Outcomes of immunosuppressive treatments varied in the 14 studies found with reports of normalised immunophenotypes (*n*=7/14, 50.0%) [121, 131, 132, 136, 141, 142, 145, 146, 149], reduced lymphoproliferation (*n*= 10/14, 71.4%) [7, 11, 16, 121, 127, 136, 143–145, 148, 149] and reduced infections (*n*= 5/14, 35.7%) [16, 121, 143–145, 149].

##### HSCT

HSCT showed high donor engraftment (>90%) in the majority of participants in most studies (*n*= 9/13, 69.2%) [3, 7, 16, 123, 126, 130, 134, 135, 147, 150], with a correlation reported between graft failure and mTOR inhibitor use in the first year post-HCST in one study [126, 150]. However, immunophenotype changes were only reported in two studies (*n*= 2/13, 15.4%) and three case reports (*n*=3/18, 16.7%), in all of which normalisation was observed [50, 94, 108, 119, 147]. 

##### Surgery

Surgeries, such as splenectomy, tonsillectomy, bowel resection, adenotonsillectomy hemicolectomy and laparotomy, were reported in 10 unique studies/case reports in individuals with APDS (*n*= 10/117, 8.5%), with varying effectiveness [3, 7, 11, 32, 48, 53, 68, 105, 130, 135]. In one study reporting on individuals who underwent tonsillectomy (*n*= 25/68, 36.8%), a clear benefit of the intervention was observed in only 7 of the 25 participants (28.0%) [11]. Poor outcomes were reported for individuals who underwent splenectomies in most studies that reported outcomes following a splenectomy (*n*= 2/3, 66.7%) [3, 7]. One case report stated that adenotonsillectomy, in addition to treatment with antibiotics, salbutamol and corticosteroids, was unable to relieve symptoms of dyspnoea and recurrent respiratory tract infections [105]. 

##### Antimicrobials

A total of 34 unique studies/case reports (*n*= 34/117, 29.1%) reported the use of antimicrobials, including antibiotics, antivirals and antifungals [3, 11, 31, 36, 43, 55, 58–61, 65, 67, 70, 71, 76, 79, 83, 84, 86–89, 100, 101, 103, 105, 108, 109, 111, 114, 115, 121, 127, 128]. In all three studies reporting on efficacy following antimicrobial use, participants continued to experience symptoms and manifestations of APDS [3, 127, 128]. Antivirals resolved viral infections in one study (*n*= 1/1; 100%) and four case reports (*n*= 4/7; 57.1%), with improvements in symptoms such as lymphadenopathy, hepatosplenomegaly and respiratory infections [60, 100, 111, 114, 121]. Furthermore, remission of fungal infection was reported in the sole participant in the only study reporting efficacy outcomes following antifungal use [121].

##### PI3 K inhibitors

There were nine unique studies/case reports which reported on PI3 K inhibitors (*n*= 9/117, 7.7%), including leniolisib (*n*= 7/117, 6.0%), nemiralisib (*n*= 1/117, 0.9%) and seletalisib (*n*= 2/117, 1.7%) [9, 22, 88, 91, 120, 121, 124, 125, 139, 146]. No meaningful changes in phosphatidylinositol (3,4,5)-trisphosphate (PIP3; the enzyme product of PI3 Kδ), inflammatory markers or circulating B and T cell subpopulations were reported in the single study of nemiralisib [120]. In the sole seletalisib study reporting on immune improvements, a reduction in transitional B cells, an increase in naïve B cell levels and long-term improvements in CD8^+^ T cell levels were observed [125]. Three leniolisib studies/case reports (*n*= 3/7, 42.9%) reported on immune dysregulation, including a two-part, 12-week clinical trial with an open-label dose escalation part and a Phase III randomised controlled trial (RCT) part, and an open-label extension (OLE) study. All these studies found that leniolisib improved immune dysregulation, including reductions in lymphadenopathy and spleen volume, and improved the immunologic phenotype, with discontinuation of IRT in some patients [9, 22, 91, 139]. 

#### Safety and tolerability

Tolerability of interventions for APDS was variable, in both the clinical studies and case reports.

##### IRT

In the European Society for Immunodeficiencies (ESID) registry, IRT was reported to be generally well-tolerated [11], but its tolerability remains unclear as only 20.3% (*n*= 13/64) of IRT studies reported safety outcomes related to IRT [7, 11, 36, 60, 62, 67, 69, 73, 109, 110, 112, 114, 136]. Of the 10 case reports mentioning IRT use, three (30.0%) reported AEs related to IRT and five (50.0%) reported AEs when receiving IRT alongside concomitant treatment(s) [36, 60, 62, 67, 73, 109, 110, 112, 114].

##### Immunosuppressive treatment

Immunosuppressive treatments were poorly tolerated and/or resulted in AEs in the majority of studies (*n*= 4/5, 80.0%) and case reports (*n*= 12/15, 80.0%) [7, 59, 60, 62, 64, 82, 83, 88, 91, 99, 112, 114, 115, 127, 136, 149]; AEs resulted in discontinuation in three studies (*n*= 3/5, 60.0%) and seven case reports (*n*= 7/15, 46.7%) [7, 59, 60, 69, 82, 88, 91, 99, 127, 149]. AEs reported following immunosuppressive treatment were varied, and included renal toxicity, recurrent aphthous stomatitis, oral ulceration, recurrent headaches, colitis, poor appetite, weight loss, abdominal pain, hypertension, neutropenia, continuous and severe infectious episodes, pneumonia and abrupt rising of serum creatinine and sirolimus trough levels (in response to an increased dose) [7, 59, 60, 62, 64, 82, 83, 88, 91, 99, 112, 114, 115, 127, 136, 149]. 

##### HSCT

Safety outcomes following HSCT were reported in the majority of studies/case reports (*n*= 21/31, 67.6%), including presence of graft versus host disease (GVHD), AEs and/or complications, such as organ toxicities, infections and non-infectious complications [7, 16, 39, 50, 64, 78, 94, 97, 106, 108, 111, 113, 114, 117, 129, 130, 134–136, 150, 152]. Of these studies/case reports, five (*n*= 5/21, 23.8%) reported pulmonary and/or renal complications [7, 11, 106, 136, 150]. One study reported adverse complications in 90.9% of individuals (*n*= 10/11) [135], and another study reported HSCT-related mortality in 14.0% (*n*= 8/57) of people with APDS treated with HSCT [150]. In a retrospective case series that characterised HSCT outcomes in individuals with APDS (median follow-up: 27 months), acute GVHD was reported in *n*= 22/57 (38.6%) participants, and chronic GVHD was reported in *n*= 9/57 (15.8%) participants [126, 150].

##### Surgery

In the studies reporting on surgery, four reported safety outcomes (*n*=4/10, 40.0%) [7, 53, 130, 135]. Three of these studies (*n*= 3/4, 75.0%) reported death from sepsis following the combined use of splenectomy and HSCT [7, 130, 135]. 

##### Antimicrobials

Three case reports reported safety outcomes related to antimicrobials (*n*= 3/34, 8.8% of all studies/case reports reporting the use of antimicrobials) [60, 87, 89]. In one case report, antibiotics (piperacillin/tazobactam) were reported to lead to possible ‘drug fever’, requiring antibiotic escalation and ventilation alongside prednisolone treatment [89]. In the other two case reports, antivirals (remdesivir, nitazoxanide and valganciclovir) were reported to be well-tolerated [60, 87].

##### PI3 K inhibitors

Safety outcomes were reported for *n*= 6/9 (66.7%) studies/case reports discussing the use of PI3 K inhibitors [9, 22, 88, 91, 120, 125, 139]. Nemiralisib was reported to be well-tolerated in an interventional study, in which reported AEs were mostly respiratory in nature, and no deaths or severe AEs were reported [120]. Seletalisib showed acceptable tolerability in the majority of participants during a Phase Ib study and its OLE: seletalisib-related AEs were reported in *n*= 4/7 (57.1%) patients, with *n*= 2/7 (28.6%) patients prematurely discontinuing seletalisib because of potential drug-induced liver injury AEs (one of these patients had a history of elevated liver enzyme) [125]. In all four leniolisib studies (two interventional and two case reports) reporting safety outcomes, no treatment-related serious AEs were reported [9, 22, 88, 91, 139]. In the RCT, leniolisib demonstrated a tolerability profile comparable to that of the placebo, with 85.7% of participants (*n*= 18/21) treated with leniolisib reporting AEs vs. 90.0% of participants (*n*=9/10) in the placebo arm, and a lower incidence of study drug-related AEs (23.8% [*n*= 5/21] for leniolisib vs. 30.0% [*n*= 3/10] for placebo) [22]. In the five-year OLE of the RCT, Grade 1–3 AEs were reported in 86.5% of participants (*n*= 32/37), no Grade 4 AEs were reported, there was one Grade 5 AE (*n*= 1/37, 2.7%), and leniolisib-related AEs were reported in 13.5% of participants (*n*= 5/37) [139].

### Epidemiology SLR

No studies reporting on APDS epidemiology were identified in the epidemiology SLR (Fig. 3).

### Economic SLR

In the economic SLR, two studies were identified that showed that the direct cost of APDS was high (Fig. 4) [33, 151]. A US study used a conceptual model to summarise APDS-associated manifestations and medication costs based on a survey of experts treating people with APDS. The total estimated average annual medical cost among people with APDS was 83,057–793,620 US Dollars (USD) for the year 2022 [151]. Infections, haematologic pathology and malignancy were the greatest drivers of manifestation costs, with IRT and HSCT being the greatest contributors to annual treatment cost. The full extraction table for the economic SLR is presented in the Supplementary Results 2: Economic SLR.

### HRQoL SLR

In the HRQoL SLR, only one unique study was identified (Fig. 5) [9]. This dose-escalation study, which was the first part of the two-part leniolisib clinical trial, aimed to identify the optimal dose of leniolisib by gradually increasing the dosage until achieving the desired effect or encountering unacceptable side effects in *n*= 6 individuals [9]. The study utilised the Patient Global Assessment (PtGA), measured using a visual analogue scale (VAS) represented by a 100 mm line [9]. The study found that 12 weeks of leniolisib treatment improved patient well-being by a mean of 11 mm (standard deviation: ±11 mm; range: −3 to +22 mm) [9]. Textual patient narratives were also provided by the investigator, and within these, increased energy levels and/or decreased fatigue were described in all six patients following treatment [9]. The full extraction table for the HRQoL SLR is presented in the Supplementary Results 3: HRQoL SLR.

## Discussion

Four broad and methodologically robust SLRs were conducted and identified a clear paucity of evidence on clinical outcomes, epidemiology, HRQoL and costs in APDS. No evidence was identified related to epidemiology, cost-effectiveness of treatments or utilities in APDS; only one study on HRQoL and two studies on costs/HCRU were identified. Furthermore, the type and frequency of outcomes reported by studies in the clinical SLR were highly variable. Given the ultra-rare nature of APDS and its recent recognition [4], the lack of evidence is expected. This emphasises the need for enhanced data collection and analysis from individuals with APDS, with a more comprehensive and less heterogeneous approach, to gain a more comprehensive understanding of the disease burden outside of clinical studies.

The clinical SLR highlighted that there remains a considerable clinical burden of APDS, due to the effects on multiple organ systems and the lack of widely available, effective treatments. The current clinical landscape includes symptomatic treatments with limited effectiveness [8, 15, 17], HSCT, which is potentially curative but is often performed after a number of complications have arisen, and is associated with serious morbidity and mortality [3, 11, 15, 150], and leniolisib, which addresses the underlying cause but is currently licensed only in the US, the UK and Israel [9, 16–24]. Therefore, there remains a significant need for a widely available pharmacological treatment that targets the underlying cause of APDS.

Of the treatments that were studied in the clinical SLR, there was a discrepancy between the expected and actual frequencies of reported interventions. For instance, less than a third of studies mentioned patients receiving antimicrobials, despite infections being almost universal in individuals with APDS as per Maccari et al. 2023 [152]. Furthermore, approximately one quarter of studies focused on HSCT, despite only 12.8% of individuals with APDS (*N*= 243) being reported to have received this treatment, according to a literature review of 55 articles on individuals with APDS [10]. This imbalance in reporting rates versus expected treatment use rates may be indicative of publication bias and/or reporting bias; for example, antimicrobials may potentially be deemed less noteworthy because they are perceived as a standard treatment. Finally, PI3 Kδ inhibitors were the least frequently reported interventions, reflecting the recent development of these novel and promising treatments [9, 22, 88, 91, 121, 124, 137, 139, 140, 146, 153, 154].

While the original epidemiology SLR returned no articles and an updated epidemiology SLR was not performed, a recent publication by Vanselow et al. estimates that 1–2 individuals per million live with APDS [155]. It is expected that APDS is underdiagnosed due to its recent recognition, lack of awareness of the condition and the diagnostic difficulties presented by the heterogeneous manifestations [4, 50, 155]. Therefore, prevalence estimates of APDS may increase as awareness of the condition improves and evidence to characterise the disease continues to accumulate [4, 50, 156].

The clinical SLR revealed inconsistencies and variability in the reporting of study design, participant characteristics, patient compliance and outcomes. Less than half (*n*= 12/30, 40.0%) of the extracted studieswith ≥5 patients clearly described the intervention(s) of interest, such as information on dose and route of administration. Additionally, compliance with the interventions was poorly reported (not reported, unable to determine or not applicable in 80.0% of studies [*n*= 24/30]), precluding the inference of any causal relationship between interventions and outcomes. Moreover, since studies typically report outcomes for individuals who receive multiple treatments, it is not possible to attribute any one outcome to a specific treatment. Consequently, the evidence on treatment effectiveness remains minimal, with findings indicating that symptomatic treatment options are ineffective at managing all symptoms related to APDS. Furthermore, most studies were non-randomised, observational, short in duration and/or reported across multiple interventions, thereby limiting the interpretation of the efficacy and safety outcomes. Statistical analyses in the included studies were limited, with blinding and randomisation only reported for one study, the aforementioned triple-blinded Phase III RCT of leniolisib vs. placebo (*n*= 1/120, 0.8% of studies) [22]. Notably, this RCT was sufficiently powered to detect a clinically important effect [22]. In contrast, no formal power analysis was reported for any of the remaining extracted studies, due to the small sample size and study design. This underscores the importance of establishing a consensus on how clinical outcomes for APDS should be reported and presents a clear area for future study. Achieving standardised reporting will facilitate more straightforward comparison of studies and the treatments they investigate.

Further characterisation of APDS and its treatments is required, and since these SLRs were conducted, additional data have been published [157–163]. Two recent studies examined mortality in a mixed cohort of APDS1 and APDS2 patients. One study reported a 74% probability of 30-year survival based on a cohort of 256 individuals [164]. Another study, with a cohort of 33 individuals, found survival probabilities of 75.8% by age 30, 70.4% by age 40, and 66.7% by age 65 [165]. Both studies showed lower survival probabilities compared to a previously reported 30-year survival rate of 83%, highlighting the unmet need for targeted treatments to potentially increase survival in this population [16, 135]. Recent US HCRU data in APDS have shown that outpatient visits are the most commonly used healthcare resources, primarily due to respiratory diseases and immunodeficiency [163]. This aligns with the two studies identified in the economic SLR, showing the high direct costs associated with APDS [33, 151]. Furthermore, additional studies have demonstrated the long-term efficacy and safety of leniolisib, as well as the HRQoL benefits, highlighting its potential benefit for APDS patients. Notably, analysis of six individuals with APDS from the leniolisib open-label dose escalation study revealed clinically meaningful improvements as measured by the Short Form Health Survey 36 (SF-36), with the mean physical component score increasing by 4.0 points and social functioning by 7.9 points at year 5 [166]. In addition, a recent qualitative study analysing patient experiences of APDS and leniolisib treatment found that leniolisib improved symptoms and HRQoL, with 21/36 included individuals (58.3%) attributing ≥1 improvement in symptoms and 27/36 (75.0%) attributing ≥1 improvement in HRQoL to leniolisib [167]. These comprehensive data on leniolisib, enabled by the carrying out of a Phase III study, allow for valuable conclusions to be drawn that were not possible to investigate with other interventions due to lack of data, either as these treatments have not progressed to Phase III trials in individuals with APDS or due to APDS being an ultra-rare and under-researched condition. Collectively, these studies highlight the high costs associated with APDS while emphasising the HRQoL and clinical benefit of leniolisib, underscoring the significant need to improve the available care for APDS through widely available therapies that address the underlying cause of disease. Overall, these recently published data align with the findings from the SLRs; however, there are still significant gaps in APDS research, particularly regarding costs, HCRU, HRQoL and epidemiology, indicating a need for further research and analysis.

The key strengths of these SLRs were the rigor, thoroughness and breadth of the review processes, to ensure that relevant evidence was not missed. The reviews used systematic methods in line with the Cochrane Handbook for Systematic Reviews of Interventions to conduct exhaustive searches of the literature on multiple electronic databases, identifying evidence relevant to the review objectives [168]. Additionally, supplementary searches were conducted to ensure that any relevant studies that were not identified by the database searches were not missed. Conference proceedings from 2019 onwards were hand-searched to identify relevant, high quality conference research that had not yet been published as a full manuscript, minimising the impact of publication bias in the results of the SLRs. The inclusion of observational study designs in the clinical SLR was valuable, as real-world data provided the majority of identified evidence, particularly for long-term treatment outcomes. Finally, there were no restrictions on language, geography or date of publication in the inclusion criteria, ensuring all potentially relevant articles were screened.

There were some methodological limitations that should be considered when interpreting the results of this SLR. Studies which had full texts that could not be translated into English were assessed for eligibility and extracted based on their English abstracts alone; this may have resulted in the omission of relevant data which were not included in the abstracts. Additionally, interpretation of data was conducted via qualitative analyses, with no formal statistical synthesis or meta-analysis performed due to the heterogeneity of data reporting. The generalisability of the results drawn from the included studies is uncertain. Finally, the broad scope of these SLRs may have limited the potential for detailed insights into specific topics to be drawn.

## Conclusion

These methodologically robust SLRs found that outcome reporting in most studies of patients with APDS was heterogeneous and inconsistent, sample sizes were small and patients often received multiple treatments, thereby limiting the available evidence and interpretation of results. Furthermore, current clinical treatments primarily manage individual symptoms (except for HSCT which, while potentially curative, carries significant morbidity and mortality risks), with leniolisib being the only comprehensively studied and licensed treatment option that targets the underlying pathology. Our findings highlight the need for more rigorous data collection to evaluate the clinical and economic effectiveness of APDS treatments.

## Supplementary Information

Supplementary Material 1.

## Data Availability

The datasets used and/or analysed during the current study are available from the corresponding author on reasonable request.
