# Supplementary material for: Systematic literature reviews to identify epidemiological, clinical, economic and health-related quality of life evidence in activated PI3Kδ syndrome (APDS)
Source: BMC Immunol. 2025 Jul 19;26:52. doi: 10.1186/s12865-025-00723-6 (PMC12275335; doi:10.1186/s12865-025-00723-6)
Supplement: Supplementary file 1 — Supplementary Material 1. [file 12865_2025_723_MOESM1_ESM.docx]

# SUPPLEMENTARY MATERIALS

Supplementary Methods 1: Search Terms

Search Terms for Electronic Databases

*Clinical SLR*

**Supplementary Table 1**: MEDLINE^®^ search strategy for the original clinical SLR (via Ovid.com)

| **#** | **Search Term** | **Hits**  **11/11/2021** |
| --- | --- | --- |
| **1** | ((Activated Phosph* adj8 (Delta* or syndrome*)) or (Activated PI3* adj5 delta*) or (PI3* adj5 syndrome*)).mp. | 206 |
| **2** | (Immunodeficiency 14 or immunodeficiency 36).mp. | 5 |
| **3** | ((P110* adj8 ((mutation and immunodeficien* and lymphadenopathy) or syndrome*)) or PASLI* or (mutat* adj5 (PIK3CD or PIK3R1)) or (Type* adj2 APDS) or APDS 1 or APDS1 or APDS 2 or APDS2 or IMD14A).mp. | 254 |
| **4** | exp Phosphatidylinositol 3-Kinases/ and (autosomal dominant disorder/ or primary immunodeficiency diseases/) | 68 |
| **5** | or/1-4 | 397 |

**Abbreviations**: MEDLINE: Medical Literature Analysis and Retrieval System Online; SLR: systematic literature review.

**Supplementary Table 2**: MEDLINE^®^ search strategy for the clinical SLR update (via Ovid.com)

| # | Search Term | Hits  18/05/2023 |
| --- | --- | --- |
| **1** | ((Activated Phosph* adj8 (Delta* or syndrome*)) or (Activated PI3* adj5 delta*) or (Activated PI 3* adj5 delta*) or (PI3* adj5 syndrome*)).ti,ab,kf. | 245 |
| **2** | (Immunodeficiency 14 or immunodeficiency 36 or IMD14A or IMD36).ti,ab,kf. | 5 |
| **3** | (P110* adj8 ((delta and activat* and mutation) or syndrome* or immunodeficien*)).ti,ab,kf. | 32 |
| **4** | (PASLI* or (mutat* adj5 (PIK3CD or PIK3R1)) or (Type* adj2 APDS) or (APDS and immunodeficien*) or APDS 1 or APDS1 or APDS 2 or APDS2).ti,ab,kf. | 314 |
| **5** | exp Phosphatidylinositol 3-Kinases/ and (primary immunodeficiency diseases/) | 87 |
| **6** | or/1-5 | 479 |

**Abbreviations**: MEDLINE: Medical Literature Analysis and Retrieval System Online; SLR: systematic literature review.

**Supplementary Table 3**: Embase^®^ search strategy for the original clinical SLR (via Ovid.com)

| # | Search Term | Hits  11/11/2021 |
| --- | --- | --- |
| **1** | ((Activated Phosph* adj8 (Delta* or syndrome*)) or (Activated PI3* adj5 delta*) or (PI3* adj5 syndrome*)).mp. | 325 |
| **2** | (Immunodeficiency 14 or immunodeficiency 36).mp. | 6 |
| **3** | ((P110* adj8 ((mutation and immunodeficien* and lymphadenopathy) or syndrome*)) or PASLI* or (mutat* adj5 (PIK3CD or PIK3R1)) or (Type* adj2 APDS) or APDS 1 or APDS1 or APDS 2 or APDS2 or IMD14A).mp. | 521 |
| **4** | Exp phosphatidylinositol 3 kinase/ and (autosomal dominant disorder/ or immune deficiency/) | 316 |
| **5** | or/1-4 | 980 |

**Abbreviations**: Embase: Excerpta Medica Database; SLR: systematic literature review.

**Supplementary Table 4**: Embase^®^ search strategy for the clinical SLR update (via Ovid.com)

| # | Search Term | Hits  18/05/2023 |
| --- | --- | --- |
| **1** | ((Activated Phosph* adj8 (Delta* or syndrome*)) or (Activated PI3* adj5 delta*) or (Activated PI 3* adj5 delta*) or (PI3* adj5 syndrome*)).ti,ab,kf. | 366 |
| **2** | (Immunodeficiency 14 or immunodeficiency 36 or IMD14A or IMD36).ti,ab,kf. | 7 |
| **3** | (P110* adj8 ((delta and activat* and mutation) or syndrome* or immunodeficien*)).ti,ab,kf. | 60 |
| **4** | (PASLI* or (mutat* adj5 (PIK3CD or PIK3R1)) or (Type* adj2 APDS) or (APDS and immunodeficien*) or APDS 1 or APDS1 or APDS 2 or APDS2).ti,ab,kf. | 644 |
| **5** | exp phosphatidylinositol 3 Kinases/ and (autosomal dominant disorder/ or immune deficiency) | 466 |
| **6** | or/1-5 | 1,231 |

**Abbreviations**: Embase: Excerpta Medica Database; SLR: systematic literature review.

**Supplementary Table 5**: Cochrane Library search strategy for the original clinical SLR (CDSR/CENTRAL, via Cochrane Library interface)

| # | Search Term | Hits  12/11/2021 |
| --- | --- | --- |
| **1** | ((Activated Phosph* NEAR/8 (Delta* or syndrome*)) or (Activated PI3* NEAR/5 delta*) or (PI3* NEAR/5 syndrome*)):ti,ab,kw | 11 |
| **2** | ("Immunodeficiency 14" or "immunodeficiency 36"):ti,ab,kw | 1 |
| **3** | ((P110* NEAR/8 ((mutation and immunodeficien* and lymphadenopathy) or syndrome*)) or PASLI* or (mutat* NEAR/5 (PIK3CD or PIK3R1)) or (Type* NEAR/2 APDS) or APDS 1 or APDS1 or APDS 2 or APDS2 or IMD14A):ti,ab,kw | 40 |
| **4** | [mh "Phosphatidylinositol 3-Kinases"] and ([mh ^"autosomal dominant disorder"] or [mh ^"primary immunodeficiency diseases"]) | 0 |
| **5** | {Or #1-#4} | 50 |

**Abbreviations**: CDSR: Cochrane Database of Systematic Reviews; CENTRAL: Cochrane Central Register of Controlled Trials; SLR: systematic literature review.

**Supplementary Table 6**: Cochrane Library search strategy for the clinical SLR update (CDSR/CENTRAL, via Cochrane Library interface)

| **#** | **Search Term** | **Hits**  **18/05/2023** |
| --- | --- | --- |
| **1** | ((Activated Phosph* NEAR/8 (Delta* or syndrome*)) or (Activated PI3* NEAR/5 delta*) or (Activated PI 3* NEAR/5 delta*) or (PI3* NEAR/5 syndrome*)):ti,ab,kw | 17 |
| **2** | ("Immunodeficiency 14" or "immunodeficiency 36" or IMD14A or IMD36):ti,ab,kw | 1 |
| **3** | (P110* NEAR/8 ((delta and activat* and mutation) or syndrome* or immunodeficien*)):ti,ab,kw | 5 |
| **4** | (PASLI* or (mutat* NEAR/5 (PIK3CD or PIK3R1)) or (Type* NEAR/2 APDS) or (APDS and immunodeficien*) or APDS 1 or APDS1 or APDS 2 or APDS2):ti,ab,kw | 46 |
| **5** | [mh "Phosphatidylinositol 3-Kinases"] and [mh ^"primary immunodeficiency diseases"] | 0 |
| **6** | {Or #1-#5} | 61 |

**Abbreviations**: CDSR: Cochrane Database of Systematic Reviews; CENTRAL: Cochrane Central Register of Controlled Trials; SLR: systematic literature review.

**Supplementary Table 7**: CRD database search strategy for the original clinical SLR (via York.ac.uk/crd)^a^

| **#** | **Search Term** | **Hits**  **11/11/2021** |
| --- | --- | --- |
| **1** | Activated Phosph* or PI3* or Immunodeficiency 14 or immunodeficiency 36 or P110* or PASLI* or PIK3CD or PIK3R1 or APDS or IMD14A | 3 |

**Footnotes:** ^a^As records from the CRD databases (DARE, NHS EED) are only published until 31^st^ March 2015, these databases were not re-searched as part of the clinical SLR update.

**Abbreviations:** CRD: Centre for Reviews and Dissemination; DARE: Database of Abstracts of Reviews of Effects; EED: Economic Evaluation Database; NHS: National Health Service; SLR: systematic literature review.

*Epidemiology SLR*

**Supplementary Table 8**: MEDLINE® search strategy for the epidemiology SLR (via Ovid.com)

| **#** | **Search Term** | **Hits 11/11/2021** |
| --- | --- | --- |
| **1** | ((Activated Phosph* adj8 (Delta* or syndrome*)) or (Activated PI3* adj5 delta*) or (PI3* adj5 syndrome*)).mp. | 206 |
| **2** | (Immunodeficiency 14 or immunodeficiency 36).mp. | 5 |
| **3** | ((P110* adj8 ((mutation and immunodeficien* and lymphadenopathy) or syndrome*)) or PASLI* or (mutat* adj5 (PIK3CD or PIK3R1)) or (Type* adj2 APDS) or APDS 1 or APDS1 or APDS 2 or APDS2 or IMD14A).mp. | 254 |
| **4** | exp Phosphatidylinositol 3-Kinases/ and (autosomal dominant disorder/ or primary immunodeficiency diseases/) | 68 |
| **5** | or/1-4 | 397 |
| **6** | exp Epidemiology/ or exp incidence/ or exp prevalence/ or (incidence or prevalence or epidemiolog*).mp. | 2980475 |
| **7** | 5 and 6 | 27 |

**Abbreviations:** MEDLINE: Medical Literature Analysis and Retrieval System Online; SLR: systematic literature review.

**Supplementary Table 9**: Embase® search strategy for the epidemiology SLR (via Ovid.com)

| **#** | **Search Term** | **Hits 11/11/2021** |
| --- | --- | --- |
| **1** | ((Activated Phosph* adj8 (Delta* or syndrome*)) or (Activated PI3* adj5 delta*) or (PI3* adj5 syndrome*)).mp. | 325 |
| **2** | (Immunodeficiency 14 or immunodeficiency 36).mp. | 6 |
| **3** | ((P110* adj8 ((mutation and immunodeficien* and lymphadenopathy) or syndrome*)) or PASLI* or (mutat* adj5 (PIK3CD or PIK3R1)) or (Type* adj2 APDS) or APDS 1 or APDS1 or APDS 2 or APDS2 or IMD14A).mp. | 521 |
| **4** | Exp phosphatidylinositol 3 kinase/ and (autosomal dominant disorder/ or immune deficiency/) | 316 |
| **5** | or/1-4 | 980 |
| **6** | exp Epidemiology/ or exp incidence/ or exp prevalence/ or (incidence or prevalence or epidemiolog*).mp. | 5298867 |
| **7** | 5 and 6 | 105 |

**Abbreviations:** Embase: Excerpta Medica Database; SLR: systematic literature review.

**Supplementary Table 10**: Cochrane Library search strategy for the epidemiology SLR (CDSR/CENTRAL, via Cochrane Library interface)

| **#** | **Search Term** | **Hits 12/11/2021** |
| --- | --- | --- |
| **1** | ((Activated Phosph* NEAR/8 (Delta* or syndrome*)) or (Activated PI3* NEAR/5 delta*) or (PI3* NEAR/5 syndrome*)):ti,ab,kw | 11 |
| **2** | ("Immunodeficiency 14" or "immunodeficiency 36"):ti,ab,kw | 1 |
| **3** | ((P110* NEAR/8 ((mutation and immunodeficien* and lymphadenopathy) or syndrome*)) or PASLI* or (mutat* NEAR/5 (PIK3CD or PIK3R1)) or (Type* NEAR/2 APDS) or APDS 1 or APDS1 or APDS 2 or APDS2 or IMD14A):ti,ab,kw | 40 |
| **4** | [mh "Phosphatidylinositol 3-Kinases"] and ([mh ^"autosomal dominant disorder"] or [mh ^"primary immunodeficiency diseases"]) | 0 |
| **5** | {Or #1-#4} | 50 |
| **6** | [mh "Epidemiology"] or [mh "incidence"] or [mh "prevalence"] or (incidence or prevalence or epidemiolog*):ti,ab,kw | 197658 |
| **7** | #5 and #6 | 7 |

**Abbreviations:** CDSR: Cochrane database of systematic reviews; CENTRAL: Cochrane Central Register of Controlled Trials; SLR: systematic literature review.

**Supplementary Table 11**: CRD database search strategy for the epidemiology SLR (via York.ac.uk/crd)^a^

| **#** | **Search Term** | **Hits 11/11/2021** |
| --- | --- | --- |
| **1** | Activated Phosph* or PI3* or Immunodeficiency 14 or immunodeficiency 36 or P110* or PASLI* or PIK3CD or PIK3R1 or APDS or IMD14A | 3 |

**Footnotes**^: a^As records from the CRD databases (DARE, NHS EED) are only published until 31^st^ March 2015, these databases were not re-searched as part of the economic SLR update.

**Abbreviations:** CRD: Centre for Reviews and Dissemination; DARE: Database of Abstract Reviews of Effects; EED: Economic Evaluation Database; HRQoL: heath-related quality of life; NHS: National Health Service; SLR: systematic literature review.

**Supplementary Table 12**: EconLit search strategy for the epidemiology SLR

| **#** | **Search Term** | **Hits 12/11/2021** |
| --- | --- | --- |
| **1** | ((Activated Phosph* adj8 (Delta* or syndrome*)) or (Activated PI3* adj5 delta*) or (PI3* adj5 syndrome*)).mp. | 0 |
| **2** | (Immunodeficiency 14 or immunodeficiency 36).mp. | 0 |
| **3** | ((P110* adj8 ((mutation and immunodeficien* and lymphadenopathy) or syndrome*)) or PASLI* or (mutat* adj5 (PIK3CD or PIK3R1)) or (Type* adj2 APDS) or APDS 1 or APDS1 or APDS 2 or APDS2 or IMD14A).mp. | 0 |
| **4** | or/1-3 | 0 |

**Abbreviations:** EconLit: Economics Literature; SLR: systematic literature review.

**Supplementary Table 13**: ScHARRHUD database search strategy for the epidemiology SLR (via https://www.scharrhud.org/)

| **#** | **Search Term** | **Hits 11/11/2021** |
| --- | --- | --- |
| **1** | Activated Phosph* or PI3* or Immunodeficiency 14 or immunodeficiency 36 or P110* or PASLI* or PIK3CD or PIK3R1 or APDS or IMD14A | 0 |

**Abbreviations:** ScHARRHUD: School of Health and Related Research Health Utilities Database; SLR: systematic literature review.

*Economic SLR*

**Supplementary Table 14**: MEDLINE^®^ search strategy for the original economic SLR (via Ovid.com)

| **#** | **Search Term** | **Hits**  **11/11/2021** |
| --- | --- | --- |
| **1** | ((Activated Phosph* adj8 (Delta* or syndrome*)) or (Activated PI3* adj5 delta*) or (PI3* adj5 syndrome*)).mp. | 206 |
| **2** | (Immunodeficiency 14 or immunodeficiency 36).mp. | 5 |
| **3** | ((P110* adj8 ((mutation and immunodeficien* and lymphadenopathy) or syndrome*)) or PASLI* or (mutat* adj5 (PIK3CD or PIK3R1)) or (Type* adj2 APDS) or APDS 1 or APDS1 or APDS 2 or APDS2 or IMD14A).mp. | 254 |
| **4** | exp Phosphatidylinositol 3-Kinases/ and (autosomal dominant disorder/ or primary immunodeficiency diseases/) | 68 |
| **5** | or/1-4 | 397 |
| **6** | Economics/ or exp "costs and cost analysis"/ or Value of life/ or exp economics, hospital/ or exp economics, medical/ or Economics, nursing/ or Economics, pharmaceutical/ or exp "fees and charges"/ or exp budgets/ or exp models, economic/ or Income/ or Remuneration/ or "Salaries and Fringe Benefits"/ or health resources/ or health planning/ or insurance, disability/ or insurance, health, reimbursement/ or drug utilization/ or technology assessment, biomedical/ or employment/ or work/ or absenteeism/ or efficiency/ or presenteeism/ | 505143 |
| **7** | (Economic* or pharmacoeconomic* or cost* or resource* or fiscal or funding or financial or finance* or price* or pricing or hospitalisation* or hospitalization* or stay* or office visits or (A&E adj2 visit) or ((A adj2 E) and visit) or ("accident and emergency" and visit) or mental health service* or "medication use" or expenditure* or budget* or expens* or earning* or salar* or wage* or pay or pays or paid or paying or payment* or income* or remunerat* or money or monetary or fee or fees or charg* or productiv* or (burden adj2 (illness or disease* or health or global)) or "societal impact" or "social impact" or employment or employed or employee* or unemploy* or "sick leave" or (productivity adj2 (cost* or loss*)) or ((carer or caregiver) adj2 burden)).mp. | 3225612 |
| **8** | 6 or 7 | 3288468 |
| **9** | 5 and 8 | 24 |

**Abbreviations:** MEDLINE: Medical Literature Analysis and Retrieval System Online; SLR: systematic literature review.

**Supplementary Table 15**: MEDLINE^®^ search strategy for the economic SLR update (via Ovid.com)

| **#** | **Search Term** | **Hits**  **18/05/2023** |
| --- | --- | --- |
| **1** | ((Activated Phosph* adj8 (Delta* or syndrome*)) or (Activated PI3* adj5 delta*) or (Activated PI 3* adj5 delta*) or (PI3* adj5 syndrome*)).ti,ab,kf. | 245 |
| **2** | (Immunodeficiency 14 or immunodeficiency 36 or IMD14A or IMD36).ti,ab,kf. | 5 |
| **3** | (P110* adj8 ((delta and activat* and mutation) or syndrome* or immunodeficien*)).ti,ab,kf. | 32 |
| **4** | (PASLI* or (mutat* adj5 (PIK3CD or PIK3R1)) or (Type* adj2 APDS) or (APDS and immunodeficien*) or APDS 1 or APDS1 or APDS 2 or APDS2).ti,ab,kf. | 314 |
| **5** | exp Phosphatidylinositol 3-Kinases/ and (primary immunodeficiency diseases/) | 87 |
| **6** | or/1-5 | 479 |
| **7** | Economics/ or exp "costs and cost analysis"/ or Value of life/ or exp economics, hospital/ or exp economics, medical/ or Economics, nursing/ or Economics, pharmaceutical/ or exp "fees and charges"/ or exp budgets/ or exp models, economic/ or Income/ or Remuneration/ or "Salaries and Fringe Benefits"/ or health resources/ or health planning/ or insurance, disability/ or insurance, health, reimbursement/ or drug utilization/ or technology assessment, biomedical/ or employment/ or work/ or absenteeism/ or efficiency/ or presenteeism/ | 525480 |
| **8** | (Economic* or pharmacoeconomic* or cost* or resource* or fiscal or funding or financial or finance* or price* or pricing or hospitalisation* or hospitalization* or stay* or office visits or (A&E adj2 visit) or ((A adj2 E) and visit) or ("accident and emergency" and visit) or mental health service* or "medication use" or expenditure* or budget* or expens* or earning* or salar* or wage* or pay or pays or paid or paying or payment* or income* or remunerat* or money or monetary or fee or fees or charg* or productiv* or (burden adj2 (illness or disease* or health or global)) or "societal impact" or "social impact" or employment or employed or employee* or unemploy* or "sick leave" or (productivity adj2 (cost* or loss*)) or ((carer or caregiver) adj2 burden)).mp. | 3633012 |
| **9** | 7 or 8 | 3696460 |
| **10** | 6 and 9 | 29 |

**Abbreviations:** MEDLINE: Medical Literature Analysis and Retrieval System Online; SLR: systematic literature review.

**Supplementary Table 16**: Embase^®^ search strategy for the original economic SLR (via Ovid.com)

| **#** | **Search Term** | **Hits**  **11/11/2021** |
| --- | --- | --- |
| **1** | ((Activated Phosph* adj8 (Delta* or syndrome*)) or (Activated PI3* adj5 delta*) or (PI3* adj5 syndrome*)).mp. | 325 |
| **2** | (Immunodeficiency 14 or immunodeficiency 36).mp. | 6 |
| **3** | ((P110* adj8 ((mutation and immunodeficien* and lymphadenopathy) or syndrome*)) or PASLI* or (mutat* adj5 (PIK3CD or PIK3R1)) or (Type* adj2 APDS) or APDS 1 or APDS1 or APDS 2 or APDS2 or IMD14A).mp. | 521 |
| **4** | Exp phosphatidylinositol 3 kinase/ and (autosomal dominant disorder/ or immune deficiency/) | 316 |
| **5** | or/1-4 | 980 |
| **6** | Socioeconomics/ or Cost benefit analysis/ or Cost effectiveness analysis/ or Cost of illness/ or Cost control/ or Economic aspect/ or Financial management/ or Health care cost/ or Health care financing/ or Health economics/ or Hospital cost/ or Cost minimization analysis/ or exp Economics/ or cost/ or drug cost/ or pharmacoeconomics/ or exp fee/ or budget/ or Economic Evaluation/ or cost utility analysis/ or hospitalization cost/ or nursing cost/ or health care planning/ or drug utilization/ or health insurance/ or biomedical technology assessment/ or employment/ or absenteeism/ or productivity/ or presenteeism/ or return to work/ | 1339966 |
| **7** | (Economic* or pharmacoeconomic* or cost* or resource* or fiscal or funding or financial or finance* or price* or pricing or hospitalisation* or hospitalization* or stay* or office visits or (A&E adj2 visit) or ((A adj2 E) and visit) or ("accident and emergency" and visit) or mental health service* or "medication use" or expenditure* or budget* or expens* or earning* or salar* or wage* or pay or pays or paid or paying or payment* or income* or remunerat* or money or monetary or fee or fees or charg* or productiv* or (burden adj2 (illness or disease* or health or global)) or "societal impact" or "social impact" or employment or employed or employee* or unemploy* or "sick leave" or (productivity adj2 (cost* or loss*)) or ((carer or caregiver) adj2 burden)).mp. | 4346760 |
| **8** | 6 or 7 | 4533304 |
| **9** | 5 and 8 | 77 |

**Abbreviations:** Embase: Excerpta Medica Database; SLR: systematic literature review.

**Supplementary Table 17**: Embase^®^ search strategy for the economic SLR update (via Ovid.com)

| **#** | **Search Term** | **Hits**  **18/05/2023** |
| --- | --- | --- |
| **1** | ((Activated Phosph* adj8 (Delta* or syndrome*)) or (Activated PI3* adj5 delta*) or (Activated PI 3* adj5 delta*) or (PI3* adj5 syndrome*)).ti,ab,kf. | 366 |
| **2** | (Immunodeficiency 14 or immunodeficiency 36 or IMD14A or IMD36).ti,ab,kf. | 7 |
| **3** | (P110* adj8 ((delta and activat* and mutation) or syndrome* or immunodeficien*)).ti,ab,kf. | 60 |
| **4** | (PASLI* or (mutat* adj5 (PIK3CD or PIK3R1)) or (Type* adj2 APDS) or (APDS and immunodeficien*) or APDS 1 or APDS1 or APDS 2 or APDS2).ti,ab,kf. | 644 |
| **5** | exp phosphatidylinositol 3 Kinases/ and (autosomal dominant disorder/ or immune deficiency) | 466 |
| **6** | or/1-5 | 1131 |
| **7** | Socioeconomics/ or Cost benefit analysis/ or Cost effectiveness analysis/ or Cost of illness/ or Cost control/ or Economic aspect/ or Financial management/ or Health care cost/ or Health care financing/ or Health economics/ or Hospital cost/ or Cost minimization analysis/ or exp Economics/ or cost/ or drug cost/ or pharmacoeconomics/ or exp fee/ or budget/ or Economic Evaluation/ or cost utility analysis/ or hospitalization cost/ or nursing cost/ or health care planning/ or drug utilization/ or health insurance/ or biomedical technology assessment/ or employment/ or absenteeism/ or productivity/ or presenteeism/ or return to work/ | 1446300 |
| **8** | (Economic* or pharmacoeconomic* or cost* or resource* or fiscal or funding or financial or finance* or price* or pricing or hospitalisation* or hospitalization* or stay* or office visits or (A&E adj2 visit) or ((A adj2 E) and visit) or ("accident and emergency" and visit) or mental health service* or "medication use" or expenditure* or budget* or expens* or earning* or salar* or wage* or pay or pays or paid or paying or payment* or income* or remunerat* or money or monetary or fee or fees or charg* or productiv* or (burden adj2 (illness or disease* or health or global)) or "societal impact" or "social impact" or employment or employed or employee* or unemploy* or "sick leave" or (productivity adj2 (cost* or loss*)) or ((carer or caregiver) adj2 burden)).mp. | 4994309 |
| **9** | 7 or 8 | 5194680 |
| **10** | 6 and 9 | 103 |

**Abbreviations:** Embase: Excerpta Medica Database; SLR: systematic literature review.

**Supplementary Table 18**: Cochrane Library search strategy for the original economic SLR (CDSR/CENTRAL, via Cochrane Library interface)

| **#** | **Search Term** | **Hits**  **11/11/2021** |
| --- | --- | --- |
| **1** | ((Activated Phosph* NEAR/8 (Delta* or syndrome*)) or (Activated PI3* NEAR/5 delta*) or (PI3* NEAR/5 syndrome*)):ti,ab,kw | 11 |
| **2** | ("Immunodeficiency 14" or "immunodeficiency 36"):ti,ab,kw | 1 |
| **3** | ((P110* NEAR/8 ((mutation and immunodeficien* and lymphadenopathy) or syndrome*)) or PASLI* or (mutat* NEAR/5 (PIK3CD or PIK3R1)) or (Type* NEAR/2 APDS) or APDS 1 or APDS1 or APDS 2 or APDS2 or IMD14A):ti,ab,kw | 40 |
| **4** | [mh "Phosphatidylinositol 3-Kinases"] and ([mh ^"autosomal dominant disorder"] or [mh ^"primary immunodeficiency diseases"]) | 0 |
| **5** | {Or #1-#4} | 50 |
| **6** | [mh ^"economics"] or [mh "costs and cost analysis"] or [mh ^"value of life"] or [mh "economics, hospital"] or [mh "economics, medical"] or [mh ^"economics, nursing"] or [mh ^"economics, pharmaceutical"] or [mh "fees and charges"] or [mh "budgets"] or [mh "models, economic"] or [mh ^"Income"] or [mh ^"Remuneration"] or [mh ^"Salaries and Fringe Benefits"] or [mh ^"health resources"] or [mh ^"health planning"] or [mh ^"insurance, disability"] or [mh ^"insurance, health, reimbursement"] or [mh ^"drug utilization"] or [mh ^"technology assessment, biomedical"] or [mh ^"employment"] or [mh ^"work"] or [mh ^"absenteeism"] or [mh ^"efficiency"] or [mh ^"presenteeism"] | 13793 |
| **7** | (Economic* or pharmacoeconomic* or cost* or resource* or fiscal or funding or financial or finance* or price* or pricing or hospitalisation* or hospitalization* or stay* or office visits or (A&E NEAR/5 visit) or ((A NEAR/5 E) and visit) or ("accident and emergency" and visit) or physiotherapy or mobility aid* or mental health service* or "medication use" or expenditure* or budget* or expens* or earning* or salar* or wage* or pay or pays or paid or paying or payment* or income* or remunerat* or money or monetary or fee or fees or charg* or productiv* or (burden NEAR/2 (illness or disease* or health or global)) or "societal impact" or "social impact" or employment or employed or employee* or unemploy* or "sick leave" or (productivity NEAR/2 (cost* or loss*)) or ((carer or caregiver) NEAR/2 burden)):ti,ab,kw | 251581 |
| **8** | #6 or #7 | 252309 |
| **9** | #5 and #8 | 8 |

**Abbreviations:** CDSR: Cochrane database of systematic reviews; CENTRAL: Cochrane Central Register of Controlled Trials; SLR: systematic literature review.

**Supplementary Table 19**: Cochrane Library search strategy for the economic SLR update (CDSR/CENTRAL, via Cochrane Library interface)

| **#** | **Search Term** | **Hits**  **18/05/2023** |
| --- | --- | --- |
| **1** | ((Activated Phosph* NEAR/8 (Delta* or syndrome*)) or (Activated PI3* NEAR/5 delta*) or (Activated PI 3* NEAR/5 delta*) or (PI3* NEAR/5 syndrome*)):ti,ab,kw | 17 |
| **2** | ("Immunodeficiency 14" or "immunodeficiency 36" or IMD14A or IMD36):ti,ab,kw | 1 |
| **3** | (P110* NEAR/8 ((delta and activat* and mutation) or syndrome* or immunodeficien*)):ti,ab,kw | 5 |
| **4** | (PASLI* or (mutat* NEAR/5 (PIK3CD or PIK3R1)) or (Type* NEAR/2 APDS) or (APDS and immunodeficien*) or APDS 1 or APDS1 or APDS 2 or APDS2):ti,ab,kw | 46 |
| **5** | [mh "Phosphatidylinositol 3-Kinases"] and [mh ^"primary immunodeficiency diseases"] | 0 |
| **6** | {Or #1-#5} | 61 |
| **7** | [mh ^"economics"] or [mh "costs and cost analysis"] or [mh ^"value of life"] or [mh "economics, hospital"] or [mh "economics, medical"] or [mh ^"economics, nursing"] or [mh ^"economics, pharmaceutical"] or [mh "fees and charges"] or [mh "budgets"] or [mh "models, economic"] or [mh ^"Income"] or [mh ^"Remuneration"] or [mh ^"Salaries and Fringe Benefits"] or [mh ^"health resources"] or [mh ^"health planning"] or [mh ^"insurance, disability"] or [mh ^"insurance, health, reimbursement"] or [mh ^"drug utilization"] or [mh ^"technology assessment, biomedical"] or [mh ^"employment"] or [mh ^"work"] or [mh ^"absenteeism"] or [mh ^"efficiency"] or [mh ^"presenteeism"] | 18419 |
| **8** | (Economic* or pharmacoeconomic* or cost* or resource* or fiscal or funding or financial or finance* or price* or pricing or hospitalisation* or hospitalization* or stay* or office visits or (A&E NEAR/5 visit) or ((A NEAR/5 E) and visit) or ("accident and emergency" and visit) or physiotherapy or mobility aid* or mental health service* or "medication use" or expenditure* or budget* or expens* or earning* or salar* or wage* or pay or pays or paid or paying or payment* or income* or remunerat* or money or monetary or fee or fees or charg* or productiv* or (burden NEAR/2 (illness or disease* or health or global)) or "societal impact" or "social impact" or employment or employed or employee* or unemploy* or "sick leave" or (productivity NEAR/2 (cost* or loss*)) or ((carer or caregiver) NEAR/2 burden)):ti,ab,kw | 287881 |
| **9** | #6 or #7 | 288828 |
| **10** | #6 and #9 | 8 |

**Abbreviations**: CDSR: Cochrane database of systematic reviews; CENTRAL: Cochrane Central Register of Controlled Trials; SLR: systematic literature review.

**Supplementary Table 20**: CRD database search strategy for the original economic SLR (via York.ac.uk/crd)^a^

| **#** | **Search Term** | **Hits**  **11/11/2021** |
| --- | --- | --- |
| **1** | Activated Phosph* or PI3* or Immunodeficiency 14 or immunodeficiency 36 or P110* or PASLI* or PIK3CD or PIK3R1 or APDS or IMD14A | 3 |

**Footnote:** ^a^As records from the CRD databases (DARE, NHS EED) are only published until 31^st^ March 2015, these databases were not re-searched as part of the economic SLR update.

**Abbreviations:** CRD: Centre for Reviews and Dissemination; DARE: Database of Abstracts of Reviews of Effects; EED: Economic Evaluation Database; NHS: National Health Service; SLR: systematic literature review.

**Supplementary Table 21**: EconLit search strategy for the original economic SLR

| **#** | **Search Term** | **Hits**  **12/11/2021** |
| --- | --- | --- |
| **1** | ((Activated Phosph* adj8 (Delta* or syndrome*)) or (Activated PI3* adj5 delta*) or (PI3* adj5 syndrome*)).mp. | 0 |
| **2** | (Immunodeficiency 14 or immunodeficiency 36).mp. | 0 |
| **3** | ((P110* adj8 ((mutation and immunodeficien* and lymphadenopathy) or syndrome*)) or PASLI* or (mutat* adj5 (PIK3CD or PIK3R1)) or (Type* adj2 APDS) or APDS 1 or APDS1 or APDS 2 or APDS2 or IMD14A).mp. | 0 |
| **4** | or/1-3 | 0 |

**Abbreviations:** EconLit: Economics Literature; SLR: systematic literature review.

**Supplementary Table 22**: EconLit search strategy for the economic SLR update

| **#** | **Search Term** | **Hits**  **18/05/2023** |
| --- | --- | --- |
| **1** | ((Activated Phosph* adj8 (Delta* or syndrome*)) or (Activated PI3* adj5 delta*) or (Activated PI 3* adj5 delta*) or (PI3* adj5 syndrome*)).ti,ab,kf. | 0 |
| **2** | (Immunodeficiency 14 or immunodeficiency 36 or IMD14A or IMD36).ti,ab,kf. | 0 |
| **3** | (PASLI* or (mutat* adj5 (PIK3CD or PIK3R1)) or (Type* adj2 APDS) or (APDS and immunodeficien*) or APDS 1 or APDS1 or APDS 2 or APDS2).ti,ab,kf. | 0 |
| **4** | or/1-3 | 0 |

**Abbreviations:** EconLit: Economics Literature; SLR: systematic literature review.

**Supplementary Table 23**: ScHARRHUD database search strategy for the original economic SLR (via https://www.scharrhud.org/)

| **#** | **Search Term** | **Hits**  **12/11/2021** |
| --- | --- | --- |
| **1** | Activated Phosph* or PI3* or Immunodeficiency 14 or immunodeficiency 36 or P110* or PASLI* or PIK3CD or PIK3R1 or APDS or IMD14A | 0 |

**Abbreviations:** ScHARRHUD: School of Health and Related Research Health Utilities Database; SLR: systematic literature review.

**Supplementary Table 24**: ScHARRHUD database search strategy for the economic SLR update (via https://www.scharrhud.org/)

| **#** | **Search Term** | **Hits**  **18/05/2023** |
| --- | --- | --- |
| **1** | Activated Phosph* or PI3* or Immunodeficiency 14 or immunodeficiency 36 or P110* or PASLI* or PIK3CD or PIK3R1 or APDS or IMD14A | 0 |

**Abbreviations:** ScHARRHUD: School of Health and Related Research Health Utilities Database; SLR: systematic literature review.

*HRQoL SLR*

**Supplementary Table 25**: MEDLINE^®^ search strategy for the original HRQoL/utility SLR (via Ovid.com)

| **#** | **Search Term** | **Hits**  **11/11/2021** |
| --- | --- | --- |
| **1** | ((Activated Phosph* adj8 (Delta* or syndrome*)) or (Activated PI3* adj5 delta*) or (PI3* adj5 syndrome*)).mp. | 206 |
| **2** | (Immunodeficiency 14 or immunodeficiency 36).mp. | 5 |
| **3** | ((P110* adj8 ((mutation and immunodeficien* and lymphadenopathy) or syndrome*)) or PASLI* or (mutat* adj5 (PIK3CD or PIK3R1)) or (Type* adj2 APDS) or APDS 1 or APDS1 or APDS 2 or APDS2 or IMD14A).mp. | 254 |
| **4** | exp Phosphatidylinositol 3-Kinases/ and (autosomal dominant disorder/ or primary immunodeficiency diseases/) | 68 |
| **5** | or/1-4 | 397 |
| **6** | exp quality of life/ or exp quality adjusted life years/ or exp health surveys/ or Value of Life/ or exp Disability Evaluation/ or exp models, economic/ or exp questionnaire/ or exp visual analog scale/ | 1336300 |
| **7** | (quality of life or utilit* or quality adjusted or adjusted life or qaly* or qald* or qale* or qtime* or life year or life years or disability adjusted life or daly* or short form* or shortform* or sf* or hql or qol or hrql or hqol or h qol or hrqol or hr qol or hye or hyes or (health* adj2 year* adj2 equivalent*) or pqol or qls or quality of wellbeing or quality of well being or index of wellbeing or index of well being or qwb or nottingham health profile* or sickness impact profile or ((health or illness) adj3 stat*) or (preference* adj3 (score* or scoring or valu* or measur* or evaluat* or scale* or instrument* or weight or weights or weighting or information or data or unit or units or health* or life or estimat* or elicit* or disease* or mean or cost* or expenditure* or gain or gains or loss or losses or lost or analysis or index* or indices or overall or reported or calculat* or range* or increment* or state or states or status)) or disutilit* or HSUV or HSUVs or rosser or willingness to pay or standard gamble* or sg or time trade off or time tradeoff or timetradeoff or tto or hui or hui1 or hui2 or hui3 or eq or euroqol* or euro qol* or eq5d or eq-5d or eq5-d or euroqual* or euro qual* or eq-sdq or eqsdq or duke health profile or functional status questionnaire or dartmouth coop functional health assessment* or multiattribute* or multi attribute* or 15D or 15-D or 15 dimension or medical outcome study or RAND36 or RAND12 or (health adj3 (status or index)) or PedsQL or Visual analog* scale or VAS).mp. | 1030779 |
| **8** | 6 or 7 | 2013500 |
| **9** | 5 and 8 | 12 |

**Abbreviations:** HRQoL: heath-related quality of life; MEDLINE: Medical Literature Analysis and Retrieval System Online; SLR: systematic literature review.

**Supplementary Table 26**: MEDLINE^®^ search strategy for the HRQoL/utility SLR update (via Ovid.com)

| **#** | **Search Term** | **Hits**  **18/05/2023** |
| --- | --- | --- |
| **1** | ((Activated Phosph* adj8 (Delta* or syndrome*)) or (Activated PI3* adj5 delta*) or (Activated PI 3* adj5 delta*) or (PI3* adj5 syndrome*)).ti,ab,kf. | 245 |
| **2** | (Immunodeficiency 14 or immunodeficiency 36 or IMD14A or IMD36).ti,ab,kf. | 5 |
| **3** | (P110* adj8 ((delta and activat* and mutation) or syndrome* or immunodeficien*)).ti,ab,kf. | 32 |
| **4** | (PASLI* or (mutat* adj5 (PIK3CD or PIK3R1)) or (Type* adj2 APDS) or (APDS and immunodeficien*) or APDS 1 or APDS1 or APDS 2 or APDS2).ti,ab,kf. | 314 |
| **5** | exp Phosphatidylinositol 3-Kinases/ and (primary immunodeficiency diseases/) | 87 |
| **6** | or/1-5 | 479 |
| **7** | exp quality of life/ or exp quality adjusted life years/ or exp health surveys/ or Value of Life/ or exp Disability Evaluation/ or exp models, economic/ or exp questionnaire/ or exp visual analog scale/ | 1441460 |
| **8** | (quality of life or utilit* or quality adjusted or adjusted life or qaly* or qald* or qale* or qtime* or life year or life years or disability adjusted life or daly* or short form* or shortform* or sf* or hql or qol or hrql or hqol or h qol or hrqol or hr qol or hye or hyes or (health* adj2 year* adj2 equivalent*) or pqol or qls or quality of wellbeing or quality of well being or index of wellbeing or index of well being or qwb or nottingham health profile* or sickness impact profile or ((health or illness) adj3 stat*) or (preference* adj3 (score* or scoring or valu* or measur* or evaluat* or scale* or instrument* or weight or weights or weighting or information or data or unit or units or health* or life or estimat* or elicit* or disease* or mean or cost* or expenditure* or gain or gains or loss or losses or lost or analysis or index* or indices or overall or reported or calculat* or range* or increment* or state or states or status)) or disutilit* or HSUV or HSUVs or rosser or willingness to pay or standard gamble* or sg or time trade off or time tradeoff or timetradeoff or tto or hui or hui1 or hui2 or hui3 or eq or euroqol* or euro qol* or eq5d or eq-5d or eq5-d or euroqual* or euro qual* or eq-sdq or eqsdq or duke health profile or functional status questionnaire or dartmouth coop functional health assessment* or multiattribute* or multi attribute* or 15D or 15-D or 15 dimension or medical outcome study or RAND36 or RAND12 or (health adj3 (status or index)) or PedsQL or Visual analog* scale or VAS).mp. | 1156847 |
| **9** | 7 or 8 | 2198138 |
| **10** | 6 and 9 | 19 |

**Abbreviations:** HRQoL: heath-related quality of life; MEDLINE: Medical Literature Analysis and Retrieval System Online; SLR: systematic literature review.

**Supplementary Table 27**: Embase^®^ search strategy for the original HRQoL/utility SLR (via Ovid.com)

| **#** | **Search Term** | **Hits**  **11/11/2021** |
| --- | --- | --- |
| **1** | ((Activated Phosph* adj8 (Delta* or syndrome*)) or (Activated PI3* adj5 delta*) or (PI3* adj5 syndrome*)).mp. | 325 |
| **2** | (Immunodeficiency 14 or immunodeficiency 36).mp. | 6 |
| **3** | ((P110* adj8 ((mutation and immunodeficien* and lymphadenopathy) or syndrome*)) or PASLI* or (mutat* adj5 (PIK3CD or PIK3R1)) or (Type* adj2 APDS) or APDS 1 or APDS1 or APDS 2 or APDS2 or IMD14A).mp. | 521 |
| **4** | Exp phosphatidylinositol 3 kinase/ and (autosomal dominant disorder/ or immune deficiency/) | 316 |
| **5** | or/1-4 | 980 |
| **6** | socioeconomics/ or exp Quality of Life/ or exp Quality-Adjusted Life Year/ or nottingham health profile/ or sickness impact profile/ or exp health survey/ or exp Disability Evaluation/ or exp models, economic/ or exp questionnaire/ or exp visual analog scale/ | 1751523 |
| **7** | (quality of life or utilit* or quality adjusted or adjusted life or qaly* or qald* or qale* or qtime* or life year or life years or disability adjusted life or daly* or short form* or shortform* or sf* or hql or qol or hrql or hqol or h qol or hrqol or hr qol or hye or hyes or (health* adj2 year* adj2 equivalent*) or pqol or qls or quality of wellbeing or quality of well being or index of wellbeing or index of well being or qwb or nottingham health profile* or sickness impact profile or ((health or illness) adj3 stat*) or (preference* adj3 (score* or scoring or valu* or measur* or evaluat* or scale* or instrument* or weight or weights or weighting or information or data or unit or units or health* or life or estimat* or elicit* or disease* or mean or cost* or expenditure* or gain or gains or loss or losses or lost or analysis or index* or indices or overall or reported or calculat* or range* or increment* or state or states or status)) or disutilit* or HSUV or HSUVs or rosser or willingness to pay or standard gamble* or sg or time trade off or time tradeoff or timetradeoff or tto or hui or hui1 or hui2 or hui3 or eq or euroqol* or euro qol* or eq5d or eq-5d or eq5-d or euroqual* or euro qual* or eq-sdq or eqsdq or duke health profile or functional status questionnaire or dartmouth coop functional health assessment* or multiattribute* or multi attribute* or 15D or 15-D or 15 dimension or medical outcome study or RAND36 or RAND12 or (health adj3 (status or index)) or PedsQL or Visual analog* scale or VAS).mp. | 1522431 |
| **8** | 6 or 7 | 2529360 |
| **9** | 5 and 8 | 34 |

**Abbreviations:** Embase: Excerpta Medica Database; HRQoL: heath-related quality of life; SLR: systematic literature review.

**Supplementary Table 28**: Embase^®^ search strategy for the HRQoL/utility SLR update (via Ovid.com)

| **#** | **Search Term** | **Hits**  **18/05/2023** |
| --- | --- | --- |
| **1** | ((Activated Phosph* adj8 (Delta* or syndrome*)) or (Activated PI3* adj5 delta*) or (Activated PI 3* adj5 delta*) or (PI3* adj5 syndrome*)).ti,ab,kf. | 366 |
| **2** | (Immunodeficiency 14 or immunodeficiency 36 or IMD14A or IMD36).ti,ab,kf. | 7 |
| **3** | (P110* adj8 ((delta and activat* and mutation) or syndrome* or immunodeficien*)).ti,ab,kf. | 60 |
| **4** | (PASLI* or (mutat* adj5 (PIK3CD or PIK3R1)) or (Type* adj2 APDS) or (APDS and immunodeficien*) or APDS 1 or APDS1 or APDS 2 or APDS2).ti,ab,kf. | 644 |
| **5** | exp phosphatidylinositol 3 Kinases/ and (autosomal dominant disorder/ or immune deficiency) | 466 |
| **6** | Or/1-5 | 1231 |
| **7** | socioeconomics/ or exp Quality of Life/ or exp Quality-Adjusted Life Year/ or nottingham health profile/ or sickness impact profile/ or exp health survey/ or exp Disability Evaluation/ or exp models, economic/ or exp questionnaire/ or exp visual analog scale/ | 1887300 |
| **8** | (quality of life or utilit* or quality adjusted or adjusted life or qaly* or qald* or qale* or qtime* or life year or life years or disability adjusted life or daly* or short form* or shortform* or sf* or hql or qol or hrql or hqol or h qol or hrqol or hr qol or hye or hyes or (health* adj2 year* adj2 equivalent*) or pqol or qls or quality of wellbeing or quality of well being or index of wellbeing or index of well being or qwb or nottingham health profile* or sickness impact profile or ((health or illness) adj3 stat*) or (preference* adj3 (score* or scoring or valu* or measur* or evaluat* or scale* or instrument* or weight or weights or weighting or information or data or unit or units or health* or life or estimat* or elicit* or disease* or mean or cost* or expenditure* or gain or gains or loss or losses or lost or analysis or index* or indices or overall or reported or calculat* or range* or increment* or state or states or status)) or disutilit* or HSUV or HSUVs or rosser or willingness to pay or standard gamble* or sg or time trade off or time tradeoff or timetradeoff or tto or hui or hui1 or hui2 or hui3 or eq or euroqol* or euro qol* or eq5d or eq-5d or eq5-d or euroqual* or euro qual* or eq-sdq or eqsdq or duke health profile or functional status questionnaire or dartmouth coop functional health assessment* or multiattribute* or multi attribute* or 15D or 15-D or 15 dimension or medical outcome study or RAND36 or RAND12 or (health adj3 (status or index)) or PedsQL or Visual analog* scale or VAS).mp. | 1758797 |
| **9** | 7 or 8 | 2788966 |
| **10** | 6 and 9 | 45 |

**Abbreviations:** Embase: Excerpta Medica Database; HRQoL: heath-related quality of life; SLR: systematic literature review.

**Supplementary Table 29**: Cochrane Library search strategy for the original HRQoL/utility SLR (CDSR/CENTRAL, via Cochrane Library interface)

| **#** | **Search Term** | **Hits**  **12/11/2021** |
| --- | --- | --- |
| **1** | ((Activated Phosph* NEAR/8 (Delta* or syndrome*)) or (Activated PI3* NEAR/5 delta*) or (PI3* NEAR/5 syndrome*)):ti,ab,kw | 11 |
| **2** | ("Immunodeficiency 14" or "immunodeficiency 36"):ti,ab,kw | 1 |
| **3** | ((P110* NEAR/8 ((mutation and immunodeficien* and lymphadenopathy) or syndrome*)) or PASLI* or (mutat* NEAR/5 (PIK3CD or PIK3R1)) or (Type* NEAR/2 APDS) or APDS 1 or APDS1 or APDS 2 or APDS2 or IMD14A):ti,ab,kw | 40 |
| **4** | [mh "Phosphatidylinositol 3-Kinases"] and ([mh ^"autosomal dominant disorder"] or [mh ^"primary immunodeficiency diseases"]) | 0 |
| **5** | {Or #1-#4} | 50 |
| **6** | [mh "quality of life"] or [mh "quality adjusted life years"] or [mh "health survey"] or [mh ^"Value of Life"] or [mh "Disability Evaluation"] or [mh "models, economic"] or [mh "questionnaire"] or [mh "visual analog scale"] | 78928 |
| **7** | (quality of life or utilit* or quality adjusted or adjusted life or qaly* or qald* or qale* or qtime* or life year or life years or disability adjusted life or daly* or short form* or shortform* or sf* or hql or qol or hrql or hqol or h qol or hrqol or hr qol or hye or hyes or (health* NEAR/2 year* NEAR/2 equivalent*) or pqol or qls or quality of wellbeing or quality of well being or index of wellbeing or index of well being or qwb or nottingham health profile* or sickness impact profile or ((health or illness) NEAR/3 stat*) or (preference* NEAR/3 (score* or scoring or valu* or measur* or evaluat* or scale* or instrument* or weight or weights or weighting or information or data or unit or units or health* or life or estimat* or elicit* or disease* or mean or cost* or expenditure* or gain or gains or loss or losses or lost or analysis or index* or indices or overall or reported or calculat* or range* or increment* or state or states or status)) or disutilit* or HSUV or HSUVs or rosser or willingness to pay or standard gamble* or sg or time trade off or time tradeoff or timetradeoff or tto or hui or hui1 or hui2 or hui3 or eq or euroqol* or euro qol* or eq5d or eq-5d or eq5* or euroqual* or euro qual* or eqsdq or duke health profile or functional status questionnaire or dartmouth coop functional health assessment* or multiattribute* or multi attribute* or 15D or 15 D or 15 dimension or medical outcome study or RAND36 or RAND12 or (health NEAR/3 (status or index)) or PedsQL or Visual analog* scale or VAS):ti,ab,kw | 326708 |
| **8** | #6 or #7 | 364830 |
| **9** | #5 and #8 | 13 |

**Abbreviations:** CDSR: Cochrane database of systematic reviews; CENTRAL: Cochrane Central Register of Controlled Trials; HRQoL: heath-related quality of life; SLR: systematic literature review.

**Supplementary Table 30**: Cochrane Library search strategy for the HRQoL/utility SLR update (CDSR/CENTRAL, via Cochrane Library interface)

| **#** | **Search Term** | **Hits**  **18/05/2023** |
| --- | --- | --- |
| **1** | ((Activated Phosph* NEAR/8 (Delta* or syndrome*)) or (Activated PI3* NEAR/5 delta*) or (Activated PI 3* NEAR/5 delta*) or (PI3* NEAR/5 syndrome*)):ti,ab,kw | 17 |
| **2** | ("Immunodeficiency 14" or "immunodeficiency 36" or IMD14A or IMD36):ti,ab,kw | 1 |
| **3** | (P110* NEAR/8 ((delta and activat* and mutation) or syndrome* or immunodeficien*)):ti,ab,kw | 5 |
| **4** | (PASLI* or (mutat* NEAR/5 (PIK3CD or PIK3R1)) or (Type* NEAR/2 APDS) or (APDS and immunodeficien*) or APDS 1 or APDS1 or APDS 2 or APDS2):ti,ab,kw | 46 |
| **5** | [mh "Phosphatidylinositol 3-Kinases"] and [mh ^"primary immunodeficiency diseases] | 0 |
| **6** | {Or #1-#5} | 61 |
| **7** | [mh "quality of life"] or [mh "quality adjusted life years"] or [mh "health survey"] or [mh ^"Value of Life"] or [mh "Disability Evaluation"] or [mh "models, economic"] or [mh "questionnaire"] or [mh "visual analog scale"] | 110819 |
| **8** | (quality of life or utilit* or quality adjusted or adjusted life or qaly* or qald* or qale* or qtime* or life year or life years or disability adjusted life or daly* or short form* or shortform* or sf* or hql or qol or hrql or hqol or h qol or hrqol or hr qol or hye or hyes or (health* NEAR/2 year* NEAR/2 equivalent*) or pqol or qls or quality of wellbeing or quality of well being or index of wellbeing or index of well being or qwb or nottingham health profile* or sickness impact profile or ((health or illness) NEAR/3 stat*) or (preference* NEAR/3 (score* or scoring or valu* or measur* or evaluat* or scale* or instrument* or weight or weights or weighting or information or data or unit or units or health* or life or estimat* or elicit* or disease* or mean or cost* or expenditure* or gain or gains or loss or losses or lost or analysis or index* or indices or overall or reported or calculat* or range* or increment* or state or states or status)) or disutilit* or HSUV or HSUVs or rosser or willingness to pay or standard gamble* or sg or time trade off or time tradeoff or timetradeoff or tto or hui or hui1 or hui2 or hui3 or eq or euroqol* or euro qol* or eq5d or eq-5d or eq5* or euroqual* or euro qual* or eqsdq or duke health profile or functional status questionnaire or dartmouth coop functional health assessment* or multiattribute* or multi attribute* or 15D or 15 D or 15 dimension or medical outcome study or RAND36 or RAND12 or (health NEAR/3 (status or index)) or PedsQL or Visual analog* scale or VAS):ti,ab,kw | 380566 |
| **9** | #7 or #8 | 427180 |
| **10** | #6 and #9 | 13 |

**Abbreviations:** CDSR: Cochrane database of systematic reviews; CENTRAL: Cochrane Central Register of Controlled Trials; HRQoL: heath-related quality of life; SLR: systematic literature review.

**Supplementary Table 31**: CRD database search strategy for the original HRQoL/utility SLR (via York.ac.uk/crd)^a^

| **#** | **Search Term** | **Hits**  **11/11/2021** |
| --- | --- | --- |
| **1** | Activated Phosph* or PI3* or Immunodeficiency 14 or immunodeficiency 36 or P110* or PASLI* or PIK3CD or PIK3R1 or APDS or IMD14A | 3 |

**Footnote:** ^a^As records from the CRD databases (DARE, NHS EED) are only published until 31^st^ March 2015, these databases were not re-searched as part of the economic SLR update.

**Abbreviations:** CRD: Centre for Reviews and Dissemination; DARE: Database of Abstract Reviews of Effects; EED: Economic Evaluation Database; HRQoL: heath-related quality of life; NHS: National Health Service; SLR: systematic literature review.

**Supplementary Table 32**: EconLit search strategy for the original HRQoL/utility SLR

| **#** | **Search Term** | **Hits**  **12/11/2021** |
| --- | --- | --- |
| **1** | ((Activated Phosph* adj8 (Delta* or syndrome*)) or (Activated PI3* adj5 delta*) or (PI3* adj5 syndrome*)).mp. | 0 |
| **2** | (Immunodeficiency 14 or immunodeficiency 36).mp. | 0 |
| **3** | ((P110* adj8 ((mutation and immunodeficien* and lymphadenopathy) or syndrome*)) or PASLI* or (mutat* adj5 (PIK3CD or PIK3R1)) or (Type* adj2 APDS) or APDS 1 or APDS1 or APDS 2 or APDS2 or IMD14A).mp. | 0 |
| **4** | or/1-3 | 0 |

**Abbreviations:** EconLit: Economics Literature; HRQoL: heath-related quality of life; SLR: systematic literature review.

**Supplementary Table 33**: EconLit search strategy for the HRQoL/utility SLR update

| **#** | **Search Term** | **Hits**  **18/05/2023** |
| --- | --- | --- |
| **1** | ((Activated Phosph* adj8 (Delta* or syndrome*)) or (Activated PI3* adj5 delta*) or (Activated PI 3* adj5 delta*) or (PI3* adj5 syndrome*)).ti,ab,kf. | 0 |
| **2** | (Immunodeficiency 14 or immunodeficiency 36 or IMD14A or IMD36).ti,ab,kf. | 0 |
| **3** | (PASLI* or (mutat* adj5 (PIK3CD or PIK3R1)) or (Type* adj2 APDS) or (APDS and immunodeficien*) or APDS 1 or APDS1 or APDS 2 or APDS2).ti,ab,kf. | 0 |
| **4** | or/1-3 | 0 |

**Abbreviations:** EconLit: Economics Literature; HRQoL: heath-related quality of life; SLR: systematic literature review.

**Supplementary Table 34**: ScHARRHUD database search strategy for the original HRQoL/utility SLR (via https://www.scharrhud.org/)

| **#** | **Search Term** | **Hits**  **11/11/2021** |
| --- | --- | --- |
| **1** | Activated Phosph* or PI3* or Immunodeficiency 14 or immunodeficiency 36 or P110* or PASLI* or PIK3CD or PIK3R1 or APDS or IMD14A | 0 |

**Abbreviations:** HRQoL: heath-related quality of life; ScHARRHUD: School of Health and Related Research Health Utilities Database; SLR: systematic literature review.

**Supplementary Table 35**: ScHARRHUD database search strategy for the HRQoL/utility SLR update (via https://www.scharrhud.org/)

| **#** | **Search Term** | **Hits**  **18/05/2023** |
| --- | --- | --- |
| **1** | Activated Phosph* or PI3* or Immunodeficiency 14 or immunodeficiency 36 or P110* or PASLI* or PIK3CD or PIK3R1 or APDS or IMD14A | 0 |

**Abbreviations:** HRQoL: heath-related quality of life; ScHARRHUD: School of Health and Related Research Health Utilities Database; SLR: systematic literature review.

Search Strategy and Results for Grey Literature

*Conference searches*

**Supplementary Table 36**: Conference search strategy and results (clinical and economic SLRs: hand-searched from 2019 onwards)

| **Conference/ Organisation** | **Meetings searched** | **Hits^a^** | **Records included^a^** |
| --- | --- | --- | --- |
| International Society for Pharmacoeconomics and Outcomes Research (ISPOR; all meetings) | - Boston 2023 - Vienna 2022 - Asia Pacific, Virtual 2022 - Washington 2022 - Copenhagen 2021   <https://www.ispor.org/heor-resources/presentations-database/search> | 1 | 1 |
|  | - 2021 Montreal - 2021 Virtual Europe   <https://www.ispor.org/heor-resources/presentations-database/search> | 1 | 0 |
| ESID | - ESID [20th Biennial Meeting 2022 - Gothenburg, Sweden](https://esid.org/News-Events/ESID-Meetings/ESID-Biennial-Meeting/19th-Biennial-Meeting-2020-Birmingham-UK) - ESID 19th Biennial Meeting 2020 - Birmingham, UK   Unable to find abstract books | NA | NA |
| European Academy of Allergy and Clinical Immunology (EAACI) | 2022 Prague Hybrid  Abstracts: <https://onlinelibrary.wiley.com/doi/epdf/10.1111/all.15614>  Oral presentations: <https://onlinelibrary.wiley.com/doi/epdf/10.1111/all.15615>  Posters: <https://onlinelibrary.wiley.com/doi/epdf/10.1111/all.15616> | 1 | 1 |
|  | - 2021 Madrid Digital - 2020 London Digital - 2019 Lisbon   <https://medialibrary.eaaci.org/mediatheque/results.aspx?channel=8518&search=%7B%22Text%22%3A%22%22%2C%22MediaType%22%3A%5B0%5D%2C%22ContentTypes%22%3A%5B%22abstract%22%2C%22e-poster%22%5D%2C%22SortBy%22%3A5%7D> | 1 (2019) | 0 |
| American Academy of Allergy, Asthma & Immunology (AAAAI) | 2022  <https://www.sciencedirect.com/journal/journal-of-allergy-and-clinical-immunology/vol/149/issue/2/suppl/S> | 0 | NA |
|  | 2021 Virtual  <https://www.jacionline.org/issue/S0091-6749(20)X0014-5> | 2 | 0 |
|  | 2020 Philadelphia  <https://plan.core-apps.com/tristar_aaaai20/abstracts> |  |  |
|  | 2019 San Francisco  <https://plan.core-apps.com/tristar_aaaai19/abstracts> |  |  |
| Clinical Immunology Society (CIS) North American Conference | 2022  <https://link.springer.com/article/10.1007/s10875-022-01216-6> | 4 | 3 |
|  | 2021  <https://link.springer.com/article/10.1007/s10875-021-01001-x> | 4 | 0 |
|  | 2020  <https://link.springer.com/article/10.1007/s10875-020-00764-z> | 9 | 6 |
|  | 2019  <https://link.springer.com/article/10.1007/s10875-019-00597-5> | 3 | 0 |
| American Society of Haematology (ASH) | 2022  <https://ashpublications.org/blood/issue/140/Supplement%201> | 2 | 1 |
|  | 2021  <https://ash.confex.com/ash/2021/webprogram/start.html> | 0 | 0 |
|  | 2020  <https://ashpublications.org/blood/issue/136/Supplement%201> | 11 | 0 |
|  | 2019  <https://ashpublications.org/blood/issue/134/Supplement_1> | 23 | 0 |
| International Primary Immunodeficiencies Congress (IPIC) | 2022 5th edition  Titles: <https://ipic2021.com/Abstracts-for-poster-presentation/>  Presentations: <https://ipic2021.com/scientific-programme/#1599742447962-117167c7-3a1a> | 2 | 2 |
|  | 2019 4th edition  <https://ipic2019.com/submit-your-abstract/> | 0 | 0 |
| International Congress of Immunology (IUIS) | 2019  <https://onlinelibrary.wiley.com/toc/15214141/2019/49/S3> | 0 | 0 |
| European Hematology Association (EHA) | 2022  <https://journals.lww.com/hemasphere/Fulltext/2022/06003/Abstract_Book_for_the_27th_Congress_of_the.1.aspx> | 0 | NA |
|  | 2021  2020  2019  <https://library.ehaweb.org/eha/#!*menu=6*browseby=3*sortby=2*ce_id=1766*featured=16775> | 2021: 0  2020: 1  2019: 0 | 0 |
| Total | **Total hits:** 65 | **Total results:** 14 | |

**Footnotes**: ^a^Records were assessed for inclusion across the clinical and economic SLRs.

**Abbreviations**: AAAAI: American Academy of Allergy, Asthma & Immunology; ASH: American Society of Haematology; APDS: activated PI3Kδ syndrome; CIS: Clinical Immunology Society North American Conference; EAACI: European Academy of Allergy and Clinical Immunology; EHA: European Haematology Association; ESID: European Society for Immunodeficiencies; ISPOR: International Society for Pharmacoeconomics and Outcomes Research; IPIC: International Primary Immunodeficiencies Congress; IUIS: International Congress of Immunology; NA: not applicable; PI3: Phosphoinositide 3; SLR: systematic literature review.

*Clinical Trial Registry Searches*

**Supplementary Table 37**: Clinical trial registry searches (clinical and economic SLRs)

| **Registry** | **Search strategy** | **Hits^a^** | **Records included^a^** |
| --- | --- | --- | --- |
| Clinicaltrials.gov | **Condition**: Supplementary search keywords noted above  **Other terms**: None  **Study type**: All studies  **Study results**: All studies  **Recruitment status**: All | 2021:132  2023: 141 | 2021: 0  2023: 0 |
| Clinicaltrialsregister.eu | Keywords as above | 2021: 5  2023: 6 | 2021: 0  2023: 0 |
| World Health Organisation (WHO) International Clinical Trials Registry Platform (ICTRP) | Keywords as above | 2021: 0  2023: 10 | 2021: 0  2023: 0 |

**Footnotes**: ^a^Records were assessed for inclusion across the clinical and economic SLRs.

**Abbreviations**: ICTRP: International Clinical Trials Registry Platform; SLR: systematic literature review; WHO: World Health Organisation.

*HTA Agency Website Searches*

**Supplementary Table 38**: HTA agency searches (clinical and economic SLRs)

| **HTA body** | **Hits (2021)^a^** | **Records included (2021)^a^** | **Hits (2023)^a^** | **Records included (2023)^a^** |
| --- | --- | --- | --- | --- |
| NICE  <https://www.nice.org.uk/> | 54 | 0 | 5 | 0 |
| Scottish Medicines Consortium (SMC) <https://www.scottishmedicines.org.uk/> |  |  | 0 | NA |
| All Wales Medicines Strategy Group (AWMSG)  <http://www.awmsg.org/> |  |  | 79 | 0 |
| **National Centre for Pharmacoeconomics (NCPE)^b^**  [**https://www.ncpe.ie/**](https://www.ncpe.ie/) | Not searched | | 0 | 0 |

**Footnotes**: ^a^Records were assessed for inclusion across the clinical and economic SLRs. ^b^All text in bold represents additional criteria introduced in the SLR update.

**Abbreviations**: AWMSG: All Wales Medicines Strategy Group; HTA: health technology assessment; NA: not applicable; NCPE: National Centre for Pharmacoeconomics; NICE: National Institute for Health and Care Excellence; SLR: systematic literature review; SMC: Scottish Medicines Consortium.

*Economic Website Searches*

**Supplementary Table 39**: Search terms and results of economic website searching (economic SLR update)

| **Source** | **Link** | **Search strategy** | **Search terms** | **Results**  **23rd June 2023** |
| --- | --- | --- | --- | --- |
| The Cost-Effectiveness Analysis (CEA) Registry, managed by Tufts Medical Center | <http://healtheconomicsdev.tuftsmedicalcenter.org/cear2/search/search.aspx> | Using the search bar, each search term in turn was searched, with Methods, Ratios and Utility Weights selected in turn; abstracts were reviewed for relevance | APDS | 0 hits |
|  |  |  | Activated phosphoinositide 3-kinase delta syndrome | 0 hits |
|  |  |  | Activated phosphoinositide 3-kinase (PI3) | 0 hits |
|  |  |  | p110 delta activating mutation causing senescent T cells, lymphadenopathy and immunodeficiency (PASLI) | 0 hits |
| The EQ-5D Publications Database | <http://eq-5dpublications.euroqol.org/?noheader=true> | Using the advanced search function, the search terms were combined with each of the following economic terms in turn and searched in the abstract:   - Cost - Economic - Utility - Utilities - Quality of life - Resource   Abstracts were reviewed for relevance | APDS | 0 hits |
|  |  |  | Activated phosphoinositide 3-kinase delta syndrome | 0 hits |
|  |  |  | Activated PI3 | 0 hits |
|  |  |  | PASLI | 0 hits |
| All | - | - | - | Total hits: 0 |

**Abbreviations**: APDS: activated phosphoinositide 3-kinase delta syndrome; CEA: Cost-Effectiveness Analysis; EQ-5D: EuroQol 5-Dimension; PASLI: p110 delta activating mutation causing senescent T cells, lymphadenopathy and immunodeficiency; PI3: phosphoinositide 3-kinase; SLR: systematic literature review.

Supplementary Methods 2: Eligibility Criteria

**Supplementary Table 40**: Eligibility criteria for the clinical SLR^a^

| **Category** | **Inclusion criteria** | **Exclusion criteria** |
| --- | --- | --- |
| **Study design and publication type** | Study design:   - Randomised controlled trials - Interventional non-randomised controlled studies - Observational studies including prospective and retrospective cohorts and case studies or case series - **Chart reviews or databases analyses**   Publication type:   - Peer-reviewed journal articles presenting original research studies - Congress abstracts - **Letters (if they report primary research)** - Case studies/reports^b^ | - Animal studies - In-vitro studies - Editorials - Reviews - Letters - Comments - Notes - Erratum - **Narrative reviews** - **Guidelines** - **Economic evaluations** - **Cost and resource use studies** - **Trial protocols not reporting any outcomes** - **Conference reviews** - **Consensus pieces** |
|  | SLRs and network meta-analyses (NMAs) of relevant primary publications will be considered relevant at the title/abstract review stage and hand searched for relevant primary studies but will be excluded during the full-text review stage unless they themselves present primary research. | |
| **Population** | People with APDS/PASLI (types 1 and 2) | Any other population |
| **Intervention** | - Leniolisib - **Other PI3Kδ inhibitors** - HSCT - Immunosuppressive agents (including, but not limited, to corticosteroids, mTOR inhibitors and rituximab) - Antimicrobial, **antiviral and antifungal** therapies - Immunoglobulin replacement therapies - **Surgery (such as tonsillectomy) and other procedures** | Any other treatment |
| **Comparators** | Any or no treatment | No restriction |
| **Outcomes** | Clinical efficacy or effectiveness, including:   - Frequencies of infections and other disease complications - Biomarkers reflecting the systemic inflammatory components of the disease (C-reactive protein [CRP], lactate dehydrogenase [LDH], beta 2 microglobulin [B2M], ferritin, fibrinogen and erythrocyte sedimentation rate [ESR]) - **Immunophenotype measures (including lymphocyte counts** [such as naïve B cells], **immunoglobulin levels and cytokine and chemokine levels)** - Immune system function for example lymphoproliferation, including lymphadenopathy (lymph node size), organomegaly (spleen and liver volume size), infections, **use of IRT, HSCT and/or antimicrobials** - Work Productivity Activity Impairment plus Classroom Impairment Questionnaire (WPAI-CIQ) - HRQoL including: **SF-36**, Physician's Global Assessment (PGA) and the PtGA - **Fatigue** - **Disease severity** - **Mortality** - **Lymphoma** - **Gastrointestinal manifestations** - **Cytopenia** - **Bronchiectasis (in relation to lung function)** - **Hearing loss** - **Protein S6K1 (S6) and phosphorylated protein kinase B (pAKT)**   Safety   - Adverse effects of treatment - Mortality | Studies that *only* report outcomes not relevant to the clinical and safety outcomes associated with treatment of APDS |
| **Geographical location** | No restriction | No restriction |
| **Language**^c^ | No restriction | No restriction |
| **Publication date** | No restriction; any study date | No restriction |

**Footnotes:** ^a^All text in bold represents additional criteria introduced in the clinical SLR update. ^b^Case reports/studies were included in the clinical SLR but not extracted, given the limited relevant information they provided. ^c^Studies with full texts not in English were translated using Google Translate; if this was not possible, they were screened based on their abstracts.

**Abbreviations:** APDS: activated PI3Kδ syndrome; B2M: beta 2 microglobulin; CRP: C-reactive protein; ESR: erythrocyte sedimentation rate; HRQoL: health-related quality of life; HSCT: haematopoietic stem cell transplantation; IRT: immunoglobulin replacement therapy; LDH: lactate dehydrogenase; mTOR: mammalian target of rapamycin; NMA: network meta-analysis; pAKT: phosphorylated protein kinase B; PASLI: p110 delta activating mutation causing senescent T cells, lymphadenopathy and immunodeficiency; PGA: Physician's Global Assessment; PtGA: Patient's Global Assessment; S6: protein S6K1; SF-36: short-form survey 36; SLR: systematic literature review; WPAI-CIQ: Work Productivity Activity Impairment plus Classroom Impairment Questionnaire.

**Supplementary Table 41**: Eligibility criteria for the epidemiology SLR

| **Category** | **Inclusion** | **Exclusion** |
| --- | --- | --- |
| **Study design** | - Any primary publication in humans | - Animal studies - In-vitro studies - Editorials - Reviews - Letters - Comments - Notes - Erratum   SLRs will be included at the abstract review stage, for handsearching of the reference lists, then excluded as primary publications. |
| **Population** | People with APDS/PASLI | Any other population |
| **Intervention** | Any or no treatment | No restriction |
| **Comparators** | Any or no treatment | No restriction |
| **Outcomes** | - Incidence of APDS - Prevalence of APDS | Any other outcomes |
| **Geographical location** | No restriction | No restriction |
| **Language** | No restriction | No restriction |
| **Publication date** | No restriction; any study date | No restriction |

**Abbreviations:** APDS: activated PI3Kδ syndrome; PASLI: p110 delta activating mutation causing senescent T cells, lymphadenopathy and immunodeficiency; SLR: systematic literature review.

**Supplementary Table 42**: Eligibility criteria for the economic SLR^a^

| Category | Inclusion criteria | Exclusion criteria |
| --- | --- | --- |
| Study design and publication type | - Any primary studies containing resource use or cost data (**including budget impact models and cost-of-illness studies**) - Economic evaluations, including economic models (cost effectiveness analyses, cost-utility analyses, cost-benefit analyses, cost-minimisation analyses) - HTAs - Congress abstracts - Letters (if they report primary research) - Case studies/reports | - Animal studies - In-vitro studies - Editorials - Reviews - Letters - Comments - Notes - Erratum - **Book chapters** |
|  | Relevant SLRs, network meta-analyses, HTAs, economic evaluations would be considered relevant at the title/abstract review stage and would be hand-searched for primary studies. They would be excluded at the full text review stage unless they presented primary research. | |
| Population | - People with APDS/PASLI (types 1 and 2) - Caregivers of people with APDS | Any other population |
| Intervention | No restriction | No restriction |
| Comparators | No restriction | No restriction |
| Outcomes | **Economic evaluation outcomes, including, but not limited to:**   - Quality-adjusted life years (QALYs) - Disability-adjusted life years (DALYs) - Incremental cost-effectiveness ratio (ICER) - Incremental cost-utility ratio (ICUR) - Life-years gained (LYG) - **Cost per clinical outcome** - **Cost per death averted** - **Total costs** - **Incremental cost**   **Costs and resource use outcomes, including but not limited to:**   - Cost of illness including average annual costs per person, cost of health care and social care, cost of the disease, treatment cost, healthcare resource utilisation, clinician cost - Rate of use of resources (e.g. hospitalisations, office visits, accident and emergency (A&E) visits, surgeries or **HSCT avoided**) - Indirect costs including out of pocket costs and average annual indirect cost per patient/caregiver, cost to the patient/caregiver, **productivity loss, work/school attendance** | Studies that *only* report outcomes not relevant to the cost and resource use associated with APDS |
| Geographical location | No restriction | No restriction |
| Language^b^ | No restriction | No restriction |
| Publication date | No restriction; any study date | No restriction |

**Footnote:** ^a^All text in bold represents additional criteria introduced in the SLR update. ^b^Studies with full texts not in English were translated using Google Translate; if this was not possible, they were screened based on their abstracts. **Abbreviations:** A&E: accident and emergency; APDS: activated PI3Kδ syndrome; DALY: disability-adjusted life years; HSCT: haematopoietic stem cell transplantation; HTA: health technology assessment; ICER: incremental cost-effectiveness ratio; ICUR: incremental cost-utility ratio; LYG: life-years gained; PASLI: p110 delta activating mutation causing senescent T cells, lymphadenopathy and immunodeficiency; QALY: quality-adjusted life years; SLR: systematic literature review.

**Supplementary Table 43**: Eligibility criteria for the HRQoL/utility studies

| **Category** | Inclusion criteria | Exclusion criteria |
| --- | --- | --- |
| **Study design and publication type** | Any primary research study in humans, including, but not limited to:   - Economic evaluations (cost-utility, cost-effectiveness, cost-consequence, cost-benefit, cost-minimisation) - HTAs - Congress abstracts - Letters (if they report primary research) - Case studies/reports | - Animal studies - In-vitro studies - Editorials - Reviews - Letters - Comments - Notes - Erratum - Guidelines - **Book chapters** |
|  | Relevant SLRs, network meta-analyses, HTAs, economic evaluations would be considered relevant at the title/abstract review stage and would be hand-searched for primary studies. They would be excluded at the full text review stage unless they presented primary research. | |
| **Population** | - People with APDS/PASLI (types 1 and 2) - Caregivers of people with APDS | Any other population |
| **Intervention** | No restriction | No restriction |
| **Comparators** | No restriction | No restriction |
| **Outcomes** | - Utilities including but not limited to: directly elicited (time trade-off [TTO], standard gamble [SG]) or generic preference-based utilities (e.g. EQ-5D, SF-6D, health utilities index [HUI], quality of wellbeing scale [QWB]) for relevant health states - Utilities and disutilities related to treatment and non-treatment related adverse events (AEs) - HRQoL measures (for physicians, patients and caregivers), including but not limited to: PGA, WPAI-CIQ, PtGA, SF-36 | Studies that *only* report outcomes not relevant to the utilities and HRQoL associated with APDS |
| **Geographical location** | No restriction | No restriction |
| **Language^b^** | No restriction | No restriction |
| **Publication date** | No restriction; any study date | No restriction |

**Footnote:** ^a^All text in bold represents additional criteria introduced in the SLR update. ^b^Studies with full texts not in English were translated using Google Translate; if this was not possible, they were screened based on their abstracts. **Abbreviations:** AEs: adverse events; APDS: activated PI3Kδ syndrome; EQ-5D: EurolQol-5 Dimensions; HRQoL: health-related quality of life; HTA: health technology assessment; HUI: Health Utilities Index; PASLI: p110 delta activating mutation causing senescent T cells, lymphadenopathy and immunodeficiency; PGA: Physician Global Assessment; PtGA: Patient’s Global Assessment; QWB: Quality of Wellbeing Scale; SF-36: Short Form 36; SF-6D: Short Form Six Dimensions; SG: standard gamble; SLR: systematic literature review; TTO: time trade-off; WPAI-CIQ: Work Productivity and Activity Impairment Questionnaire Plus Classroom Impairment Questionnaire.

Supplementary Results 1: Quality Assessment

**Table 44:** Quality assessments of studies included in the clinical SLR, assessed using the Downs and Black checklist4

| **Study name** | **Angulo 2013** | | **Avery 2018** | | **Begg 2023** | |
| --- | --- | --- | --- | --- | --- | --- |
| **Checklist item** | **Response*** | **How is the question addressed?** | **Response*** | **How is the question addressed?** | **Response*** | **How is the question addressed?** |
| 1. Is the hypothesis/aim/objective of the study clearly described? | Yes | The aim of the study is clearly stated. | Yes | The aim of the study is clearly stated. | Yes | The aim of the study is clearly stated. |
| 2. Are the main outcomes to be measured clearly described in the Introduction or Methods section? | Yes | Outcomes are clearly described in the methods. | Yes | Outcomes are clearly described in the methods. | Yes | Outcomes are clearly described in the methods. |
| 3. Are the characteristics of the patients included in the study clearly described? | Yes | Detailed characteristics of the included patients are given in the supplementary materials. | No | Limited information on inclusion and exclusion criteria and baseline characteristics is given. | Yes | Inclusion and exclusion criteria are provided. Baseline characteristics (including age, gender and APDS mutation) are reported. |
| 4. Are the intervention(s) of interest clearly described? | No | Limited information about the intervention is given. | No | Limited information about the intervention is given. | Yes | The intervention, dose and route of administration are clearly described. |
| 5. Are the distributions of principal confounders in each group of subjects to be compared clearly described? | No | A list of principal confounders is not given. | No | A list of principal confounders is not given. | No | A list of principal confounders is not given. |
| 6. Are the main findings of the study clearly described? | No | Limited detail on treatment outcomes are given. | Yes | The findings for outcomes stated in the methods are clearly described. | Yes | The findings for all outcomes stated in the methods are clearly described, numerically or visually. |
| 7. Does the study provide estimates of the random variability in the data for the main outcomes? | No | No estimates of the random variability are given. | Yes | Estimates of the random variability are reported for the main outcomes. | No | Estimates of the random variability are not reported for measurements of inflammatory markers and T and B cell populations. |
| 8. Have all important adverse events that may be a consequence of the intervention been reported? | No | AEs are not described. | No | AEs are not described. | Yes | AEs are clearly described in a table and in-text. |
| 9. Have the characteristics of patients lost to follow-up been described? | No | The study was not longitudinal. | NA | The study was not longitudinal; involved taking blood samples from patients at a single timepoint. | Yes | Numbers and reasons for discontinuation (and relationship to the intervention) are described. |
| 10. Have actual probability values been reported (e.g. 0.035 rather than <0.05) for the main outcomes except where the probability value is less than 0.001? | No | No formal statistical analysis was performed for outcomes of interest. | No | Summary probability values rather than actual values are reported. | No | No formal statistical analysis was performed due to the limited sample size. |
| 11. Were the subjects asked to participate in the study representative of the entire population from which they were recruited? | Unable to determine | APDS patients were identified from various PID cohorts, but it is unclear if those cohorts were representative. | Unable to determine | The study does not report how participants were selected or the proportion of the source population from which the participants are derived. | Unable to determine | The study does not report how participants were selected or the proportion of the source population from which the participants are derived. |
| 12. Were those subjects who were prepared to participate representative of the entire population from which they were recruited? | Unable to determine | As above. No validation was reported. | Unable to determine | As above. No validation was reported. | Unable to determine | As above. No validation was reported. |
| 13. Were the staff, places, and facilities where the patients were treated, representative of the treatment the majority of patients receive? | Unable to determine | Details of these factors were not provided. | Unable to determine | Details of these factors were not provided. | No | Safety assessments and conditions in the research unit exceeded recommendations for the management of AEs associated with the class of treatment. |
| 14. Was an attempt made to blind study subjects to the intervention they have received? | No | This was an observational study. | No | This was an observational study. | No | This was an open-label study. |
| 15. Was an attempt made to blind those measuring the main outcomes of the intervention? | Unable to determine | Blinding of investigators not reported. | Unable to determine | Blinding of investigators not reported. | Unable to determine | Blinding of investigators not reported. |
| 16. If any of the results of the study were based on “data dredging”, was this made clear? | Unable to determine | It is not stated whether all outcomes were pre-specified. | Unable to determine | It is not stated whether all outcomes were pre-specified. | Unable to determine | It is not stated whether all outcomes were pre-specified. |
| 17. In trials and cohort studies, do the analyses adjust for different lengths of follow-up of patients, or in case-control studies, is the time period between the intervention and outcome the same for cases and controls? | NA | The study was not longitudinal. | NA | The study was not longitudinal. | Yes | Length of follow-up was comparable for all participants. |
| 18. Were the statistical tests used to assess the main outcomes appropriate? | Yes | No formal statistical analysis was performed for outcomes of interest | Yes | There is no evidence of bias in the statistical analyses. | Yes | No formal statistical analysis was performed due to the limited sample size. |
| 19. Was compliance with the intervention/s reliable? | Unable to determine | Compliance with the interventions is not reported. | Yes | The intervention (HSCT) was discrete. | Yes | Four out of five participants who completed the study completed all timepoints and visits. |
| 20. Were the main outcome measures used accurate (valid and reliable)? | Yes | The outcomes reported are adequately described. | Yes | The main outcome measures are clearly described. | Yes | Safety outcomes were comprehensive and adequately described. |
| 21. Were the patients in different intervention groups (trials and cohort studies) or were the cases and controls (case-control studies) recruited from the same population? | No | Cases and controls were recruited from different populations. | Unable to determine | Details of participant recruitment are not provided. | Unable to determine | Details of participant recruitment are not provided. |
| 22. Were study subjects in different intervention groups (trials and cohort studies) or were the cases and controls (case-control studies) recruited over the same period of time? | Unable to determine | Details of the timing of participant recruitment are not provided. | Unable to determine | Details of participant recruitment are not provided. | Unable to determine | Details of participant recruitment are not provided. |
| 23. Were study subjects randomised to intervention groups? | No | Non-randomised observational study. | No | Non-randomised observational study comprising one intervention. | No | The study comprised only one intervention group. |
| 24. Was the randomised intervention assignment concealed from both patients and health care staff until recruitment was complete and irrevocable? | NA | Non-randomised observational study. | NA | Non-randomised observational study. | NA | Non-randomised study. |
| 25. Was there adequate adjustment for confounding in the analyses from which the main findings were drawn? | No | No adjustment for confounding was reported. | No | No adjustment for confounding was reported. | No | No adjustment for confounding was reported. |
| 26. Were losses of patients to follow-up taken into account? | NA | The study was not longitudinal. | NA | The study was not longitudinal. | Yes | Loss of participants to follow-up well described. |
| 27. Did the study have sufficient power to detect a clinically important effect where the probability value for a difference being due to chance is less than 5%? | No | No formal power analysis was reported. | No | No formal power analysis was reported. | No | No formal statistical analysis or power analysis was performed due to the limited sample size. |
| **Study name** | **Bloomfield 2021** | | **Campinhos 2021** | | **Chan 2020** | |
| **Checklist item** | **Response*** | **How is the question addressed?** | **Response*** | **How is the question addressed?** | **Response*** | **How is the question addressed?** |
| 1. Is the hypothesis/aim/objective of the study clearly described? | Yes | The aim of the study is clearly stated. | Yes | The aim of the study is clearly stated. | Yes | The aim of the study is clearly stated. |
| 2. Are the main outcomes to be measured clearly described in the Introduction or Methods section? | Yes | Outcomes are described in the methods. | No | Abstract only; limited information on the outcomes measured is given. | Yes | Outcomes are clearly described in the methods. |
| 3. Are the characteristics of the patients included in the study clearly described? | Yes | Baseline characteristics (including age, gender and APDS mutation) are reported. | No | Abstract only; limited information about patient characteristics is given. | No | Limited information about APDS patient characteristics is given. |
| 4. Are the intervention(s) of interest clearly described? | No | Dose and route/length of administration of each intervention are not described. | No | Dose and route/length of administration of each intervention are not described. | Yes | The intervention is clearly described. |
| 5. Are the distributions of principal confounders in each group of subjects to be compared clearly described? | No | A list of principal confounders is not given. | No | A list of principal confounders is not given. | No | A list of principal confounders is not given. |
| 6. Are the main findings of the study clearly described? | Yes | The main findings for outcomes stated in the methods are described narratively. | Yes | The findings for outcomes stated in the methods are clearly described numerically. | No | The main findings for APDS patients are unclear. |
| 7. Does the study provide estimates of the random variability in the data for the main outcomes? | No | No estimates of the random variability are given. | No | No estimates of the random variability are given. | No | No estimates of the random variability are given. |
| 8. Have all important adverse events that may be a consequence of the intervention been reported? | No | Some AEs reported but not systematically for each intervention. | No | Abstract only; limited information of AEs is reported. | No | No AEs are reported. |
| 9. Have the characteristics of patients lost to follow-up been described? | NA | This was a retrospective study. | Yes | No patients were lost to follow-up. | NA | The study comprises survey data taken at a single timepoint. |
| 10. Have actual probability values been reported (e.g. 0.035 rather than <0.05) for the main outcomes except where the probability value is less than 0.001? | No | No probability values are given. | No | No probability values are given. | No | No probability values are given. |
| 11. Were the subjects asked to participate in the study representative of the entire population from which they were recruited? | Unable to determine | The study reports on the source population from which the participants are derived but does not report how participants were selected from this population. | Unable to determine | The study does not report how participants were selected or the proportion of the source population from which the participants are derived. | Unable to determine | The study does not report the source population from which the participants are derived. |
| 12. Were those subjects who were prepared to participate representative of the entire population from which they were recruited? | Unable to determine | As above. No validation was reported. | Unable to determine | As above. No validation was reported. | Unable to determine | As above. No validation was reported. |
| 13. Were the staff, places, and facilities where the patients were treated, representative of the treatment the majority of patients receive? | Unable to determine | Details of these factors were not provided. | Unable to determine | Details of these factors were not provided. | Unable to determine | Details of these factors were not provided. |
| 14. Was an attempt made to blind study subjects to the intervention they have received? | No | This was an observational chart review. | No | This was an observational chart review. | No | This was an observational review of survey data. |
| 15. Was an attempt made to blind those measuring the main outcomes of the intervention? | No | This was an observational chart review. | No | This was an observational chart review. | No | This was an observational review of survey data. |
| 16. If any of the results of the study were based on “data dredging”, was this made clear? | Unable to determine | It is not stated whether all outcomes were pre-specified. | Unable to determine | It is not stated whether all outcomes were pre-specified. | Yes | No evidence of data dredging. |
| 17. In trials and cohort studies, do the analyses adjust for different lengths of follow-up of patients, or in case-control studies, is the time period between the intervention and outcome the same for cases and controls? | NA | This was an observational chart review. | NA | This was an observational chart review. | Unable to determine | No description of whether the analyses were adjusted for different lengths of follow-up. |
| 18. Were the statistical tests used to assess the main outcomes appropriate? | Yes | No formal statistical analysis was performed. | Yes | No formal statistical analysis was performed. | Yes | No formal statistical analysis was performed. |
| 19. Was compliance with the intervention/s reliable? | Unable to determine | Compliance with the interventions is not reported. | Unable to determine | Compliance with the interventions is not reported. | Yes | The intervention (HSCT) was discrete. |
| 20. Were the main outcome measures used accurate (valid and reliable)? | Yes | The outcomes reported are adequately described. | Yes | The outcomes reported are adequately described. | No | Reporting of the main outcomes is unclear. |
| 21. Were the patients in different intervention groups (trials and cohort studies) or were the cases and controls (case-control studies) recruited from the same population? | Unable to determine | Details of participant recruitment are not provided. | Unable to determine | Details of participant recruitment are not provided. | Unable to determine | Details of participant recruitment are not provided. |
| 22. Were study subjects in different intervention groups (trials and cohort studies) or were the cases and controls (case-control studies) recruited over the same period of time? | Unable to determine | Details of participant recruitment are not provided. | Unable to determine | Details of participant recruitment are not provided. | Unable to determine | Details of participant recruitment are not provided. |
| 23. Were study subjects randomised to intervention groups? | No | Non-randomised observational study. | No | Non-randomised observational study. | No | Non-randomised observational study. |
| 24. Was the randomised intervention assignment concealed from both patients and health care staff until recruitment was complete and irrevocable? | NA | Non-randomised observational study. | NA | Non-randomised observational study. | NA | Non-randomised observational study. |
| 25. Was there adequate adjustment for confounding in the analyses from which the main findings were drawn? | No | No adjustment for confounding was reported. | No | No adjustment for confounding was reported. | No | No adjustment for confounding was reported. |
| 26. Were losses of patients to follow-up taken into account? | NA | Retrospective observational study; no patients were lost to follow up. | NA | Retrospective observational study; no patients were lost to follow up. | NA | Observational study of survey data. |
| 27. Did the study have sufficient power to detect a clinically important effect where the probability value for a difference being due to chance is less than 5%? | No | No formal power analysis was performed. | No | No formal power analysis was performed. | No | No formal power analysis was performed. |
| **Study name** | **Conrey 2021** | | **Coulter 2017** | | **Diaz 2020** | |
| **Checklist item** | **Response*** | **How is the question addressed?** | **Response*** | **How is the question addressed?** | **Response*** | **How is the question addressed?** |
| 1. Is the hypothesis/aim/objective of the study clearly described? | Yes | The aim of the study is clearly stated. | Yes | The aim of the study is clearly stated. | Yes | The aim of the study is clearly stated. |
| 2. Are the main outcomes to be measured clearly described in the Introduction or Methods section? | No | Abstract only; only a brief description of outcomes to be measured is given. | Yes | Outcomes are described in the methods. | Yes | Outcomes are clearly described in the methods. |
| 3. Are the characteristics of the patients included in the study clearly described? | No | Abstract only: limited details of patient characteristics given. | Yes | Baseline characteristics (including age, gender and APDS mutation) are reported. | Yes | Inclusion and exclusion criteria are provided. Baseline characteristics (including age, gender and APDS mutation) are reported. |
| 4. Are the intervention(s) of interest clearly described? | No | Dose and route/length of administration of each intervention are not described. | No | Dose and route/length of administration of each intervention are not described. | Yes | The intervention, dose and route of administration are clearly described. |
| 5. Are the distributions of principal confounders in each group of subjects to be compared clearly described? | No | A list of principal confounders is not given. | No | A list of principal confounders is not given. | No | A list of principal confounders is not given. |
| 6. Are the main findings of the study clearly described? | No | The outcomes of leniolisib treatment are not clearly described. | No | The outcomes of each treatment are not clearly described. | Yes | The findings for all outcomes stated in the methods are clearly described, numerically or visually. |
| 7. Does the study provide estimates of the random variability in the data for the main outcomes? | No | Estimates of the random variability are not reported. | No | Estimates of the random variability are not reported. | No | Estimates of the random variability are not reported for the main outcomes. |
| 8. Have all important adverse events that may be a consequence of the intervention been reported? | No | AEs are not described. | No | Some AEs reported, but not systematically for all interventions. | Yes | AEs are clearly described in-text. |
| 9. Have the characteristics of patients lost to follow-up been described? | NA | This was a retrospective study. | NA | Retrospective chart review. | Yes | Numbers and reasons for discontinuation are described. |
| 10. Have actual probability values been reported (e.g. 0.035 rather than <0.05) for the main outcomes except where the probability value is less than 0.001? | No | No probability values are given. | No | No probability values are given. | No | No probability values are given. |
| 11. Were the subjects asked to participate in the study representative of the entire population from which they were recruited? | Unable to determine | The study does not report how participants were selected or the proportion of the source population from which the participants are derived. | Unable to determine | The study does not report how participants were selected or the proportion of the source population from which the participants are derived. | Unable to determine | The study does not report how participants were selected or the proportion of the source population from which the participants are derived. |
| 12. Were those subjects who were prepared to participate representative of the entire population from which they were recruited? | Unable to determine | As above. No validation was reported. | Unable to determine | As above. No validation was reported. | Unable to determine | As above. No validation was reported. |
| 13. Were the staff, places, and facilities where the patients were treated, representative of the treatment the majority of patients receive? | Unable to determine | Details of these factors were not provided. | Unable to determine | Details of these factors were not provided. | Unable to determine | Details of these factors were not provided. |
| 14. Was an attempt made to blind study subjects to the intervention they have received? | No | This was an observational cohort study. | No | This was an observational chart review. | No | This was an open-label study. |
| 15. Was an attempt made to blind those measuring the main outcomes of the intervention? | Unable to determine | Limited details on study design are given. | No | This was an observational chart review. | Unable to determine | Blinding of investigators not reported. |
| 16. If any of the results of the study were based on “data dredging”, was this made clear? | Unable to determine | It is not stated whether all outcomes were pre-specified. | Unable to determine | It is not stated whether all outcomes were pre-specified. | Unable to determine | It is not stated whether all outcomes were pre-specified. |
| 17. In trials and cohort studies, do the analyses adjust for different lengths of follow-up of patients, or in case-control studies, is the time period between the intervention and outcome the same for cases and controls? | Unable to determine | Abstract only; study design unclear. | NA | This was an observational chart review. | Unable to determine | Whether the analyses adjusted for different lengths of follow-up in the extension study is not described. |
| 18. Were the statistical tests used to assess the main outcomes appropriate? | Yes | No formal statistical analysis was performed. | Yes | No formal statistical analysis was performed. | Yes | No formal statistical analysis was performed. |
| 19. Was compliance with the intervention/s reliable? | Unable to determine | Compliance with the interventions is not reported. | Unable to determine | Compliance with the interventions is not reported. | Unable to determine | Compliance with the intervention is not reported. |
| 20. Were the main outcome measures used accurate (valid and reliable)? | No | Abstract only; the main outcome measures are unclear. | Yes | Outcomes were adequately described. | Yes | All outcomes were comprehensive and adequately described. |
| 21. Were the patients in different intervention groups (trials and cohort studies) or were the cases and controls (case-control studies) recruited from the same population? | Unable to determine | Details of participant recruitment are not provided. | Unable to determine | Details of participant recruitment are not provided. | Unable to determine | Details of participant recruitment are not provided. |
| 22. Were study subjects in different intervention groups (trials and cohort studies) or were the cases and controls (case-control studies) recruited over the same period of time? | Unable to determine | Details of participant recruitment are not provided. | Unable to determine | Details of participant recruitment are not provided. | Unable to determine | Details of participant recruitment are not provided. |
| 23. Were study subjects randomised to intervention groups? | No | Non-randomised observational study. | No | Non-randomised observational study. | No | The study comprised only one intervention group. |
| 24. Was the randomised intervention assignment concealed from both patients and health care staff until recruitment was complete and irrevocable? | NA | Non-randomised observational study. | NA | Non-randomised observational study. | NA | Non-randomised study. |
| 25. Was there adequate adjustment for confounding in the analyses from which the main findings were drawn? | No | No adjustment for confounding was reported. | No | No adjustment for confounding was reported. | No | No adjustment for confounding was reported. |
| 26. Were losses of patients to follow-up taken into account? | Unable to determine | Patient numbers and follow-up not reported. | NA | Retrospective observational study. | No | Whether loss of participants to follow-up was taken into account for the analyses was not described. |
| 27. Did the study have sufficient power to detect a clinically important effect where the probability value for a difference being due to chance is less than 5%? | No | No formal power analysis was performed. | No | No formal power analysis was performed. | No | No formal power analysis was performed. |
| **Study name** | **Dimitrova 2020 (Dimitrova 2021)** | | **Elgizouli 2016** | | **Elkaim 2016** | |
| **Checklist item** | **Response*** | **How is the question addressed?** | **Response*** | **How is the question addressed?** | **Response*** | **How is the question addressed?** |
| 1. Is the hypothesis/aim/objective of the study clearly described? | Yes | The aim of the study is clearly stated. | Yes | The aim of the study is clearly stated. | Yes | The aim of the study is clearly stated. |
| 2. Are the main outcomes to be measured clearly described in the Introduction or Methods section? | Yes | The main outcomes to be measured are specified in the methods. | No | The main outcomes to be measured are not clear from the methods. | Yes | The main outcomes to be measured are specified in the introduction. |
| 3. Are the characteristics of the patients included in the study clearly described? | Yes | The characteristics of the patients included are clearly described. | Yes | The characteristics of the patients included are clearly described. | Yes | The characteristics of the patients included are clearly described. |
| 4. Are the intervention(s) of interest clearly described? | Yes | Thorough details about HSCT (e.g. conditioning regime, HSCT type, age at treatment, graft source) are given.. | No | Details of the dose, route/length of administration of the interventions are not clearly described. | No | Details of the dose, route/length of administration of the interventions are not clearly described. |
| 5. Are the distributions of principal confounders in each group of subjects to be compared clearly described? | No | A list of principal confounders is not given. | No | A list of principal confounders is not given. | No | A list of principal confounders is not given. |
| 6. Are the main findings of the study clearly described? | Yes | The main findings are clearly described in the text and in figures/tables.. | Yes | The main findings are clearly described in the text. | Yes | The main findings are clearly described. |
| 7. Does the study provide estimates of the random variability in the data for the main outcomes? | Yes | Estimates of the random variability are provided for some outcomes. | No | Estimates of the random variability are not provided. | No | Estimates of the random variability are not provided. |
| 8. Have all important adverse events that may be a consequence of the intervention been reported? | Yes | AEs are clearly described. | No | AEs are not clearly described. | No | AEs are not clearly described. |
| 9. Have the characteristics of patients lost to follow-up been described? | NA | This was a retrospective case series. | NA | Results relevant to this review are from a retrospective chart review. | NA | This was a retrospective study. |
| 10. Have actual probability values been reported (e.g. 0.035 rather than <0.05) for the main outcomes except where the probability value is less than 0.001? | No | Actual probability values are not consistently reported for all outcomes. | No | No probability values are reported. | No | No probability values are reported. |
| 11. Were the subjects asked to participate in the study representative of the entire population from which they were recruited? | Unable to determine | The study does not report how participants were selected or the proportion of the source population from which the participants are derived. | Unable to determine | The study does not report how participants were selected or the proportion of the source population from which the participants are derived. | Unable to determine | The study does not report how participants were selected or the proportion of the source population from which the participants are derived. |
| 12. Were those subjects who were prepared to participate representative of the entire population from which they were recruited? | Unable to determine | As above. No validation was reported. | Unable to determine | As above. No validation was reported. | Unable to determine | As above. No validation was reported. |
| 13. Were the staff, places, and facilities where the patients were treated, representative of the treatment the majority of patients receive? | Unable to determine | Details of these factors were not provided. | Unable to determine | Details of these factors were not provided. | Unable to determine | Details of these factors were not provided. |
| 14. Was an attempt made to blind study subjects to the intervention they have received? | No | Discrete intervention; blinding not feasible. | No | This was a retrospective study. | No | This was a retrospective study. |
| 15. Was an attempt made to blind those measuring the main outcomes of the intervention? | No | Non-randomised study of a discrete intervention. | No | This was a retrospective study. | No | This was a retrospective study. |
| 16. If any of the results of the study were based on “data dredging”, was this made clear? | Unable to determine | This was a retrospective study; it is unclear whether all outcomes described were pre-specified. | Unable to determine | This was a retrospective study; it is unclear whether all outcomes described were pre-specified. | Unable to determine | This was a retrospective study; it is unclear whether all outcomes described were pre-specified. |
| 17. In trials and cohort studies, do the analyses adjust for different lengths of follow-up of patients, or in case-control studies, is the time period between the intervention and outcome the same for cases and controls? | No | Some outcomes adjusted for different lengths of follow-up (e.g. transplant-related mortality, graft failure) and others did not (HSCT outcomes). | Unable to determine | It is unclear whether the time period between the intervention and outcomes is the same for all cases. | Unable to determine | It is unclear whether the time period between the intervention and outcomes is the same for all cases. |
| 18. Were the statistical tests used to assess the main outcomes appropriate? | Yes | No evidence of bias in the statistical analyses. | Yes | No formal statistical analysis was performed. | Yes | No formal statistical analysis was performed. |
| 19. Was compliance with the intervention/s reliable? | Yes | The intervention (HSCT) was discrete. | Unable to determine | Compliance with the intervention not reported. | Unable to determine | Compliance with the intervention not reported. |
| 20. Were the main outcome measures used accurate (valid and reliable)? | Yes | Outcomes were adequately described. | Yes | Outcomes were adequately described. | Yes | Outcomes were adequately described. |
| 21. Were the patients in different intervention groups (trials and cohort studies) or were the cases and controls (case-control studies) recruited from the same population? | Unable to determine | Details of participant recruitment are not provided. | Unable to determine | Details of participant recruitment are not provided. | Unable to determine | Details of participant recruitment are not provided. |
| 22. Were study subjects in different intervention groups (trials and cohort studies) or were the cases and controls (case-control studies) recruited over the same period of time? | Unable to determine | Details of participant recruitment are not provided. | Unable to determine | Details of participant recruitment are not provided. | Unable to determine | Details of participant recruitment are not provided. |
| 23. Were study subjects randomised to intervention groups? | No | Non-randomised study. | No | Non-randomised study. | No | Non-randomised study. |
| 24. Was the randomised intervention assignment concealed from both patients and health care staff until recruitment was complete and irrevocable? | NA | Non-randomised study. | NA | Non-randomised study. | NA | Non-randomised study. |
| 25. Was there adequate adjustment for confounding in the analyses from which the main findings were drawn? | No | No adjustment for confounding was reported. | No | No adjustment for confounding was reported. | No | No adjustment for confounding was reported. |
| 26. Were losses of patients to follow-up taken into account? | Yes | Losses of patients to follow-up were taken into accounts for overall survival and graft failure-free survival. | NA | This was an observational, non-longitudinal study. | NA | This was an observational, retrospective study. |
| 27. Did the study have sufficient power to detect a clinically important effect where the probability value for a difference being due to chance is less than 5%? | No | No formal power analysis was reported. | No | No formal power analysis was reported. | No | No formal power analysis was reported. |
| **Study name** | **Ferkrvand 2021** | | **Fox 2018** | | **Imai 2014** | |
| **Checklist item** | **Response*** | **How is the question addressed?** | **Response*** | **How is the question addressed?** | **Response*** | **How is the question addressed?** |
| 1. Is the hypothesis/aim/objective of the study clearly described? | Yes | The aim of the study is clearly stated. | Yes | The aim of the study is clearly stated. | No | The aim of the study is unclear. |
| 2. Are the main outcomes to be measured clearly described in the Introduction or Methods section? | Yes | Outcomes are clearly described in the methods. | Yes | Outcomes are clearly described in the methods. | No | Abstract only; limited information on outcomes is given. |
| 3. Are the characteristics of the patients included in the study clearly described? | Yes | Some inclusion and exclusion criteria are provided. Baseline characteristics are reported. | Yes | Some inclusion and exclusion criteria are provided. Baseline characteristics are reported. | No | Abstract only; limited information on patient characteristics is given. |
| 4. Are the intervention(s) of interest clearly described? | Yes | The intervention, dose and route of administration are clearly described. | Yes | The intervention and method of administration are clearly described. | No | Abstract only; limited information on the intervention is given. |
| 5. Are the distributions of principal confounders in each group of subjects to be compared clearly described? | No | A list of principal confounders is not given. | No | A list of principal confounders is not given. | No | A list of principal confounders is not given. |
| 6. Are the main findings of the study clearly described? | Yes | Brief outcomes are described. | Yes | The findings for all outcomes stated in the methods are clearly described. | Yes | Brief outcomes of the intervention are described. |
| 7. Does the study provide estimates of the random variability in the data for the main outcomes? | No | Estimates of the random variability are not given. | No | Estimates of the random variability are not given. | No | Estimates of the random variability are not given. |
| 8. Have all important adverse events that may be a consequence of the intervention been reported? | No | AEs are not reported. | Yes | AEs are described. | No | Abstract only; only some AEs are described. |
| 9. Have the characteristics of patients lost to follow-up been described? | Yes | Retrospective study; no patients were lost to follow-up. | No | Characteristics of patients lost to follow-up not described. | Yes | No patients were lost to follow-up. |
| 10. Have actual probability values been reported (e.g. 0.035 rather than <0.05) for the main outcomes except where the probability value is less than 0.001? | No | No probability values are reported for outcomes of interest. | Yes | Actual probability values are reported for most outcomes. | No | No probability values are reported. |
| 11. Were the subjects asked to participate in the study representative of the entire population from which they were recruited? | Unable to determine | The study does not report how participants were selected or the proportion of the source population from which the participants are derived. | Unable to determine | The study does not report how participants were selected or the proportion of the source population from which the participants are derived. | Unable to determine | The study does not report how participants were selected or the proportion of the source population from which the participants are derived. |
| 12. Were those subjects who were prepared to participate representative of the entire population from which they were recruited? | Unable to determine | As above. No validation was reported. | Unable to determine | As above. No validation was reported. | Unable to determine | As above. No validation was reported. |
| 13. Were the staff, places, and facilities where the patients were treated, representative of the treatment the majority of patients receive? | Unable to determine | Only the location of treatment is described. Details of other factors are not described. | Unable to determine | Only locations of treatment administration described. | Unable to determine | These factors are not described. |
| 14. Was an attempt made to blind study subjects to the intervention they have received? | No | This was a retrospective study. | No | Discrete intervention; blinding not feasible. | No | Discrete intervention; blinding not feasible. |
| 15. Was an attempt made to blind those measuring the main outcomes of the intervention? | No | This was a retrospective study. | NA | Non-randomised study of a discrete intervention. | NA | Non-randomised study of a discrete intervention. |
| 16. If any of the results of the study were based on “data dredging”, was this made clear? | Unable to determine | It is not stated whether all outcomes were pre-specified. | Unable to determine | It is not stated whether all outcomes were pre-specified. | Unable to determine | It is not stated whether all outcomes were pre-specified. |
| 17. In trials and cohort studies, do the analyses adjust for different lengths of follow-up of patients, or in case-control studies, is the time period between the intervention and outcome the same for cases and controls? | Unable to determine | It is unclear whether the time period between the intervention and outcomes is the same for all cases. | Yes | Outcomes are reported at the same time for all participants. | Unable to determine | It is unclear whether the time period between the intervention and outcomes is the same for all cases. |
| 18. Were the statistical tests used to assess the main outcomes appropriate? | Yes | No evidence of bias in the statistical analyses. | Yes | No evidence of bias in the statistical analyses. | Yes | No formal statistical analysis was performed. |
| 19. Was compliance with the intervention/s reliable? | Unable to determine | Compliance with the interventions is not reported. | NA | The intervention (HSCT) was discrete. | NA | The intervention (HSCT) was discrete. |
| 20. Were the main outcome measures used accurate (valid and reliable)? | Yes | Outcomes were adequately described. | Yes | Outcomes were comprehensive and adequately described. | Yes | Outcomes were adequately described. |
| 21. Were the patients in different intervention groups (trials and cohort studies) or were the cases and controls (case-control studies) recruited from the same population? | Unable to determine | Details of participant recruitment are not provided. | Unable to determine | Details of participant recruitment are not provided. | Unable to determine | Details of participant recruitment are not provided. |
| 22. Were study subjects in different intervention groups (trials and cohort studies) or were the cases and controls (case-control studies) recruited over the same period of time? | Unable to determine | Details of participant recruitment are not provided. | Unable to determine | Details of participant recruitment are not provided. | Unable to determine | Details of participant recruitment are not provided. |
| 23. Were study subjects randomised to intervention groups? | No | The study comprised only one intervention group. | No | The study comprised only one intervention group. | No | The study comprised only one intervention group. |
| 24. Was the randomised intervention assignment concealed from both patients and health care staff until recruitment was complete and irrevocable? | NA | Non-randomised study. | NA | Non-randomised study. | NA | Non-randomised study. |
| 25. Was there adequate adjustment for confounding in the analyses from which the main findings were drawn? | No | No adjustment for confounding was reported. | No | No adjustment for confounding was reported. | No | No adjustment for confounding was reported. |
| 26. Were losses of patients to follow-up taken into account? | Yes | Retrospective study; no participants were lost to follow-up. | NA | Only one patient with APDS is described. | Yes | No participants were lost to follow-up. |
| 27. Did the study have sufficient power to detect a clinically important effect where the probability value for a difference being due to chance is less than 5%? | No | No formal power analysis was reported. | No | No formal power analysis was reported. | No | No formal power analysis was reported. |
| **Study name** | **Lucas 2014a (Lucas 2014b)** | | **Maccari 2018** | | **Martinez 2014** | |
| **Checklist item** | **Response*** | **How is the question addressed?** | **Response*** | **How is the question addressed?** | **Response*** | **How is the question addressed?** |
| 1. Is the hypothesis/aim/objective of the study clearly described? | Yes | The study aim is clearly described. | Yes | The study aim is clearly described. | Yes | The study aim is clearly described. |
| 2. Are the main outcomes to be measured clearly described in the Introduction or Methods section? | Yes | The main outcomes are clearly described in the methods. | No | The main outcomes are not described in the introduction or methods section. | Yes | Abstract only; the main outcomes are partly described. |
| 3. Are the characteristics of the patients included in the study clearly described? | Yes | Baseline characteristics (including age, gender and APDS mutation) are reported. | Yes | Baseline characteristics (including age and APDS mutation) are reported. | No | Baseline characteristics are not reported. |
| 4. Are the intervention(s) of interest clearly described? | Yes | The intervention, dose and route of administration are clearly described. | No | The dose and route of administration are not clearly described for all interventions. | No | The interventions are unclear. |
| 5. Are the distributions of principal confounders in each group of subjects to be compared clearly described? | No | A list of principal confounders is not given. | No | A list of principal confounders is not given; although, differences in CT uptake is noted as a potential confounder in the discussion. | No | A list of principal confounders is not given. |
| 6. Are the main findings of the study clearly described? | Yes | Findings for the one participant treated with rapamycin are clearly described. | Yes | Relevant findings are clearly described. | Yes | Abstract only; relevant findings are clearly described. |
| 7. Does the study provide estimates of the random variability in the data for the main outcomes? | No | Estimates of the random variability are not reported. | No | Estimates of the random variability are not reported. | No | Estimates of the random variability are not reported. |
| 8. Have all important adverse events that may be a consequence of the intervention been reported? | No | No AEs are reported. | Yes | AEs of interventions are reported but not systematically. | No | No AEs are reported. |
| 9. Have the characteristics of patients lost to follow-up been described? | NA | Only one treated participant. | No | Characteristics of patients lost to follow-up have not been described. | NA | Non-longitudinal study. |
| 10. Have actual probability values been reported (e.g. 0.035 rather than <0.05) for the main outcomes except where the probability value is less than 0.001? | No | Probability values are not reported. | No | Probability values are not reported. | Yes | Actual probability values are reported. |
| 11. Were the subjects asked to participate in the study representative of the entire population from which they were recruited? | Unable to determine | The study does not report how participants were selected or the proportion of the source population from which the participants are derived. | Yes | Study participants are from an international registry; all patients with available data at time of analysis are included in this study. | Unable to determine | The study does not report how participants were selected or the proportion of the source population from which the participants are derived. |
| 12. Were those subjects who were prepared to participate representative of the entire population from which they were recruited? | Unable to determine | As above. No validation was reported. | Yes | All participants with available data in the registry are included in this study. | Unable to determine | As above. No validation was reported. |
| 13. Were the staff, places, and facilities where the patients were treated, representative of the treatment the majority of patients receive? | Yes | The study reports that all procedures were based on standard of care and established clinical guidelines were followed. | Unable to determine | Details of these factors were not provided. | NA | Not relevant to study design (study of blood samples taken from people with APDS). |
| 14. Was an attempt made to blind study subjects to the intervention they have received? | No | Non-randomised study. | No | Registry study. | No | Non-randomised study. |
| 15. Was an attempt made to blind those measuring the main outcomes of the intervention? | Unable to determine | Blinding of investigators not reported. | No | Registry study. | Unable to determine | Blinding of investigators not reported. |
| 16. If any of the results of the study were based on “data dredging”, was this made clear? | Unable to determine | It is not stated whether all outcomes were pre-specified. | Unable to determine | It is not stated whether all outcomes were pre-specified. | Unable to determine | It is not stated whether all outcomes were pre-specified. |
| 17. In trials and cohort studies, do the analyses adjust for different lengths of follow-up of patients, or in case-control studies, is the time period between the intervention and outcome the same for cases and controls? | NA | Only one treated participant. | Unable to determine | Adjustment for different lengths of follow-up in the patient cohort is not reported. | NA | No follow-up, study of blood samples taken from people with APDS. |
| 18. Were the statistical tests used to assess the main outcomes appropriate? | Yes | No statistical analysis was performed for the outcomes relevant to this review. | Yes | No statistical analysis was performed for the outcomes relevant to this review. | Yes | No evidence of bias in the statistical analysis. |
| 19. Was compliance with the intervention/s reliable? | Unable to determine | Compliance with the intervention not reported. | No | Compliance is reported for some but not all interventions. | Unable to determine | Compliance with the intervention not reported. |
| 20. Were the main outcome measures used accurate (valid and reliable)? | Yes | Outcomes were adequately described. | Yes | Outcomes were adequately described. | Yes | Outcomes were adequately described. |
| 21. Were the patients in different intervention groups (trials and cohort studies) or were the cases and controls (case-control studies) recruited from the same population? | Unable to determine | Details of participant recruitment are not provided. | Unable to determine | Details of participant recruitment are not provided. | Unable to determine | Details of participant recruitment are not provided. |
| 22. Were study subjects in different intervention groups (trials and cohort studies) or were the cases and controls (case-control studies) recruited over the same period of time? | Unable to determine | Details of participant recruitment are not provided. | Unable to determine | Details of participant recruitment are not provided. | Unable to determine | Details of participant recruitment are not provided. |
| 23. Were study subjects randomised to intervention groups? | No | The study comprised only one intervention. | No | Registry study. | No | Non-randomised study. |
| 24. Was the randomised intervention assignment concealed from both patients and health care staff until recruitment was complete and irrevocable? | NA | Non-randomised study. | NA | Registry study. | NA | Non-randomised study. |
| 25. Was there adequate adjustment for confounding in the analyses from which the main findings were drawn? | No | No adjustment for confounding was reported. | No | No adjustment for confounding was reported. | No | No adjustment for confounding was reported. |
| 26. Were losses of patients to follow-up taken into account? | NA | Only one treated participant. | Unable to determine | It is unclear whether losses of patients to follow-up were taken into account. | NA | Non-longitudinal study of blood samples taken from people with APDS. |
| 27. Did the study have sufficient power to detect a clinically important effect where the probability value for a difference being due to chance is less than 5%? | No | No formal power analysis was reported. | No | No formal power analysis was reported. | No | No formal power analysis was reported. |
| **Study name** | **Nademi 2020** | | **Okano 2019** | | **Qiu 2022** | |
| **Checklist item** | **Response*** | **How is the question addressed?** | **Response*** | **How is the question addressed?** | **Response*** | **How is the question addressed?** |
| 1. Is the hypothesis/aim/objective of the study clearly described? | Yes | The aim of the study is clearly stated. | Yes | The aim of the study is clearly stated. | Yes | The aim of the study is clearly stated. |
| 2. Are the main outcomes to be measured clearly described in the Introduction or Methods section? | No | Abstract only; outcomes are not described in the introduction or methods. | No | Outcomes are not clearly described in the introduction or methods. | No | The specific outcomes to be reported are not specified in the introduction or methods section. |
| 3. Are the characteristics of the patients included in the study clearly described? | Yes | Some characteristics of the patients included are given. | Yes | Baseline characteristics are provided. | Yes | No inclusion or exclusion criteria are listed (observational). Baseline characteristics (including age, sex and APDS mutation) are reported. |
| 4. Are the intervention(s) of interest clearly described? | Yes | Abstract only; details of HSCT regimens are described. | Yes | The intervention is clearly described. | No | The interventions that each individual received are shown in Figure 6, however, the dose, duration of treatment and additional concomitant therapies are unclear. |
| 5. Are the distributions of principal confounders in each group of subjects to be compared clearly described? | No | A list of principal confounders is not given. | No | A list of principal confounders is not given. | No | A list of principal confounders is not given. |
| 6. Are the main findings of the study clearly described? | Yes | The findings for all outcomes are adequately described. | Yes | The findings for all outcomes are clearly described. | No | Some findings are reported with numerical data, while others are described narratively. Outcomes for all individuals in each treatment group are unclear. |
| 7. Does the study provide estimates of the random variability in the data for the main outcomes? | No | Estimates of the random variability are not given. | No | Estimates of the random variability are not given. | No | Estimates of the random variability are not reported for the main outcomes. |
| 8. Have all important adverse events that may be a consequence of the intervention been reported? | Yes | Some AEs are described. | Yes | AEs are described. | No | The study did not report AEs for each treatment comprehensively. |
| 9. Have the characteristics of patients lost to follow-up been described? | NA | Retrospective study of registry data. | NA | Retrospective chart review. | Yes | Participants lost to follow-up due to death are described. |
| 10. Have actual probability values been reported (e.g. 0.035 rather than <0.05) for the main outcomes except where the probability value is less than 0.001? | No | Probability values are not reported. | No | Probability values are not reported. | No | No formal statistical analysis was performed on the outcomes of interest. |
| 11. Were the subjects asked to participate in the study representative of the entire population from which they were recruited? | Unable to determine | The study does not report how participants were selected or the proportion of the source population from which the participants are derived. | Unable to determine | Participant selection is reported but it is unclear what proportion of the source population the participants constitute. | Unable to determine | The study does not report how participants were selected or the proportion of the source population from which the participants are derived. |
| 12. Were those subjects who were prepared to participate representative of the entire population from which they were recruited? | Unable to determine | As above. No validation was reported. | Unable to determine | As above. No validation was reported. | Unable to determine | As above. No validation was reported. |
| 13. Were the staff, places, and facilities where the patients were treated, representative of the treatment the majority of patients receive? | Unable to determine | Details of these factors were not provided. | Unable to determine | Medical records were collected from two medical centres, but the staff, facilities and treatment of the patients are not described. | Unable to determine | Details of these factors were not provided. |
| 14. Was an attempt made to blind study subjects to the intervention they have received? | No | Retrospective study design. | No | Discrete intervention; blinding not feasible. | No | Retrospective study design. |
| 15. Was an attempt made to blind those measuring the main outcomes of the intervention? | Unable to determine | As above. | NA | Retrospective study of a discrete intervention. | Unable to determine | As above. |
| 16. If any of the results of the study were based on “data dredging”, was this made clear? | Unable to determine | It is not stated whether all outcomes were pre-specified. | Unable to determine | It is not stated whether all outcomes were pre-specified. | Unable to determine | As above, due to the retrospective study design, it is unclear which outcomes were specified prior. |
| 17. In trials and cohort studies, do the analyses adjust for different lengths of follow-up of patients, or in case-control studies, is the time period between the intervention and outcome the same for cases and controls? | No | The analyses are descriptive and do not adjust for different lengths of follow-up. | No | The analyses are descriptive and do not adjust for different lengths of follow-up. | Unable to determine | The length of follow-up for each individual is unclear. |
| 18. Were the statistical tests used to assess the main outcomes appropriate? | Yes | No formal statistical analysis was performed. | Yes | No evidence of bias in the statistical analyses. | Yes | No formal statistical analysis was performed on the outcomes of interest. |
| 19. Was compliance with the intervention/s reliable? | NA | The intervention (HSCT) was discrete. | NA | The intervention (HSCT) was discrete. | Unable to determine | Compliance with the interventions was not reported. |
| 20. Were the main outcome measures used accurate (valid and reliable)? | Yes | Outcomes were adequately described. | Yes | Outcomes were adequately described. | Unable to determine | The outcome measures used to describe the results were unclear. |
| 21. Were the patients in different intervention groups (trials and cohort studies) or were the cases and controls (case-control studies) recruited from the same population? | NA | Only one intervention group. | NA | Only one intervention group. | Unable to determine | No information concerning the source of individuals in the study is given. |
| 22. Were study subjects in different intervention groups (trials and cohort studies) or were the cases and controls (case-control studies) recruited over the same period of time? | Unable to determine | The time period of participant recruitment is not described. | Unable to determine | The time period of participant recruitment is not described. | Unable to determine | No information on study recruitment is given. |
| 23. Were study subjects randomised to intervention groups? | No | Only one intervention group. | No | Only one intervention group. | No | Clinical data were collected retrospectively. |
| 24. Was the randomised intervention assignment concealed from both patients and health care staff until recruitment was complete and irrevocable? | NA | Non-randomised retrospective study. | NA | Non-randomised retrospective study. | No | Clinical data were collected retrospectively. |
| 25. Was there adequate adjustment for confounding in the analyses from which the main findings were drawn? | No | No adjustment for confounding was reported. | No | No adjustment for confounding was reported. | No | The effect of confounders was not investigated. |
| 26. Were losses of patients to follow-up taken into account? | NA | Retrospective study. | NA | Retrospective study. | No | The number of individuals lost due to death is reported, however, the effect of these losses on the main outcomes is unclear. |
| 27. Did the study have sufficient power to detect a clinically important effect where the probability value for a difference being due to chance is less than 5%? | No | No formal power analysis was reported. | No | No formal power analysis was reported. | No | No formal power analysis was performed. |
| **Study name** | **Rao 2023a (Rao 2022b, Rao 2023b, Rao 2017a, Rao 2017b, NCT02435173)** | | **Rao 2022a (Rao 2018, NCT02859727)** | | **Ruiz-Garcia 2018 (Ruiz-Garcia 2017)** | |
| **Checklist item** | **Response*** | **How is the question addressed?** | **Response*** | **How is the question addressed?** | **Response*** | **How is the question addressed?** |
| 1. Is the hypothesis/aim/objective of the study clearly described? | Yes | The aim of the study is clearly stated. | Yes | The aim of the study is clearly stated. | Yes | The aim of the study is clearly stated. |
| 2. Are the main outcomes to be measured clearly described in the Introduction or Methods section? | Yes | Outcomes are clearly described in the methods. | Yes | Outcomes are clearly described in the methods. | Yes | Outcomes are clearly described in the methods. |
| 3. Are the characteristics of the patients included in the study clearly described? | Yes | Inclusion and exclusion criteria are provided. Baseline characteristics (including age, gender and APDS mutation) are reported. | No | Limited details of the patients are given as the records were abstracts only. | Yes | Baseline characteristics (including age, gender and APDS mutation) are reported. |
| 4. Are the intervention(s) of interest clearly described? | Yes | The intervention, dose and route of administration are clearly described. | Yes | Dosage and route of administration are well described. | No | The dose and route of administration are not described.. |
| 5. Are the distributions of principal confounders in each group of subjects to be compared clearly described? | Yes | Baseline IRT or glucocorticoid use were specified as covariates in the statistical analysis section, and baseline distributions provided. | No | A list of principal confounders is not given. | No | A list of principal confounders is not given. |
| 6. Are the main findings of the study clearly described? | Yes | The findings for all outcomes stated in the methods are clearly described, numerically or visually. | Yes | The findings for all outcomes stated in the methods are clearly described. | Yes | The findings for all relevant outcomes are described. |
| 7. Does the study provide estimates of the random variability in the data for the main outcomes? | Yes | Estimates of the random variability are reported for the primary outcomes. | Yes | Estimates of the random variability are provided for lymph node and spleen volumes. | No | Estimates of the random variability are not reported for relevant outcomes. |
| 8. Have all important adverse events that may be a consequence of the intervention been reported? | Yes | AEs are clearly described in a table and in-text. | Yes | AEs are clearly described. | No | AEs are not described. |
| 9. Have the characteristics of patients lost to follow-up been described? | Yes | No patients were lost to follow-up and a participant flow diagram is given. | Yes | Numbers and reasons for study discontinuation are described. | Yes | No patients were lost to follow-up. |
| 10. Have actual probability values been reported (e.g. 0.035 rather than <0.05) for the main outcomes except where the probability value is less than 0.001? | Yes | Actual probability values are reported for the primary outcomes. | Yes | Where reported, actual probability values are given. | NA | No statistical analysis was performed for the relevant outcomes. |
| 11. Were the subjects asked to participate in the study representative of the entire population from which they were recruited? | Unable to determine | The study does not report how participants were selected or the proportion of the source population from which the participants are derived. However, authors note that the population was representative of the broader APDS population. | Unable to determine | The study does not report how participants were selected or the proportion of the source population from which the participants are derived. | Unable to determine | The study does not report how participants were selected or the proportion of the source population from which the participants are derived. |
| 12. Were those subjects who were prepared to participate representative of the entire population from which they were recruited? | Unable to determine | As above. No validation was reported. | Unable to determine | As above. No validation was reported. | Unable to determine | As above. No validation was reported. |
| 13. Were the staff, places, and facilities where the patients were treated, representative of the treatment the majority of patients receive? | Unable to determine | Study setting and facilities were not reported. | Unable to determine | Details of these factors were not provided. | Unable to determine | Details of these factors were not provided. |
| 14. Was an attempt made to blind study subjects to the intervention they have received? | Yes | Triple blind study (Part II); participants were blind to the intervention. | No | This was an open-label study. | Unable to determine | Whether study subjects were blinded is not reported. |
| 15. Was an attempt made to blind those measuring the main outcomes of the intervention? | Yes | Triple blind study (Part II); investigators were blind to the intervention. | Unable to determine | Blinding of investigators not reported. | Unable to determine | Blinding of investigators not reported. |
| 16. If any of the results of the study were based on “data dredging”, was this made clear? | Yes | Outcomes presented correspond to those stated in the clinicaltrials.gov entry. | No | All outcomes are as stated in the methods and the clinicaltrials.gov record. | Unable to determine | It is not stated whether all outcomes were pre-specified. |
| 17. In trials and cohort studies, do the analyses adjust for different lengths of follow-up of patients, or in case-control studies, is the time period between the intervention and outcome the same for cases and controls? | Yes | Length of follow-up was the same for all participants. | Yes | Outcomes are reported at the same follow-up times for all participants. | Unable to determine | Length of follow-up not reported. |
| 18. Were the statistical tests used to assess the main outcomes appropriate? | Yes | Distribution of the data is not described therefore the statistical tests used are assumed to be appropriate. | Yes | There is no evidence of bias in the statistical analyses. | Yes | No formal statistical analysis was performed.. |
| 19. Was compliance with the intervention/s reliable? | Yes | All participants completed treatment. | Yes | Treatment discontinuations were reported and were low in number. | Unable to determine | Compliance with the intervention is not reported. |
| 20. Were the main outcome measures used accurate (valid and reliable)? | Yes | Safety and efficacy outcomes were comprehensive and adequately described. | Yes | The outcome measures are clearly described. | Yes | The outcome measures are adequately described. |
| 21. Were the patients in different intervention groups (trials and cohort studies) or were the cases and controls (case-control studies) recruited from the same population? | Unable to determine | Participant recruitment not described. | Unable to determine | Details of participant recruitment are not provided. | Unable to determine | Details of participant recruitment are not provided. |
| 22. Were study subjects in different intervention groups (trials and cohort studies) or were the cases and controls (case-control studies) recruited over the same period of time? | Unable to determine | Participant recruitment not described. | Unable to determine | Details of participant recruitment are not provided. | Unable to determine | Details of participant recruitment are not provided. |
| 23. Were study subjects randomised to intervention groups? | Yes | Participants were randomised to groups using an automated system. | No | The study comprised only one intervention group. | No | The study comprised only one intervention group. |
| 24. Was the randomised intervention assignment concealed from both patients and health care staff until recruitment was complete and irrevocable? | Yes | Triple blind study design. | NA | Non-randomised study. | NA | Non-randomised study. |
| 25. Was there adequate adjustment for confounding in the analyses from which the main findings were drawn? | No | Efficacy outcomes are reported in an analysis set that excluded patients with protocol deviations. This may have affected the final balance of covariates in each group. | No | No adjustment for confounding was reported. | No | No adjustment for confounding was reported. |
| 26. Were losses of patients to follow-up taken into account? | Yes | No participants were lost to follow-up. | Yes | Loss of participants to follow-up is well described. | Yes | No participants were lost to follow-up. |
| 27. Did the study have sufficient power to detect a clinically important effect where the probability value for a difference being due to chance is less than 5%? | Yes | Input data and methods for power analysis are reported. | Unable to determine | No formal power analysis was reported. | No | No formal power analysis was reported. |
|  | **Tang 2017** | | **Tessarin 2020 (Tessarin 2021)** | | **Uzel 2014** | |
| **Checklist item** | **Response*** | **How is the question addressed?** | **Response*** | **How is the question addressed?** | **Response*** | **How is the question addressed?** |
| 1. Is the hypothesis/aim/objective of the study clearly described? | Yes | The aim of the study is clearly stated. | Yes | The aim of the study is clearly stated. | No | The aim of the study is not clearly stated. |
| 2. Are the main outcomes to be measured clearly described in the Introduction or Methods section? | No | Abstract only; outcomes are not clearly described in the introduction or methods. | No | Outcomes are not clearly described in the introduction or methods. | No | Abstract only; outcomes are not clearly described in the introduction or methods. |
| 3. Are the characteristics of the patients included in the study clearly described? | No | Abstract only; inclusion and exclusion criteria and baseline characteristics are not provided. | Yes | Baseline characteristics are provided. | No | Abstract only; inclusion and exclusion criteria and baseline characteristics are not provided. |
| 4. Are the intervention(s) of interest clearly described? | No | Limited detail on the route of administration, dose and duration of treatment are given. | No | Limited detail on the route of administration, dose and duration of treatment are given. | Yes | Interventions, dose and duration of treatment are described. |
| 5. Are the distributions of principal confounders in each group of subjects to be compared clearly described? | No | A list of principal confounders is not given. | No | A list of principal confounders is not given. | No | A list of principal confounders is not given. |
| 6. Are the main findings of the study clearly described? | No | Abstract only; a limited description of the outcomes is given. | Yes | The findings for outcomes are clearly described. | No | Abstract only; a limited description of the outcomes is given. |
| 7. Does the study provide estimates of the random variability in the data for the main outcomes? | No | Estimates of the random variability are not given. | No | Estimates of the random variability are not given for intervention-related outcomes. | No | Estimates of the random variability are not given. |
| 8. Have all important adverse events that may be a consequence of the intervention been reported? | No | AEs are not described. | Yes | AEs are described. | No | AEs are not described. |
| 9. Have the characteristics of patients lost to follow-up been described? | NA | Retrospective chart review. | NA | Retrospective chart review. | No | Loss of patients to follow-up not described. |
| 10. Have actual probability values been reported (e.g. 0.035 rather than <0.05) for the main outcomes except where the probability value is less than 0.001? | No | Probability values are not reported. | No | Probability values are not reported for intervention-related outcomes. | No | Probability values are not reported. |
| 11. Were the subjects asked to participate in the study representative of the entire population from which they were recruited? | Unable to determine | Participants selection is reported but it is unclear what proportion of the source population the participants constitute. | Unable to determine | Participants selection is reported but it is unclear what proportion of the source population the participants constitute. | Unable to determine | The study does not report how participants were selected or the proportion of the source population from which the participants are derived. |
| 12. Were those subjects who were prepared to participate representative of the entire population from which they were recruited? | Unable to determine | As above. No validation was reported. | Unable to determine | As above. No validation was reported. | Unable to determine | As above. No validation was reported. |
| 13. Were the staff, places, and facilities where the patients were treated, representative of the treatment the majority of patients receive? | Unable to determine | Details of these factors were not provided. | Unable to determine | Medical records were collected from several medical centres, but the staff, facilities and treatment of the patients are not described. | Unable to determine | Details of these factors were not provided. |
| 14. Was an attempt made to blind study subjects to the intervention they have received? | Unable to determine | Blinding not reported. | Unable to determine | Blinding not reported. | No | Open-label study. |
| 15. Was an attempt made to blind those measuring the main outcomes of the intervention? | Unable to determine | Blinding of investigators not reported. | Unable to determine | Blinding of investigators not reported. | No | Open-label study. |
| 16. If any of the results of the study were based on “data dredging”, was this made clear? | Unable to determine | It is not stated whether all outcomes were pre-specified. | Unable to determine | It is not stated whether all outcomes were pre-specified. | Unable to determine | It is not stated whether all outcomes were pre-specified. |
| 17. In trials and cohort studies, do the analyses adjust for different lengths of follow-up of patients, or in case-control studies, is the time period between the intervention and outcome the same for cases and controls? | No | The analyses are descriptive and do not adjust for different lengths of follow-up. | No | The analyses are descriptive and do not adjust for different lengths of follow-up. | No | Adjustment for different lengths of follow-up not reported. |
| 18. Were the statistical tests used to assess the main outcomes appropriate? | Yes | No evidence of bias in the statistical analyses. | Yes | No evidence of bias in the statistical analyses. | Yes | No evidence of bias in the statistical analyses. |
| 19. Was compliance with the intervention/s reliable? | Unable to determine | Compliance with the intervention is not reported. | Unable to determine | Compliance with the intervention is not reported. | Unable to determine | Compliance with the intervention is not reported. |
| 20. Were the main outcome measures used accurate (valid and reliable)? | No | Intervention-related outcomes are not adequately described. | No | Intervention-related outcomes are not adequately described. | No | Abstract only; a limited description of the outcome measures used is given. |
| 21. Were the patients in different intervention groups (trials and cohort studies) or were the cases and controls (case-control studies) recruited from the same population? | Unable to determine | Details of participant recruitment are not provided. | Yes | All participants receiving interventions were recruited from the same medical centres and all had APDS-1. | Unable to determine | Details of participant recruitment are not provided. |
| 22. Were study subjects in different intervention groups (trials and cohort studies) or were the cases and controls (case-control studies) recruited over the same period of time? | Unable to determine | Details of participant recruitment are not provided. | Unable to determine | The time period of participant recruitment is not described. | Unable to determine | Details of participant recruitment are not provided. |
| 23. Were study subjects randomised to intervention groups? | NA | Non-randomised retrospective study. | NA | Non-randomised retrospective study, | No | Non-randomised open-label study. |
| 24. Was the randomised intervention assignment concealed from both patients and health care staff until recruitment was complete and irrevocable? | NA | Non-randomised retrospective study. | NA | Non-randomised retrospective study. | NA | Non-randomised open-label study. |
| 25. Was there adequate adjustment for confounding in the analyses from which the main findings were drawn? | No | No adjustment for confounding was reported. | No | No adjustment for confounding was reported. | No | No adjustment for confounding was reported. |
| 26. Were losses of patients to follow-up taken into account? | NA | Retrospective study. | NA | Retrospective study. | Unable to determine | Losses of patients to follow-up is not reported. |
| 27. Did the study have sufficient power to detect a clinically important effect where the probability value for a difference being due to chance is less than 5%? | No | No formal power analysis was reported. | No | No formal power analysis was reported. | No | No formal power analysis was reported. |
| **Study name** | **Wang 2018** | | **Wang 2022** | | **Wentink 2017** | |
| **Checklist item** | **Response*** | **How is the question addressed?** | **Response*** | **How is the question addressed?** | **Response*** | **How is the question addressed?** |
| 1. Is the hypothesis/aim/objective of the study clearly described? | Yes | The aim of the study is clearly stated. | Yes | The aim of the study is clearly stated. | Yes | The aim of the study is clearly stated. |
| 2. Are the main outcomes to be measured clearly described in the Introduction or Methods section? | Yes | Outcomes are clearly described in the methods. | Yes | Outcomes are clearly described in the methods. | Yes | The outcomes are clearly described in the methods. |
| 3. Are the characteristics of the patients included in the study clearly described? | Yes | Baseline characteristics are provided. | No | A case-definition and source for controls are not given. Baseline characteristics (including age, gender and APDS mutation) are reported. | Yes | Baseline characteristics are provided. |
| 4. Are the intervention(s) of interest clearly described? | No | Dose and route of administration are not clearly described for all interventions. | No | Interventions (including dose, route of administration) are not adequately described. | No | The dose, route of administration and duration of treatment are not described. |
| 5. Are the distributions of principal confounders in each group of subjects to be compared clearly described? | No | A list of principal confounders is not given. | No | A list of principal confounders is not given. | No | A list of principal confounders is not given. |
| 6. Are the main findings of the study clearly described? | Yes | The main findings are clearly described. | No | Some findings are reported with numerical data, while others are described narratively. Outcomes for all individuals in each treatment group are unclear. | Yes | The main findings are clearly described. |
| 7. Does the study provide estimates of the random variability in the data for the main outcomes? | No | Estimates of the random variability are not given for intervention-related outcomes. | No | Estimates of the random variability are not reported for measures of routine immunological features and lymphocyte subpopulations. | No | Estimates of the random variability are not given for intervention-related outcomes. |
| 8. Have all important adverse events that may be a consequence of the intervention been reported? | No | AEs are not described. | No | AEs are not described. | No | AEs are not described. |
| 9. Have the characteristics of patients lost to follow-up been described? | NA | Retrospective chart review. | Yes | Numbers and reasons for discontinuation (e.g. death) are described. | No | Loss of patients to follow-up not described. |
| 10. Have actual probability values been reported (e.g. 0.035 rather than <0.05) for the main outcomes except where the probability value is less than 0.001? | No | Probability values are not reported. | No | No actual probability values have been reported for the outcomes of interest. | No | Probability values are not reported for intervention-related outcomes. |
| 11. Were the subjects asked to participate in the study representative of the entire population from which they were recruited? | Unable to determine | The study does not report how participants were selected or the proportion of the source population from which the participants are derived. | Unable to determine | The study does not report how participants were selected or the proportion of the source population from which the participants are derived. | Unable to determine | The study does not report how participants were selected or the proportion of the source population from which the participants are derived. |
| 12. Were those subjects who were prepared to participate representative of the entire population from which they were recruited? | Unable to determine | As above. No validation was reported. | Unable to determine | As above. No validation was reported. | Unable to determine | As above. No validation was reported. |
| 13. Were the staff, places, and facilities where the patients were treated, representative of the treatment the majority of patients receive? | Unable to determine | Details of these factors were not provided. | Unable to determine | As above, the study did not report on the staff, places, and facilities. | Unable to determine | Details of these factors were not provided. |
| 14. Was an attempt made to blind study subjects to the intervention they have received? | Unable to determine | Blinding not reported. | No | This was an observational study. | Unable to determine | Blinding not reported. |
| 15. Was an attempt made to blind those measuring the main outcomes of the intervention? | Unable to determine | Blinding of investigators not reported. | Unable to determine | Blinding of investigators not reported. | Unable to determine | Blinding not reported. |
| 16. If any of the results of the study were based on “data dredging”, was this made clear? | Unable to determine | It is not stated whether all outcomes were pre-specified. | Unable to determine | It is not stated whether all outcomes were pre-specified. | Unable to determine | It is not stated whether all outcomes were pre-specified. |
| 17. In trials and cohort studies, do the analyses adjust for different lengths of follow-up of patients, or in case-control studies, is the time period between the intervention and outcome the same for cases and controls? | No | The analyses are descriptive and do not adjust for different lengths of follow-up. | Unable to determine | Time period between the intervention and outcome is not reported. | No | No adjustment for different lengths of follow-up is reported. |
| 18. Were the statistical tests used to assess the main outcomes appropriate? | Yes | No evidence of bias in the statistical analyses. | Yes | Little statistical analysis has been undertaken and there is no evidence of bias. | Yes | No evidence of bias in the statistical analyses. |
| 19. Was compliance with the intervention/s reliable? | Unable to determine | Compliance with the intervention is not reported. | Unable to determine | Compliance with the intervention not reported. | Unable to determine | Compliance with the intervention is not reported. |
| 20. Were the main outcome measures used accurate (valid and reliable)? | No | A limited description of the intervention-related outcomes used is given. | Yes | Outcome measures are clearly described. | No | A limited description of the outcome measures used is given. |
| 21. Were the patients in different intervention groups (trials and cohort studies) or were the cases and controls (case-control studies) recruited from the same population? | Unable to determine | Details of participant recruitment are not provided. | Unable to determine | Details of participant recruitment are not provided. | Unable to determine | Details of participant recruitment are not provided. |
| 22. Were study subjects in different intervention groups (trials and cohort studies) or were the cases and controls (case-control studies) recruited over the same period of time? | Unable to determine | Details of participant recruitment are not provided. | Unable to determine | Details of participant recruitment are not provided. | Unable to determine | Details of participant recruitment are not provided. |
| 23. Were study subjects randomised to intervention groups? | NA | Non-randomised retrospective study. | No | Non-randomised study. | No | Non-randomised study. |
| 24. Was the randomised intervention assignment concealed from both patients and health care staff until recruitment was complete and irrevocable? | NA | Non-randomised retrospective study. | No | Non-randomised study. | NA | Non-randomised study. |
| 25. Was there adequate adjustment for confounding in the analyses from which the main findings were drawn? | No | No adjustment for confounding was reported. | No | No adjustment for confounding was reported. | No | No adjustment for confounding was reported. |
| 26. Were losses of patients to follow-up taken into account? | NA | Retrospective study. | Yes | Loss of participants due to death was well described. | Unable to determine | Losses of patients to follow-up is not reported. |
| 27. Did the study have sufficient power to detect a clinically important effect where the probability value for a difference being due to chance is less than 5%? | No | No formal power analysis was reported. | No | No formal power analysis is reported. | No | No formal power analysis was reported. |

**Abbreviations:** AE: adverse event; APDS: activated phosphoinositide 3-kinase delta syndrome; HSCT: haematopoietic stem cell transplantation; IRT: immunoglobulin replacement therapy; NA: not applicable.

**Sources** (Please see the main body for the full reference list): Angulo 2013,[^3^](#_ENREF_3) Avery 2018,[^118^](#_ENREF_118) Begg 2023,[^119^](#_ENREF_119) Bloomfield 2021,[^120^](#_ENREF_120) Campinhos 2017,[^121^](#_ENREF_121) Chan 2020,[^122^](#_ENREF_122) Conrey 2021,[^123^](#_ENREF_123) Coulter 2017,[^7^](#_ENREF_7) Diaz 2020,[^124^](#_ENREF_124) Dimitrova 2020,[^125^](#_ENREF_125) Dimitrova 2021,[^149^](#_ENREF_149) Elgizouli 2016,[^126^](#_ENREF_126) Elkaim 2016,[^16^](#_ENREF_16) Fekrvand 2021,[^127^](#_ENREF_127) Fox 2018,[^128^](#_ENREF_128) Imai 2014,[^129^](#_ENREF_129) Lucas 2014,[^131^](#_ENREF_131) Lucas 2014,[^130^](#_ENREF_130) Maccari 2018,[^11^](#_ENREF_11) Martinez 2014,[^132^](#_ENREF_132) Nademi 2020,[^133^](#_ENREF_133) Okano 2019,[^134^](#_ENREF_134) Qiu 2022,[^135^](#_ENREF_135) Rao 2023a,[^22^](#_ENREF_22) Rao 2022b,[^151^](#_ENREF_151) Rao 2023b,[^151^](#_ENREF_151) Rao 2017a,[^9^](#_ENREF_9) Rao 2017b,[^152^](#_ENREF_152) NCT02435173, Rao 2022a,[^138^](#_ENREF_138) Rao 2018,[^136^](#_ENREF_136) NCT02859727, Ruiz-Garcia 2018,[^140^](#_ENREF_140) Ruiz-Garcia 2017,[^141^](#_ENREF_141) Tang 2017,[^147^](#_ENREF_147) Tessarin 2021,[^142^](#_ENREF_142) Tessarin 2020,[^143^](#_ENREF_143) Uzel 2014,[^144^](#_ENREF_144) Wang 2018,[^148^](#_ENREF_148) Wang 2022,[^145^](#_ENREF_145) Wentink 2017.[^146^](#_ENREF_146)

Supplementary Results 2: Economic SLR

**Supplementary Table 45:** Summary of the results of cost/HCRU studies included in the economic SLR

| Study name | Objective and population | Country, perspective and cost year | Cost valuations used in the study | Costs/resource use reported | Applicability to clinical practice in England and for cost-effectiveness analysis |
| --- | --- | --- | --- | --- | --- |
| Ariue 2020^32^ | Objective:  To describe the case presentation of an individual with APDS  Population:  A 20-year-old male with APDS1 receiving sirolimus and IRT | Country: US  Cost perspective: NA  Cost year: NA | Case review of a patient including some description of HCRU  Costs were not valued; only HCRU was reported | The individual presented to the Emergency Department with a 3-week complaint of right leg weakness, constipation and urinary retention  Length of hospital stay: 2 weeks (lumbar puncture performed by the Neurosurgery service)  Extensive hospital evaluation of his neurological deficits was conducted  Multiple magnetic resonance imaging (MRI) examinations conducted | Reports HCRU only; cost data not reported |
| Harrington 2023^150^ | Objective:  To estimate the annual direct medical costs associated with APDS  Population:  People with APDS who are assumed to be treated | Country: US  Perspective: NR  Cost year: 2022 | A conceptual model was developed, summarising both APDS-associated manifestations and medications to treat APDS  Annual rates of manifestations and treatment were estimated based on interim survey responses from experts currently treating people diagnosed with APDS and assumed to be treated  Survey responses were weighted based on the number of people with APDS treated by each respondent  To estimate costs for each manifestation and treatment, a targeted literature review was conducted  All costs were inflated to 2022 USD via the medical inflation index  These values were then used in the model to estimate average annual direct medical costs based on the minimum and maximum manifestation and treatment rates reported in the surveys | Total estimated average annual cost among people with APDS: $83,057–$793,620  Estimated average annual cost of manifestations of APDS: $12,242–$322,939  Infections, haematologic pathology and malignancy were the greatest drivers of costs due to annual APDS-manifestations  Estimated average annual treatment cost of APDS: $70,814–$470,681  IRT and HSCT were greatest contributors to annual treatment cost adding $37,963–$94,907 and $23,143–$289,293, respectively, to the average annual cost of care | The study was performed from a US perspective, which limits its relevance to clinical practice in England and NHS resource use  Characteristics of the patient population (e.g. age) are not specified and specific treatments are not reported |

**Abbreviations:** APDS: activated phosphoinositide 3-kinase delta syndrome; CT: computed tomography; HCRU: healthcare resource use; HSCT: haematopoietic stem cell transplantation; IRT: immunoglobulin replacement therapy; MRI: magnetic resonance imaging; NA: not applicable; NHS: National Health Service; NR: not reported; PI3K: phosphoinositide 3-kinase; SLR: systematic literature review; US: United States; USD: United States dollars.

**Source** (Please see the main body for the full reference list): Ariue 2020,^32^ Harrington 2023.^150^

Supplementary Results 3: HRQoL SLR

**Supplementary Table 46**: Summary of the results of HRQoL study included in the HRQoL SLR

| **Source** | **Description of population, any interventions and recruitment method** | **Country, setting and study type** | **Sample size, response rate** | **Health states and adverse events** | **Methods of elicitation, valuation and mapping** | **Utility values and uncertainty around values** | **Appropriateness of study for cost-effectiveness evaluation** |
| --- | --- | --- | --- | --- | --- | --- | --- |
| Rao 2017^9^ | Population:  Individuals with with an APDS-associated genetic PI3KD mutation and clinical findings compatible with APDS  APDS type  APDS1  Mutation  E525K: n=1  E1021K: n=5  Age  Age at enrolment (years): 17, 24, 17, 20, 25, 31  Gender  Female (n=2), male (n=4)  Intervention:  Escalating doses of leniolisib (10, 30 and 70 mg bid for 4 weeks each)  Recruitment:  Individuals were recruited from: the National Institute of Allergy and Infectious Diseases, NIH (n=4), Motol University Hospital, Prague, Czech Republic (n=1), Erasmus medical centre, Rotterdam, The Netherlands (n=1) | Country:  Multinational (USA, Belarus, Czech Republic, Germany, Ireland, Italy, The Netherlands, Russian Federation, UK)  Care setting:  NR  Study Type:  12-week, open-label, multi-centre, within-subject, dose-escalation clinical trial in 6 people with APDS | N=6  Evaluable HRQoL data was available for all patients | HRQoL reported for trial cohort every 4 weeks to Week 12  Utilities for specific AEs NR | The PGA and PtGA (100 mm VAS), describing self-reported APDS-related well-being, were measured every 4 weeks to Week 12  Textual patient narratives were provided for each patient by the investigator at the end of the trial | The PtGA VAS measuring self-reported APDS-related wellbeing showed a mean (standard deviation) increase in well-being of 11 (11) mm (range: –3–22 mm)  The PGA VAS demonstrated less disease activity following 12 weeks of treatment, with a mean (standard deviation) reduction of 26 (16) mm (range: 12–51 mm)  In the textual patient narratives, investigators described an improvement with treatment, including details on various clinical and quality of life improvements. Increased energy levels and/or decreased fatigue were consistently described following treatment (n=6) | Consistency with NICE reference case  HRQoL values are reported rather than utility values, which deviate from NICE’s preference  This was an international study, with participants from the UK. However, specific data are not presented for UK patients and it is unclear how many patients were from the UK. As such, the results may not be directly relevant to clinical practice in the UK  Relevance to decision problem  Individuals had APDS and were all adolescents or adults (≥12 years old) which is aligned to the decision problem |

**Abbreviations**: APDS: activated phosphoinositide 3-kinase delta (PI3Kδ) syndrome; bid: bis in die (twice in a day); HRQoL: health related quality of life; NICE: National Institute for Health and Care Excellence; NIH: National Institutes of Health; NR: not reported; PGA: Physician Global Assessment; PtGA: Patient Global Assessment; SLR: systematic literature review; UK: United Kingdom; US: United States; VAS: visual analogue scale.

**Source** (Please see the main body for the full reference list): Rao 2017.^9^
